# Supplementary material for: Phosphodiesterase 2A2 regulates mitochondria clearance through Parkin-dependent mitophagy
Source: Commun Biol. 2020 Oct 21;3:596. doi: 10.1038/s42003-020-01311-7 (PMC7578833; doi:10.1038/s42003-020-01311-7)

Supplementary Figures and Legends

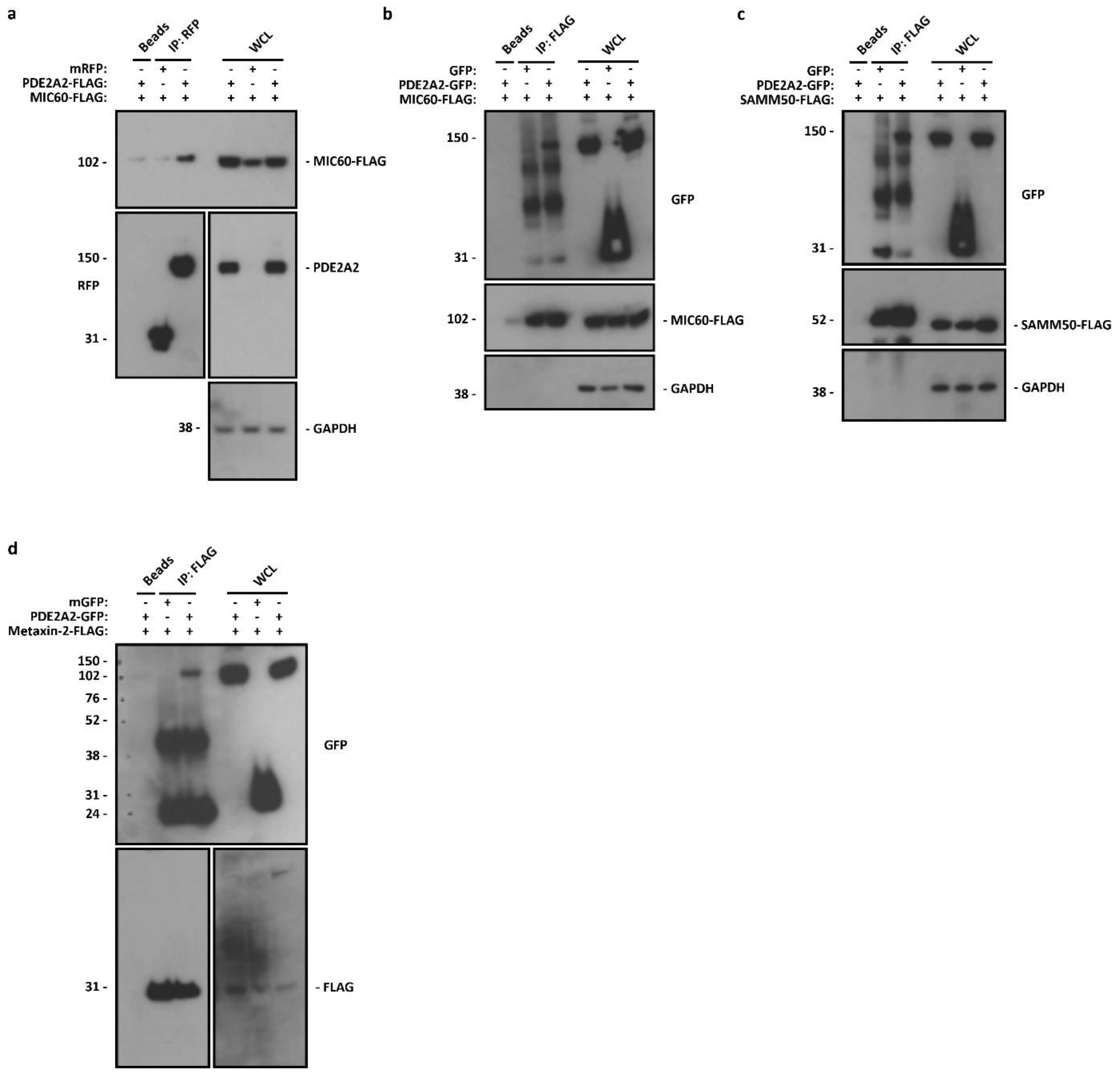

Supplementary Figure 1 | **PDE2A2 interacts with MICOS components.**

(**a**) Detection of MIC60-FLAG after PDE2A2-RFP or mRFP immunoprecipitation from lysates obtained from HEK293T cells transiently expressing MIC60-FLAG and either PDE2A2-RFP or mRFP. Pull-down of mRFP and pull-down with beads only (without conjugated antibody) were used as a negative control.

(**b**) Detection of PDE2A2-GFP and GFP after MIC60-FLAG immunoprecipitation from lysates obtained from HEK293T cells transiently expressing MIC60-FLAG and either PDE2A2-GFP or GFP.

(**c**) Detection of PDE2A2-GFP and GFP after SAMM50-FLAG immunoprecipitation from lysates obtained from HEK293T cells transiently expressing SAMM50-FLAG and either PDE2A2-GFP or GFP.

(**d**) Detection of PDE2A2-GFP and GFP after Metaxin-2-FLAG immunoprecipitation from lysates obtained from HEK293T cells transiently expressing Metaxin-2-FLAG and either PDE2A2-GFP or GFP. For all panels blot is representative of two independent experiments.

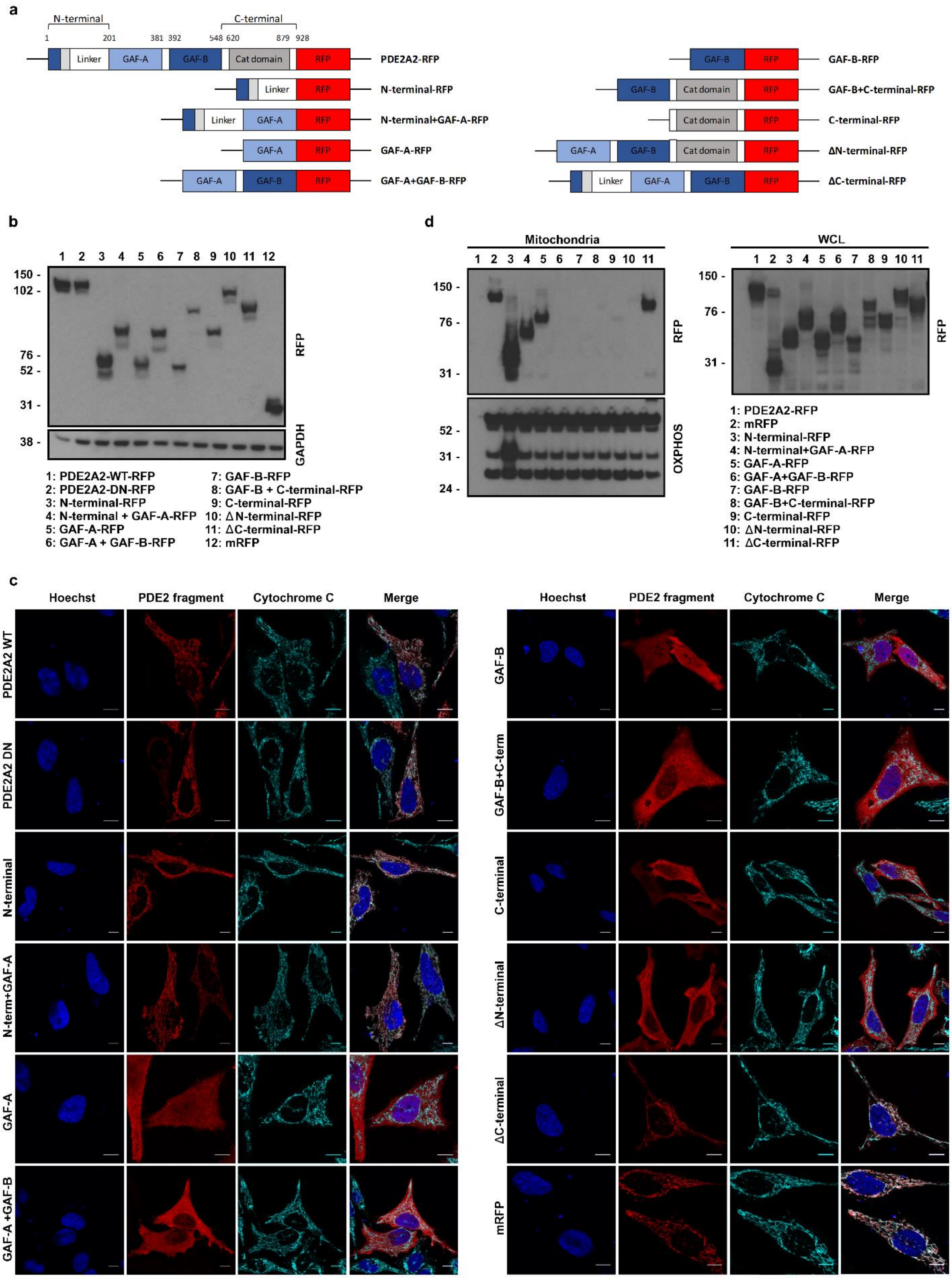
a
N-terminal
C-terminal
1 201 381 392 548 620 879 928
Linker GAF-A GAF-B Cat domain RFP
PDE2A2-RFP
N-terminal-RFP
N-terminal+GAF-A-RFP
GAF-A-RFP
GAF-A+GAF-B-RFP
GAF-B-RFP
GAF-B+C-terminal-RFP
C-terminal-RFP
ΔN-terminal-RFP
ΔC-terminal-RFP
b
RFP
GAPDH
1: PDE2A2-WT-RFP
2: PDE2A2-DN-RFP
3: N-terminal-RFP
4: N-terminal + GAF-A-RFP
5: GAF-A-RFP
6: GAF-A + GAF-B-RFP
7: GAF-B-RFP
8: GAF-B + C-terminal-RFP
9: C-terminal-RFP
10: ΔN-terminal-RFP
11: ΔC-terminal-RFP
12: mRFP
d
Mitochondria
WCL
RFP
OXPHOS
1: PDE2A2-RFP
2: mRFP
3: N-terminal-RFP
4: N-terminal+GAF-A-RFP
5: GAF-A-RFP
6: GAF-A+GAF-B-RFP
7: GAF-B-RFP
8: GAF-B+C-terminal-RFP
9: C-terminal-RFP
10: ΔN-terminal-RFP
11: ΔC-terminal-RFP
c
Hoechst
PDE2 fragment
Cytochrome C
Merge
PDE2A2 WT
PDE2A2 DN
N-terminal
N-term+GAF-A
GAF-A
GAF-A +GAF-B
GAF-B
GAF-B+C-term
C-terminal
ΔN-terminal
ΔC-terminal
mRFP

Supplementary Figure 2 | **The N-terminal region of PDE2A2 is necessary and sufficient to target the protein to mitochondria.**

(**a**) Schematic representation of the PDE2A2-RFP deletion constructs generated. Amino acid numbering refers to the mouse protein.

(**b**) western blot analysis showing the fragments molecular weight upon expression in HEK293T cells. GAF-A/B, cGMP-specific phosphodiesterases, adenylyl cyclases and FhlA domains A and B. Cat domain, catalytic domain. mRFP, mitochondrial RFP. PDE2A2-DN is a mutant containing a single amino acid substitution in the catalytic site resulting in a catalytically inactive enzyme[62]. Blot is representative of two independent experiments.

(**c**) Confocal images of HeLa cells expressing PDE2A2-RFP deletion constructs. Cells were fixed for immunofluorescence staining with RFP- (red) and cytochrome C- (green) specific antibodies. The nuclei were stained with Hoechst (blue). Images are representative of two independent experiments. Scale bar: 10 μm.

(**d**) Representative western blot analysis of cytosolic and mitochondrial sub-fractions obtained from HEK293T cells expressing the PDE2A2-RFP deletion constructs as indicated. An antibody for OXPHOS subunits was used as a control for mitochondrial protein loading. Blot is representative of two independent experiments.

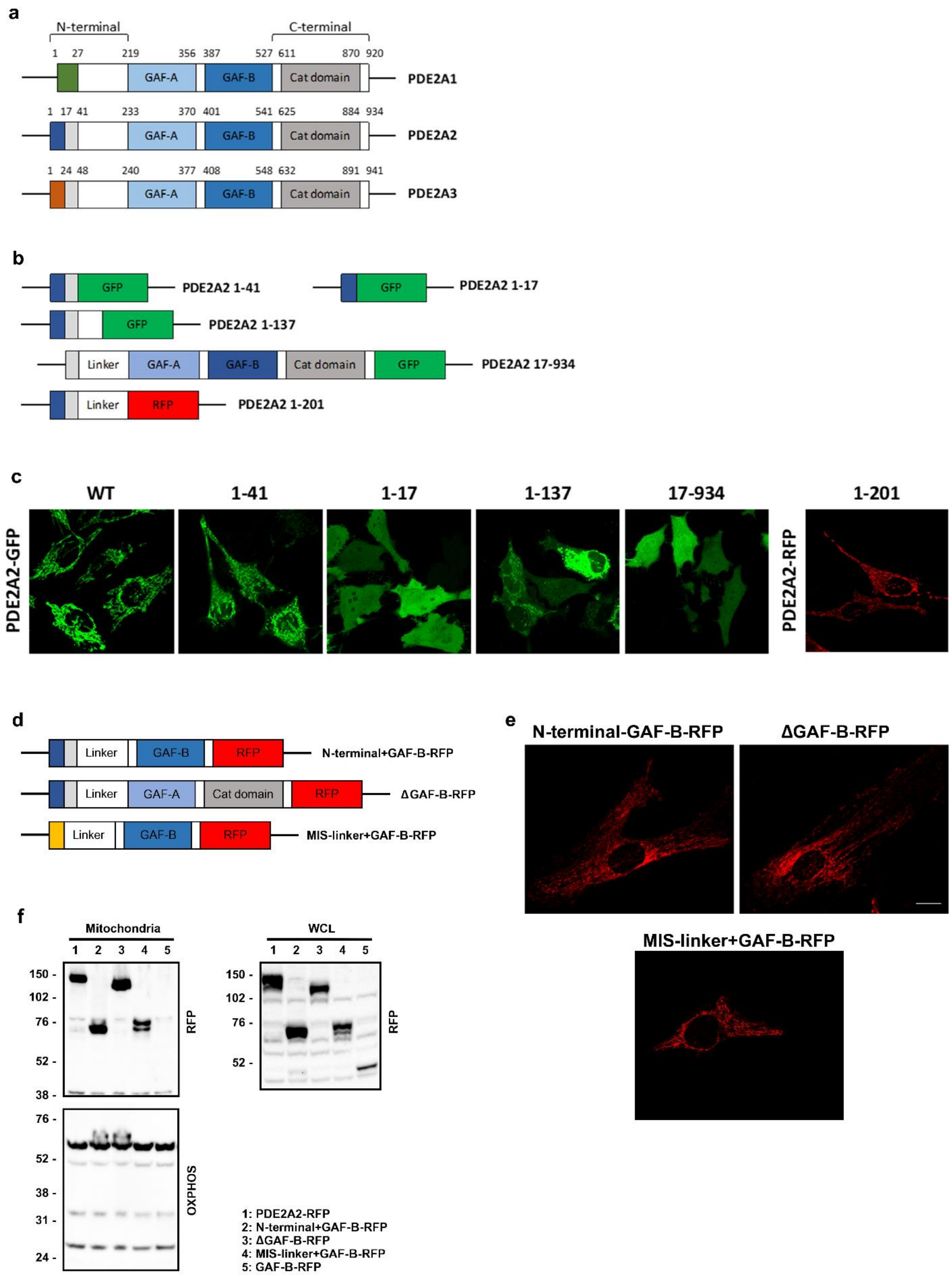
a
N-terminal
C-terminal
1 27 219 356 387 527 611 870 920
GAF-A
GAF-B
Cat domain
PDE2A1
1 17 41 233 370 401 541 625 884 934
PDE2A2
1 24 48 240 377 408 548 632 891 941
PDE2A3
b
GFP
PDE2A2 1-41
PDE2A2 1-17
PDE2A2 1-137
Linker
PDE2A2 17-934
RFP
PDE2A2 1-201
c
WT
1-41
1-17
1-137
17-934
1-201
PDE2A2-GFP
PDE2A2-RFP
d
N-terminal+GAF-B-RFP
ΔGAF-B-RFP
MIS-linker+GAF-B-RFP
e
N-terminal-GAF-B-RFP
ΔGAF-B-RFP
MIS-linker+GAF-B-RFP
f
Mitochondria
WCL
1 2 3 4 5
150 -
102 -
76 -
52 -
38 -
31 -
24 -
RFP
OXPHOS
1: PDE2A2-RFP
2: N-terminal+GAF-B-RFP
3: ΔGAF-B-RFP
4: MIS-linker+GAF-B-RFP
5: GAF-B-RFP

Supplementary Figure 3 | **Role of N-terminal domain of PDE2A2 in targeting the protein to the mitochondria.**

(**a**) Schematic representation of PDE2A isoforms domains. Different color at the N- terminal region of PDE2A1, PDE2A2 and PDE2A3 represents a different amino acid sequence. Numbering of amino acids refers to human PDE2A.

(**b**,**d**) Schematic of the PDE2A2 constructs generated. In **d**, the yellow box indicates amino acids 1-59 from the protein Smac/DIABLO (see min text)

Confocal images of HeLa (**c**) and MEF (**e**) cells expressing PDE2A2 constructs tagged with either GFP or RFP, as indicated. Although some localization to mitochondria is observed with truncations 1-41 and 1-137, only the 1-201 mutant shows full targeting to mitochondria. Scale bar 20 μm

(**f**) Detection by western blot of the localization of PDE2A2 or its modified versions, as indicated, in the whole cell lysate (WCL) or in the mitochondrial fraction of HEK293 cells.

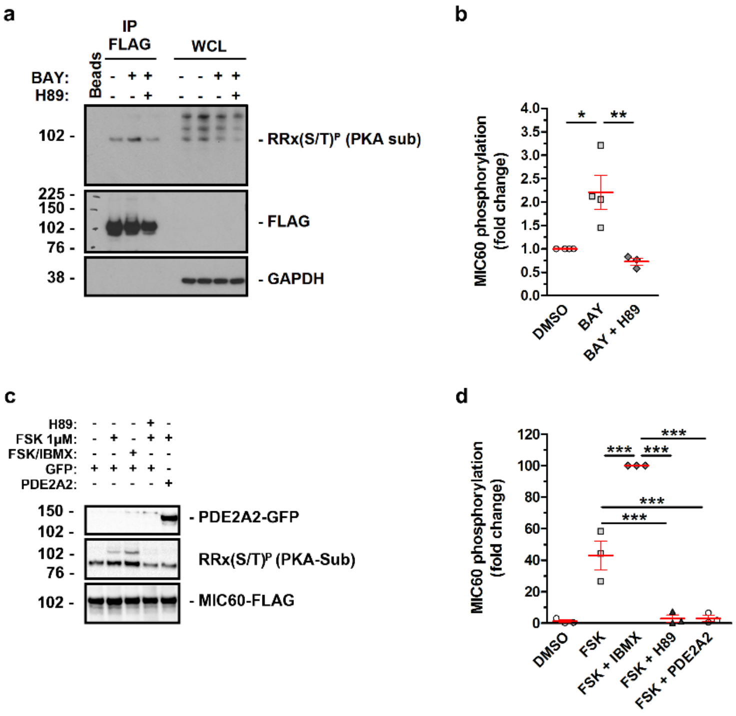

Supplementary Figure 4 | **Increased MIC60 phosphorylation on PDE2A inhibition depends on PKA.**

(**a**) Representative western blot and (**b**) quantification showing the phosphorylation status of MIC60 in HEK293T cells expressing MIC60-FLAG and treated with BAY (1 μM) alone or in combination with the PKA inhibitor H89 for 2h. (**c**) Representative western blot and (**d**) quantification showing the phosphorylation status of MIC60 in HEK293T cells expressing MIC60-FLAG in combination with GFP or PDE2A2-GFP and treated with foskolin (1 μM), H89 or IBMX (100 μM) as indicated. A phospho-(Ser/Thr) PKA substrate antibody was used both on whole cell lysate (WCL) or on MIC60-FLAG pull-down samples (IP). The amount of phosphorylated MIC60 was normalized to the amount of precipitated MIC60 and is shown as the fold change relative to levels in DMSO-treated cells (**b**) or relative to the phosphorylation achieved with forskolin and IBMX (**d**). N = 4 independent experiments. Data represents mean ± s.e.m. One-way ANOVA and Tukey's multiple comparison tests were performed. *P<0.05; ***P<0.005.

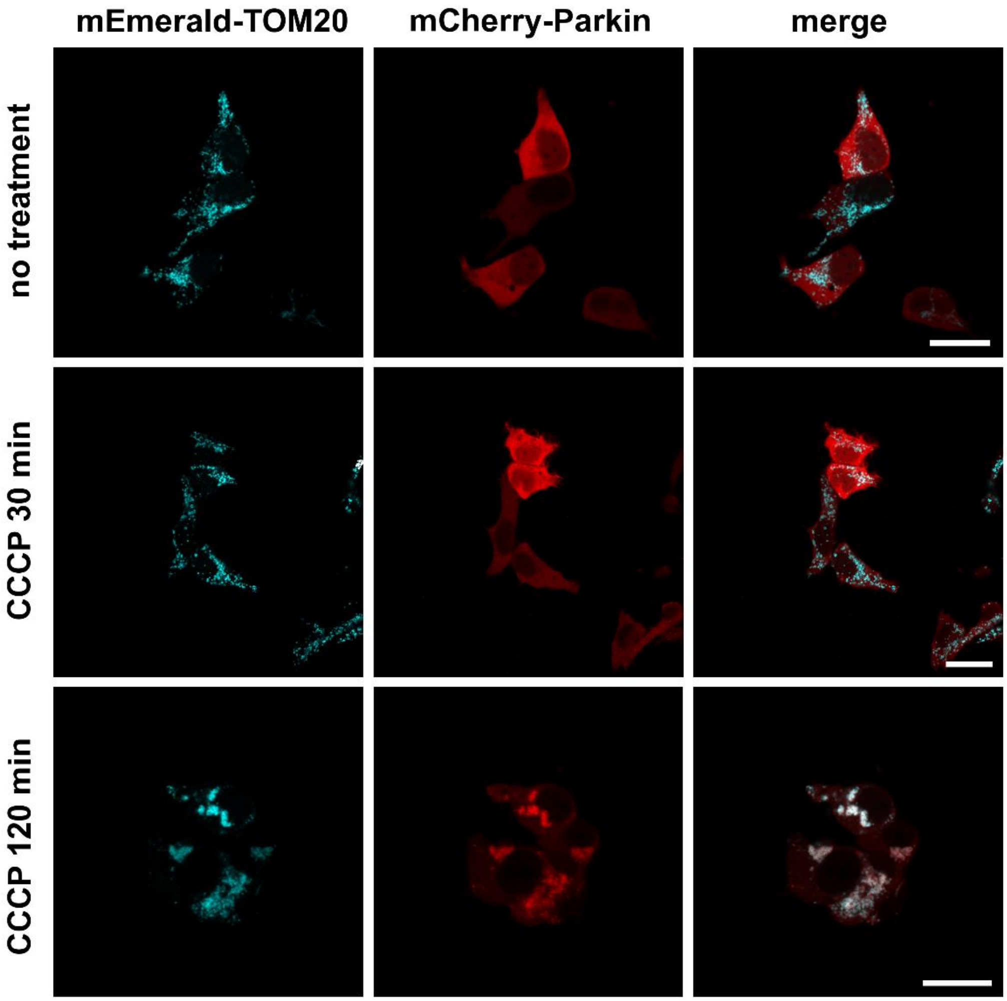

Supplementary Figure 5 | **Translocation of mCherry-Parkin to the mitochondria upon incubation with CCCP**. HEK293T cells expressing mCherry-Parkin and mEmerald-TOM20 (marker for mitochondria) under basal conditions, 30 min or 120 min incubation with 10 μM CCCP. Scale bar 20 μm

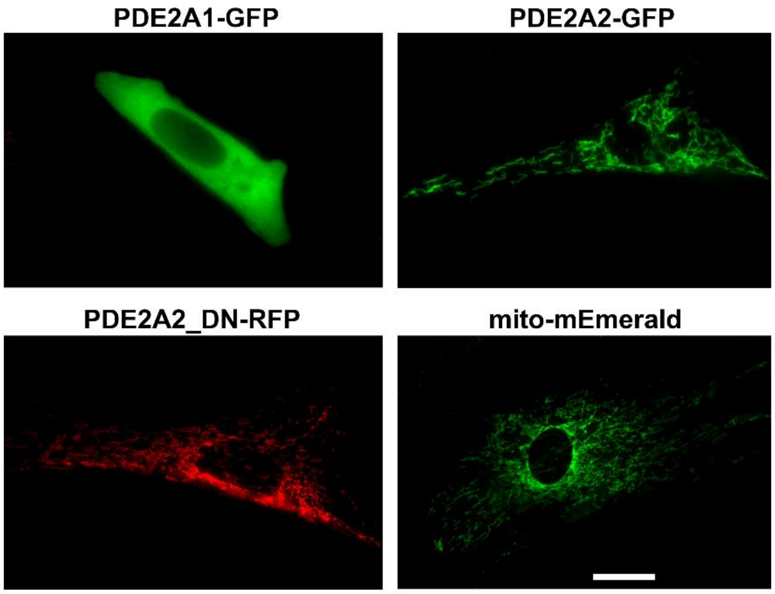

Supplementary Figure 6 | **Sub-cellular localization of PDE2A1, PDE2A2 and PDE2A2_DN in MEF cells**

MEF^{WT} cells expressing PDE2A1-GFP, PDE2A2-GFP, PDE2A2_DN-RFP or mito-mEmerald. Scale bar 20 μm

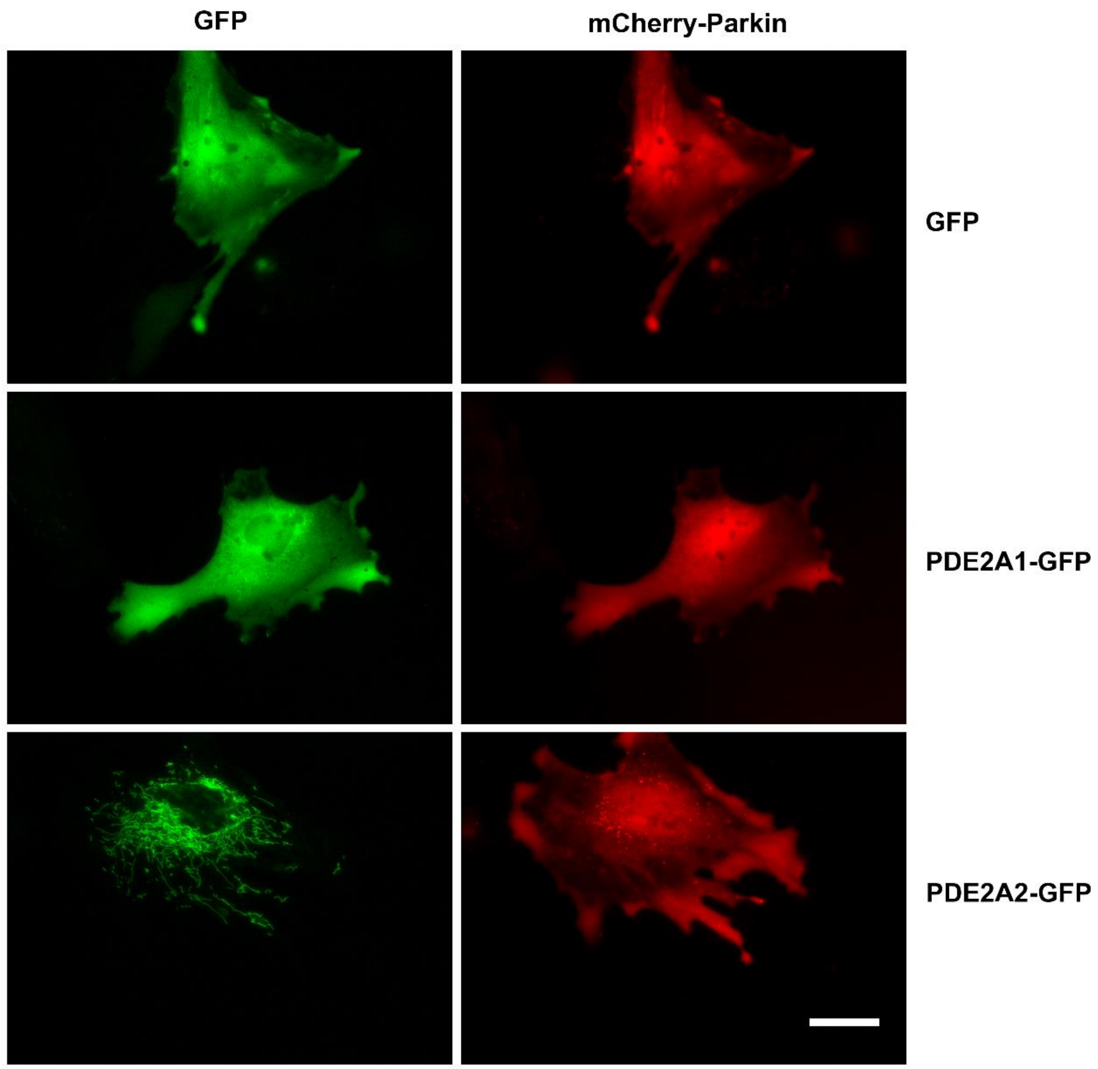

Supplementary Figure 7 | **Effect on Parkin recruitment of overexpressing PDE2A1 or PDE2A2**

Representative MEF^{WT} cells expressing Parkin-RFP in combination with PDE2A1-GFP, PDE2A2-GFP or GFP. Scale bar 20 μm

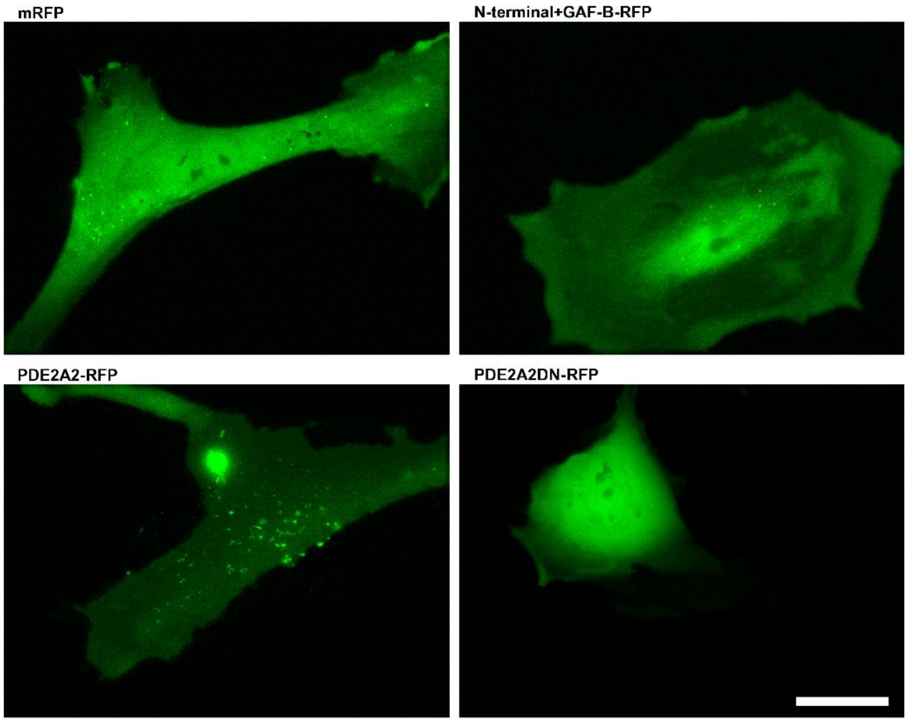

Supplementary Figure 8 | **Effect on Parkin recruitment of overexpressing mutant PDE2A2**
Representative MEF^{WT} cells expressing Parkin-GFP in combination with m-RFP, N-Term-GAB-B-RFP, PDE2A2-RFP or PDE2A2_DN-RFP. Scale bar 20 μm

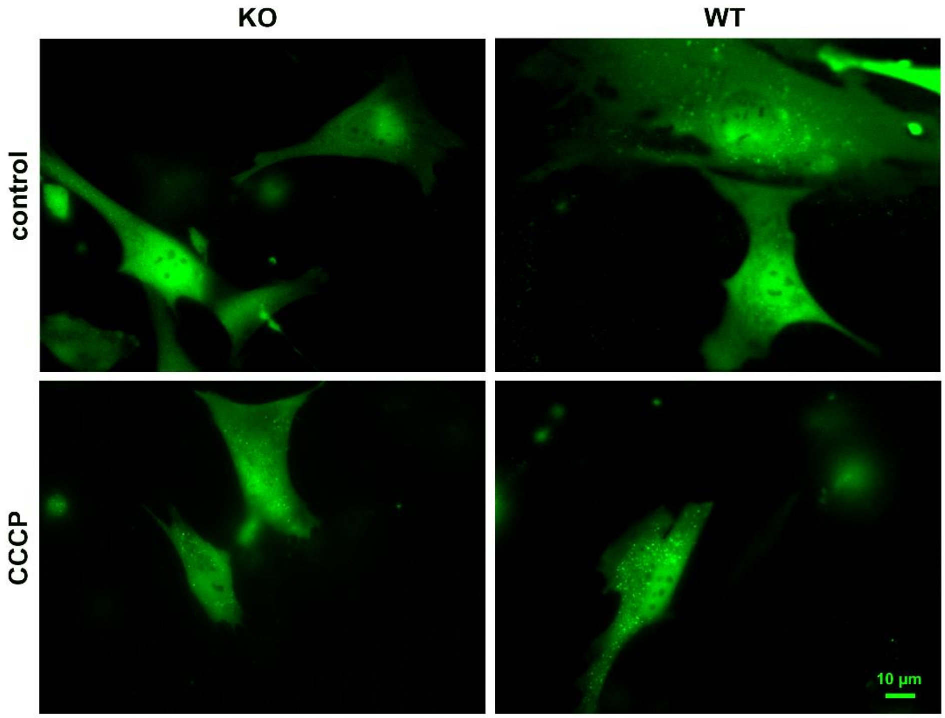

Supplementary Figure 9 | **Effect on Parkin recruitment of genetic ablation of *PDE2a***

Representative images of MEF^{WT} and $MEF^{PDE2-/-}$ expressing Parkin-GFP untreated or treated with 10µM CCCP for 120 min.

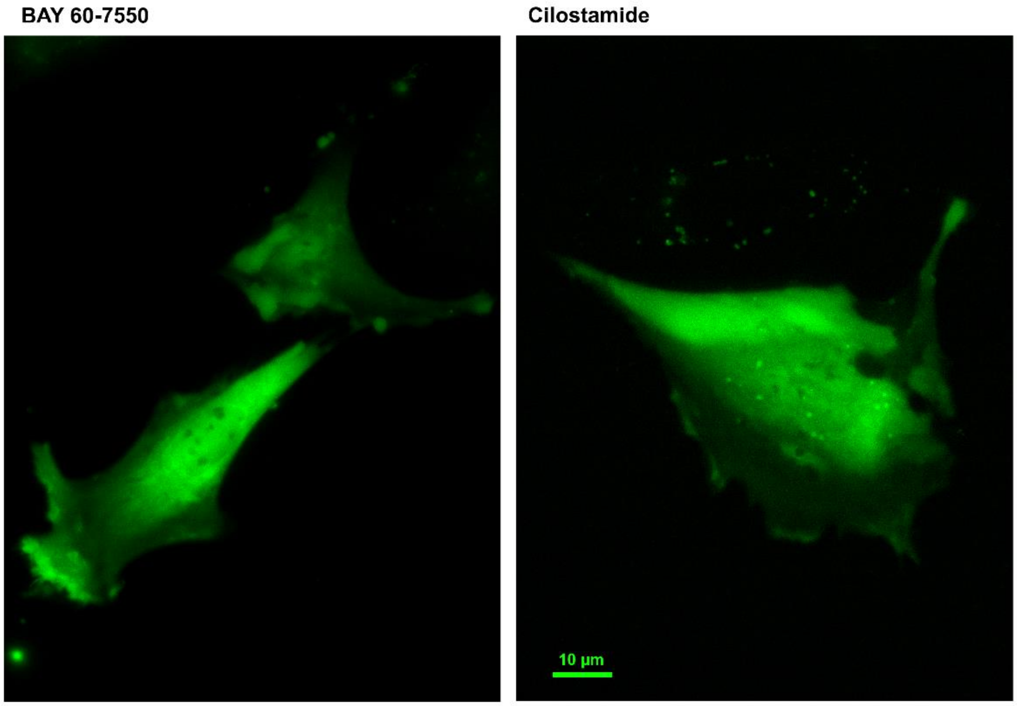

Supplementary Figure 10 | **Effect of PDE2A and PDE3 inhibition on Parkin recruitment** in MEF^{WT} treated with PDE2A or PDE3 inhibitors in the absence of CCCP.

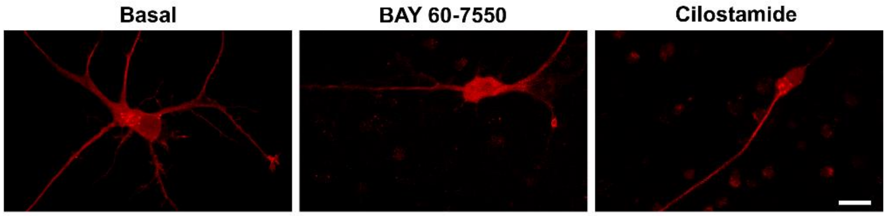

Supplementary Figure 11 | **Effect of PDE2A and PDE3 inhibition on Parkin recruitment** in inducible human pluripotent stem cell-derived dopaminergic neurons. Representative cells are shown. Scale bar 20 μm

Supplementary Table 1

List of primers used to amplify target genes

Cox7a forward	CAGCGTCATGGTCAGTCTGT
Cox7a reverse	AGAAAACCGTGTGGCAGAGA
Cox8b forward	GAACCATGAAGCCAACGACT
Cox8b reverse	GCGAAGTTCACAGTGGTTCC
Tfam forward	CCTTCGATTTTCCACAGAACA
Tfam reverse	GCTCACAGCTTCTTTGTATGCTT
Elovl3 forward	TCCGCGTTCTCATGTAGGTCT
Elovl3 reverse	GGACCTGATGCAACCCTATGA
Ppargc1a forward	GCACACACCGCAATTCTCCCTTGTA
Ppargc1a reverse	ACGCTGTCCCATGAGGTATTGACCA
Ucp1 forward	GGTGAACCCGACAACTTCCGAAGTG
Ucp1 reverse	GGGTCGTCCCTTTCCAAAGTGTTGA
Hprt forward	GTCCCAGCGTCGTGATTAGC
Hprt reverse	TCATGACATCTCGAGCAAGTCTTT
mt-Atp6 forward	GCATTAGCAGTCCGGCTTAC
mt-Atp6 reverse	GGTAGCTGTTGGTGGGCTAA
mt-Atp8 f forward	GGCACCTTCACCAAAATCACT
mt-Atp8 reverse	TGGGGTAATGAATGAGGCAAAT
mt-Co1 forward	TACTATTCGGAGCCTGAGCG
mt-Co1 reverse	AGTGCACCTGGTTGACCTAA
mt-Co2 forward	CTGAAGACGTCCTCCACTCA
mt-Co2 reverse	TGATTTAGTCGGCCTGGGAT

Original Western-Blot Scans - Main Article

Original scans Figure 1

Figure 1c MIC60:

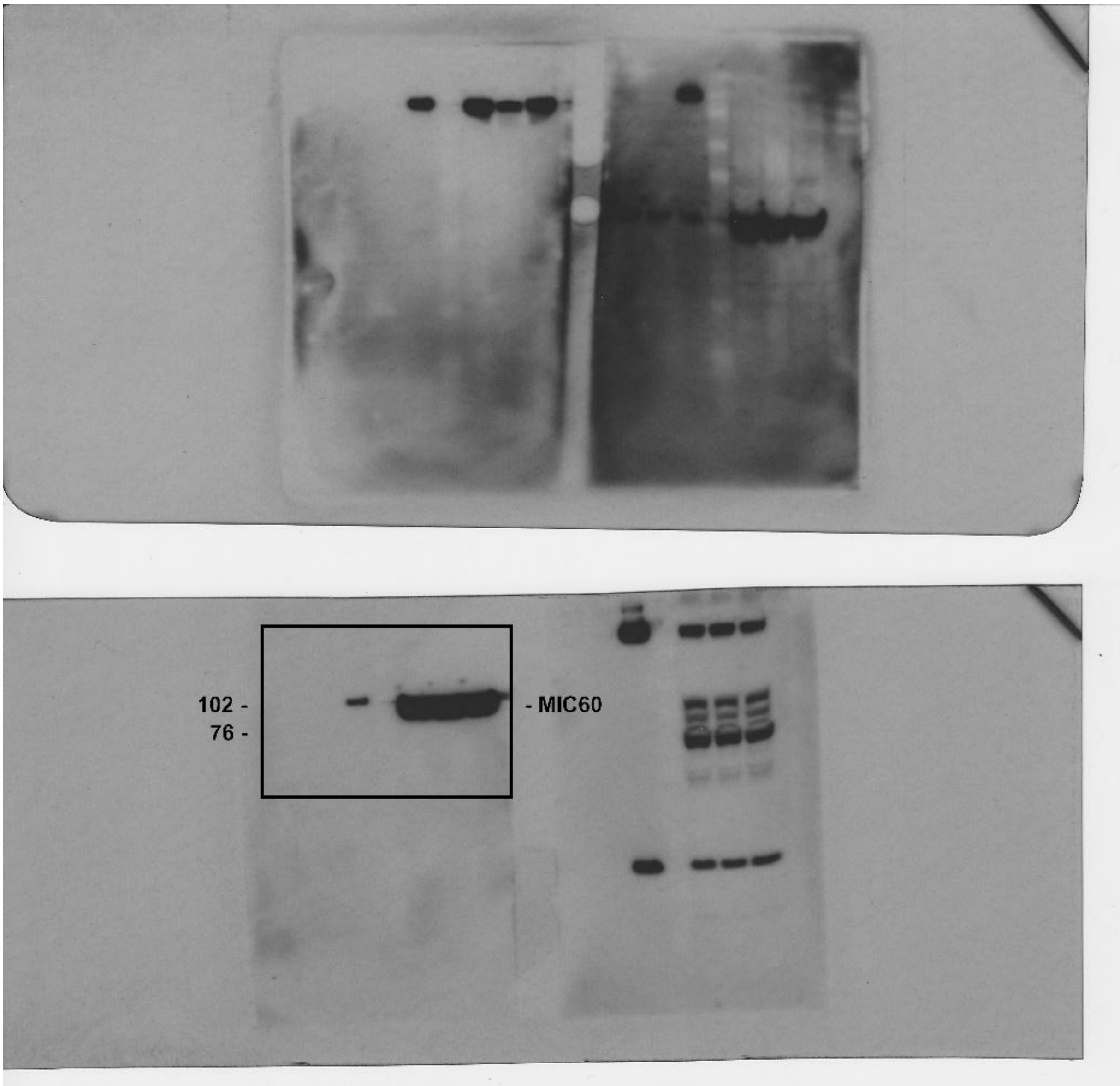

Figure 1 c lower panel (PDE2A-RFP, GAPDH and mRFP):

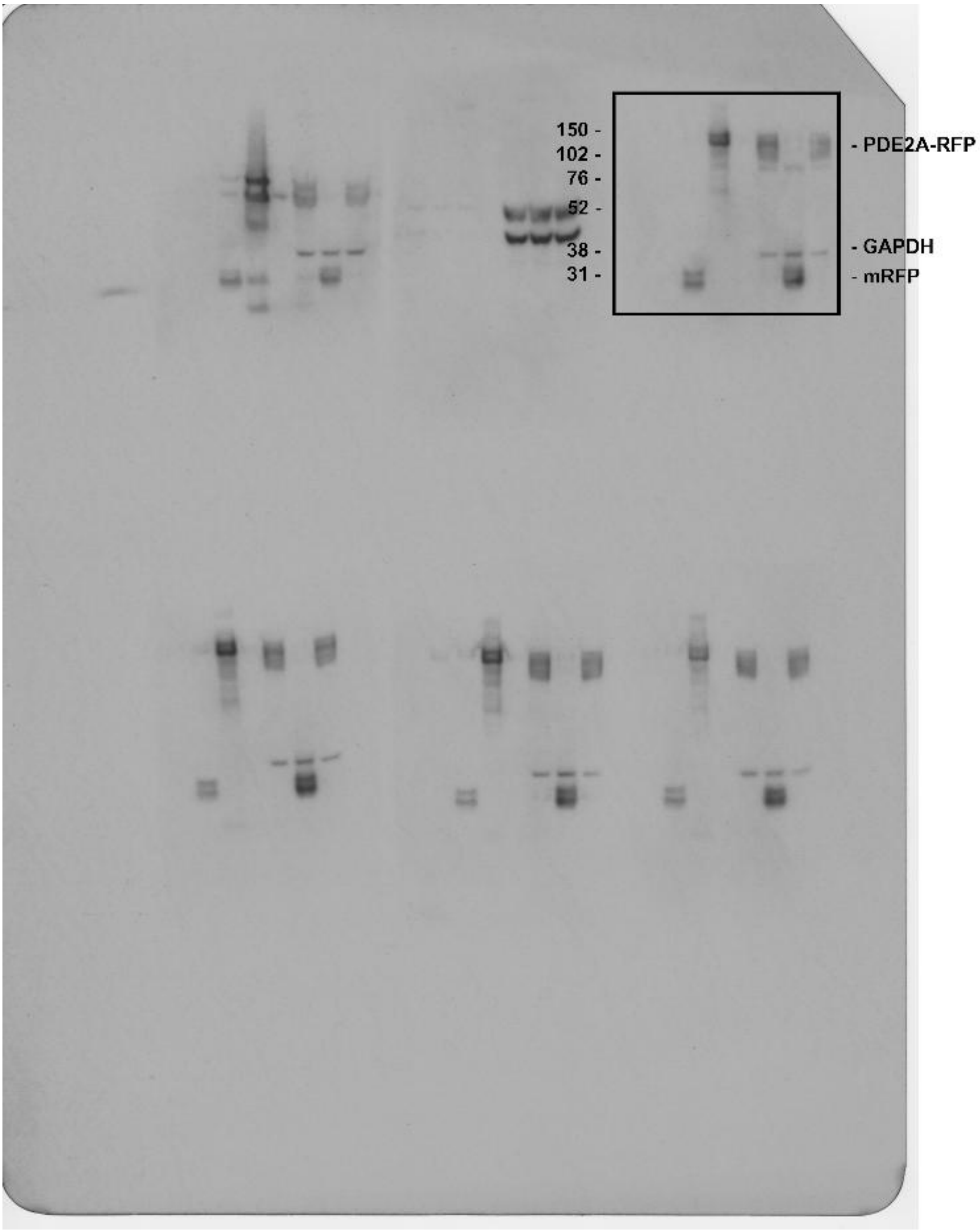

Figure 1 d and e upper panel (MIC19 and SAMM50):

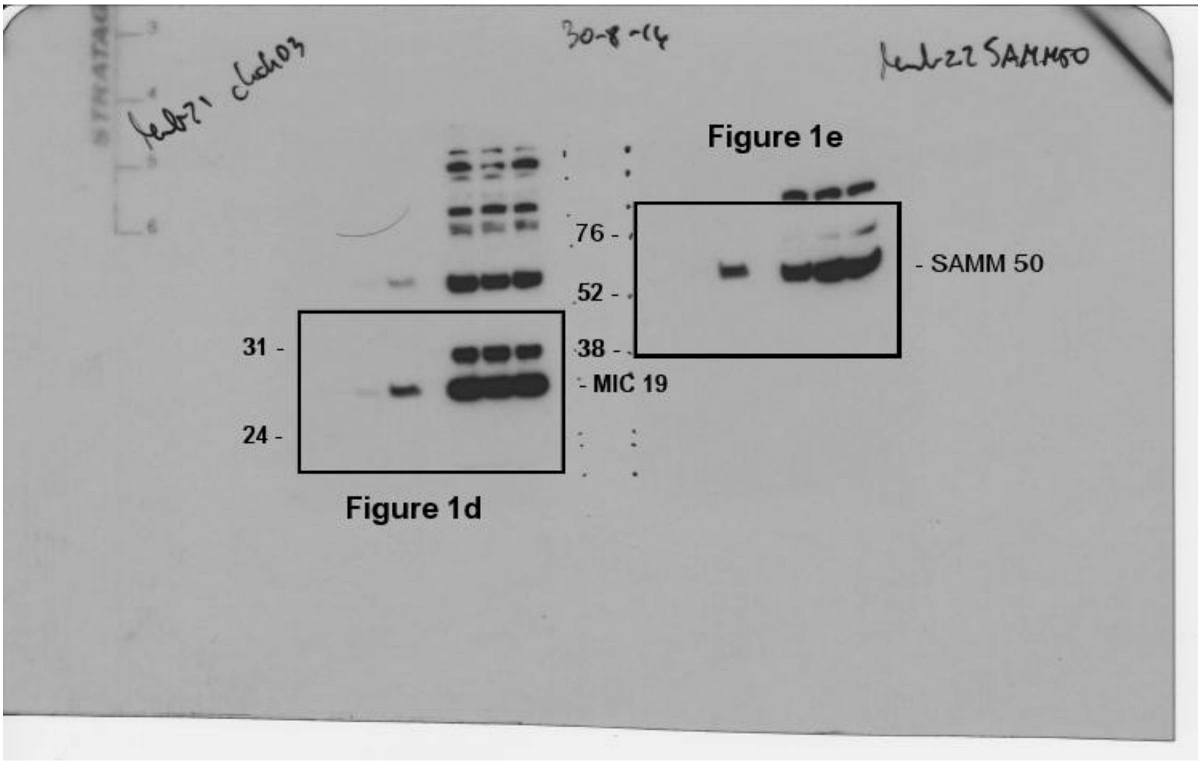

Figure 1d panel RFP:

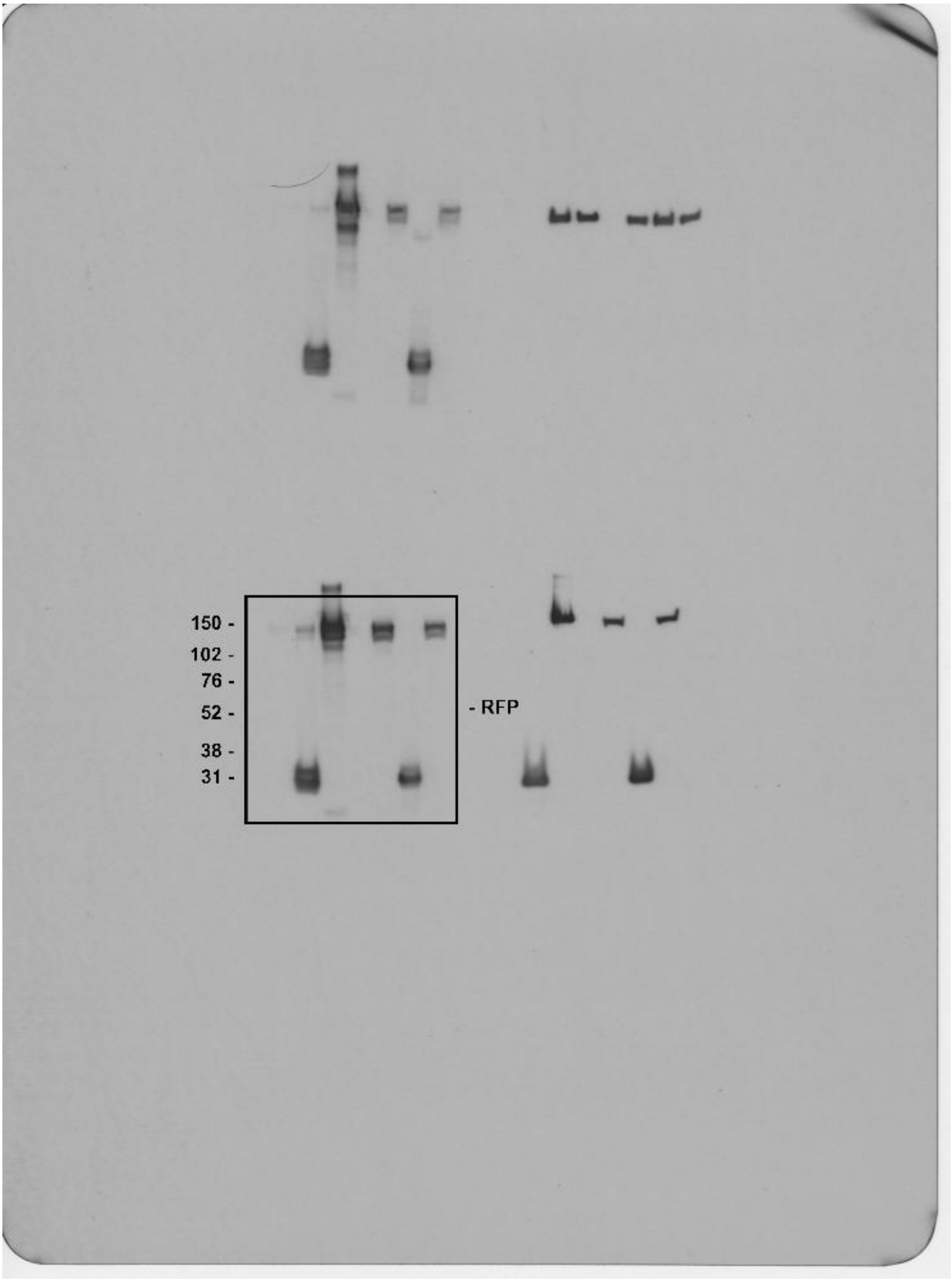

Figure 1 d panel GAPDH:

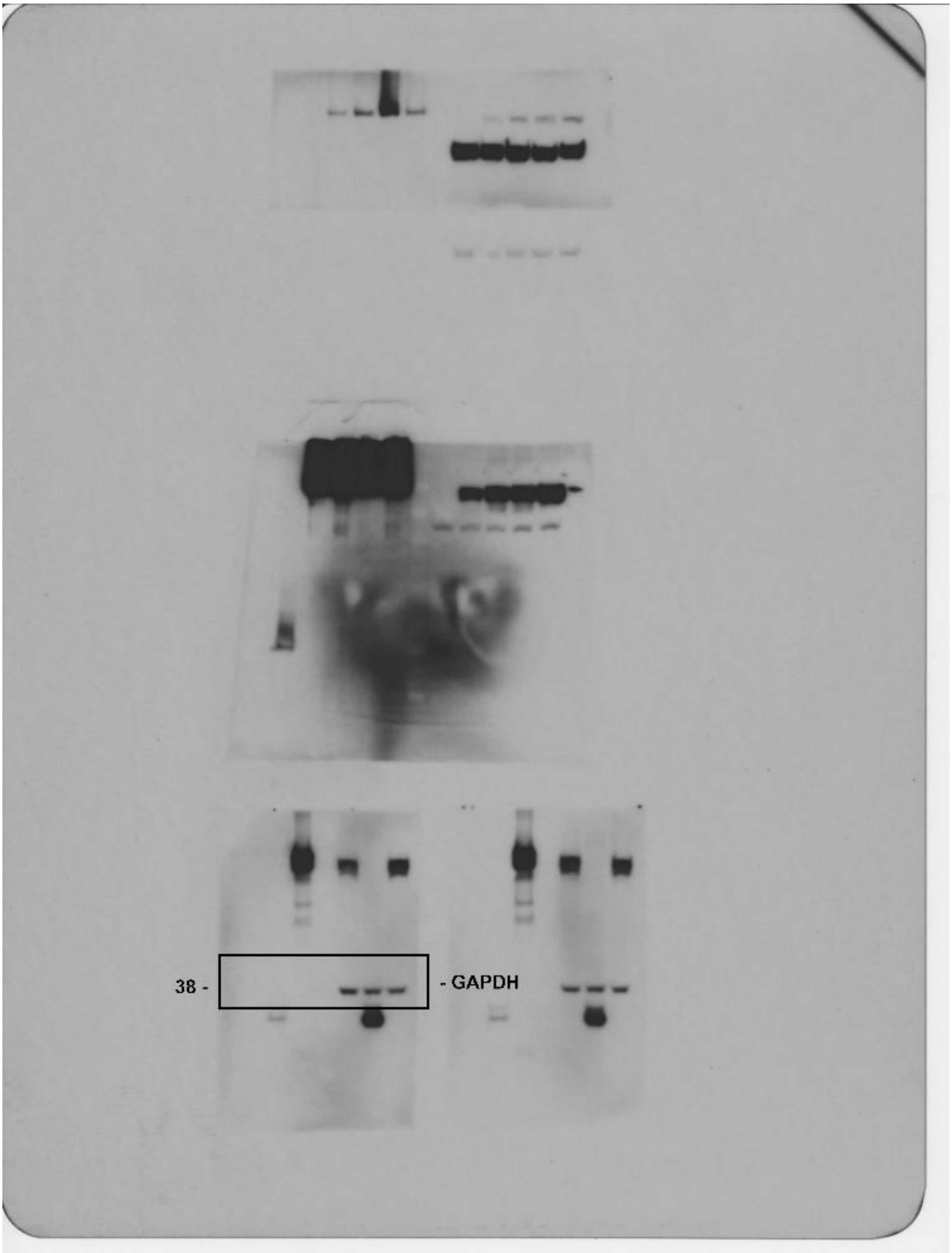

Figure 1 f panel PDE2A:

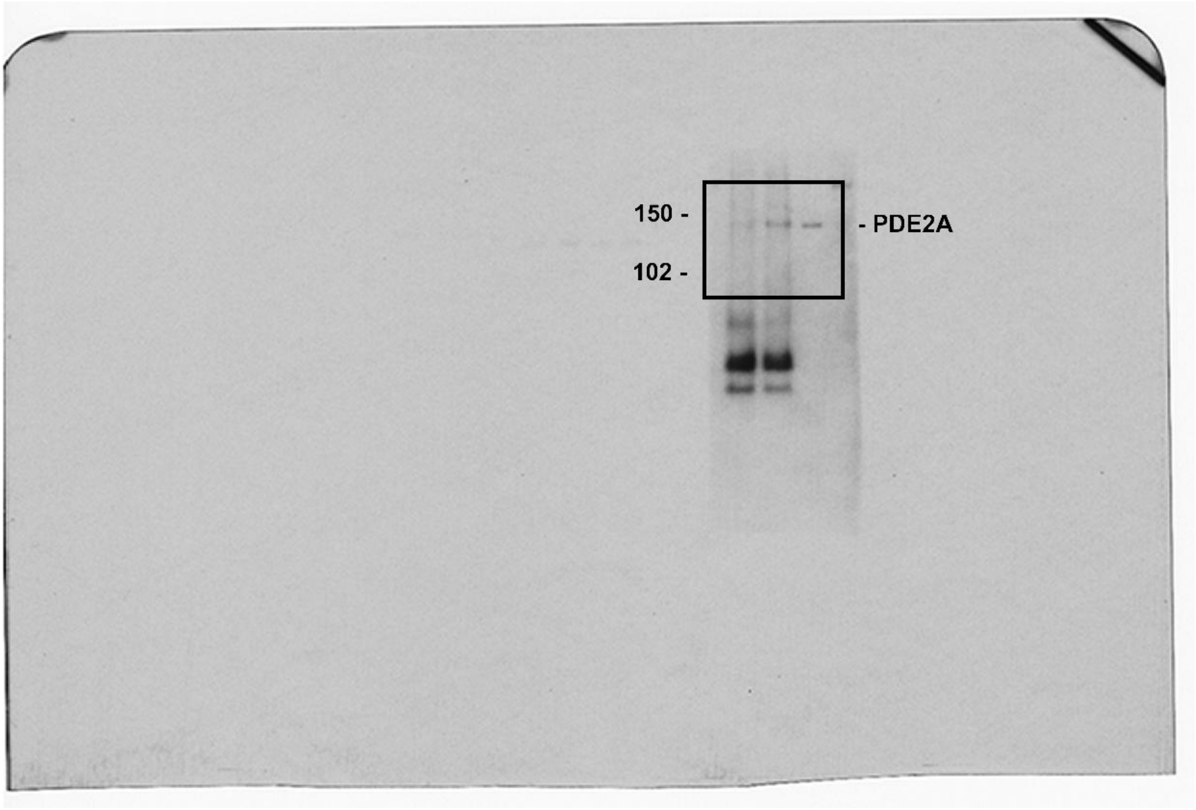

Figure 1 e panel MIC60:

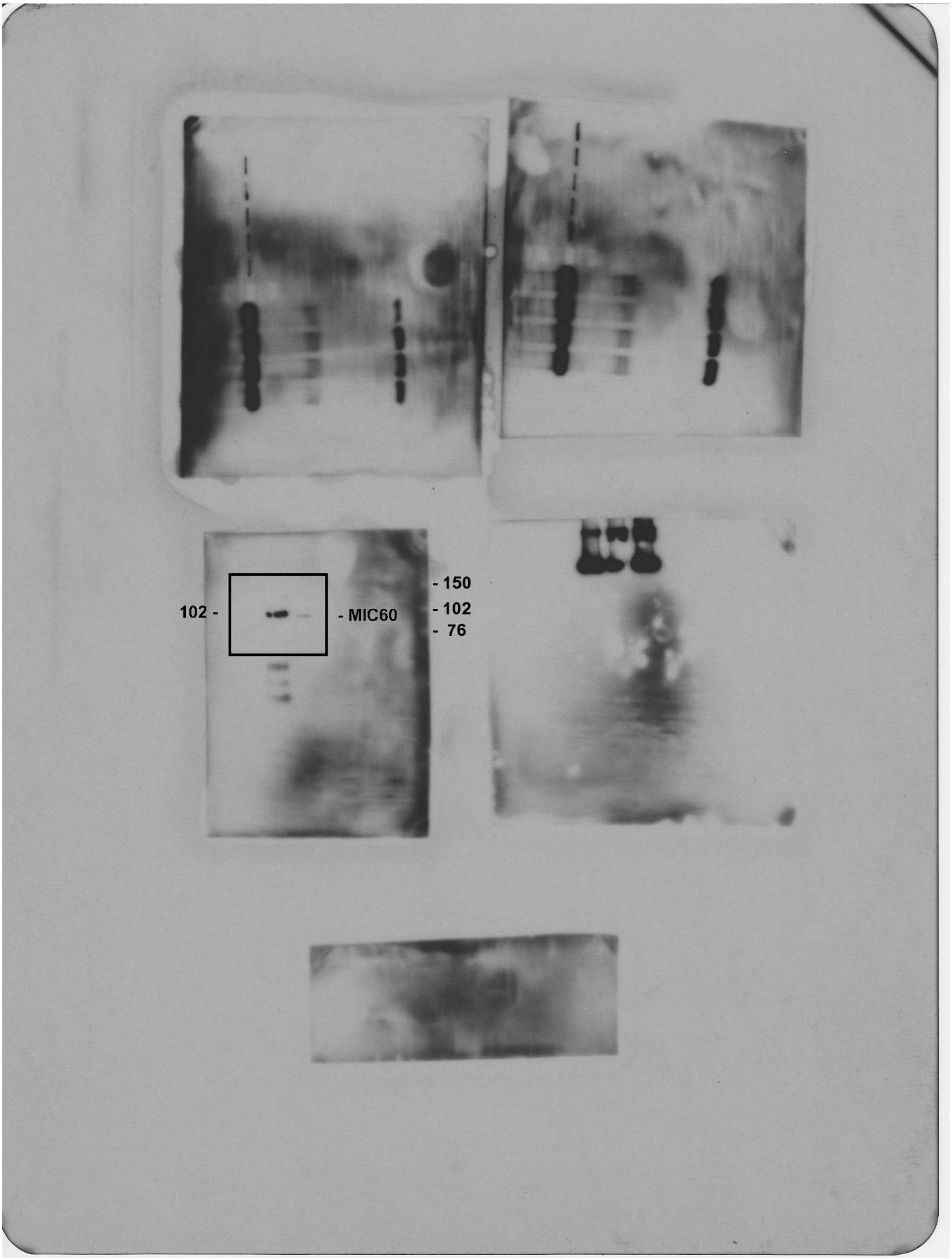

Figure 1 f panel alpha-Tubulin and GAPDH:

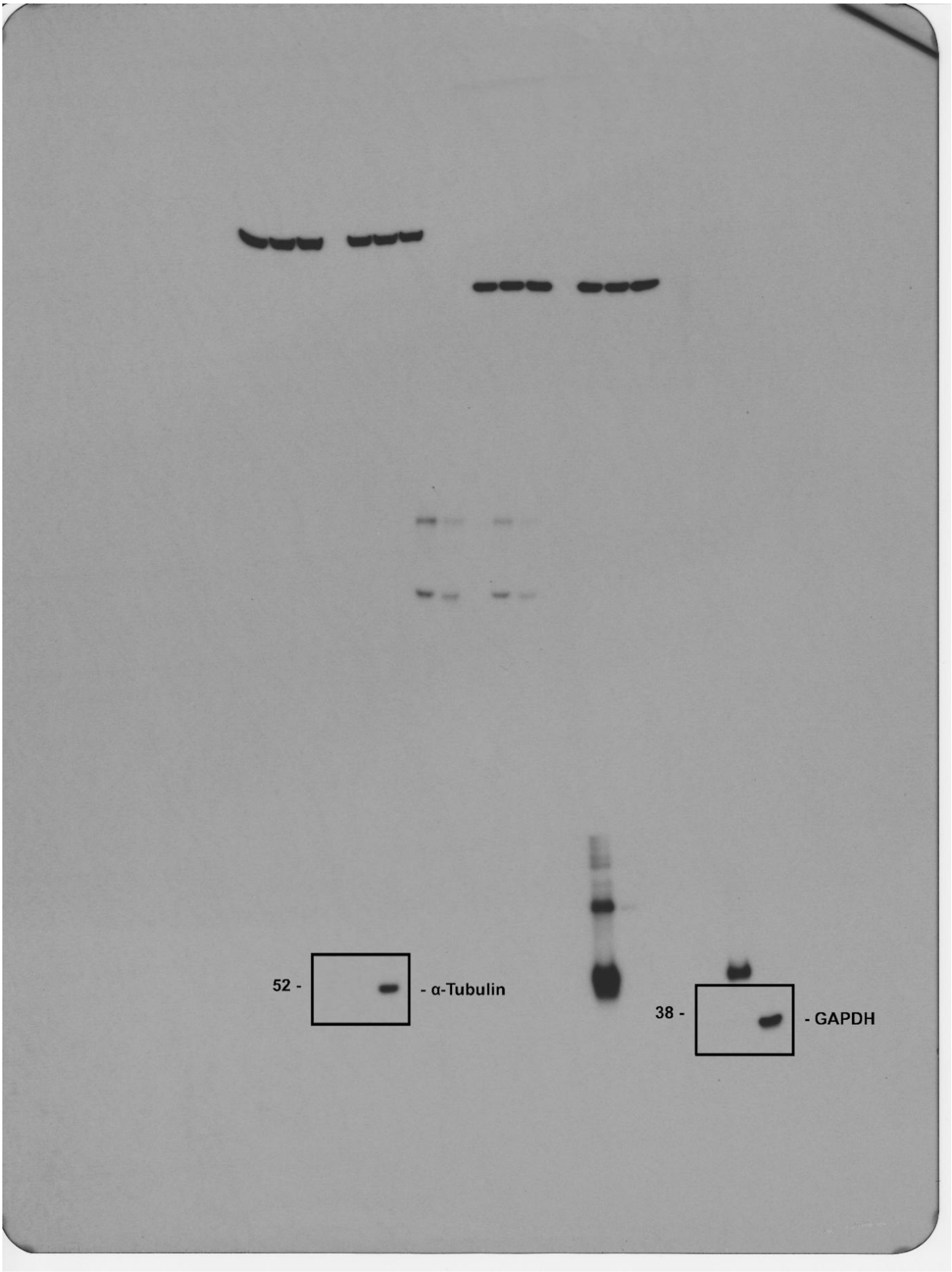

Figure 1 g panel MIC60:

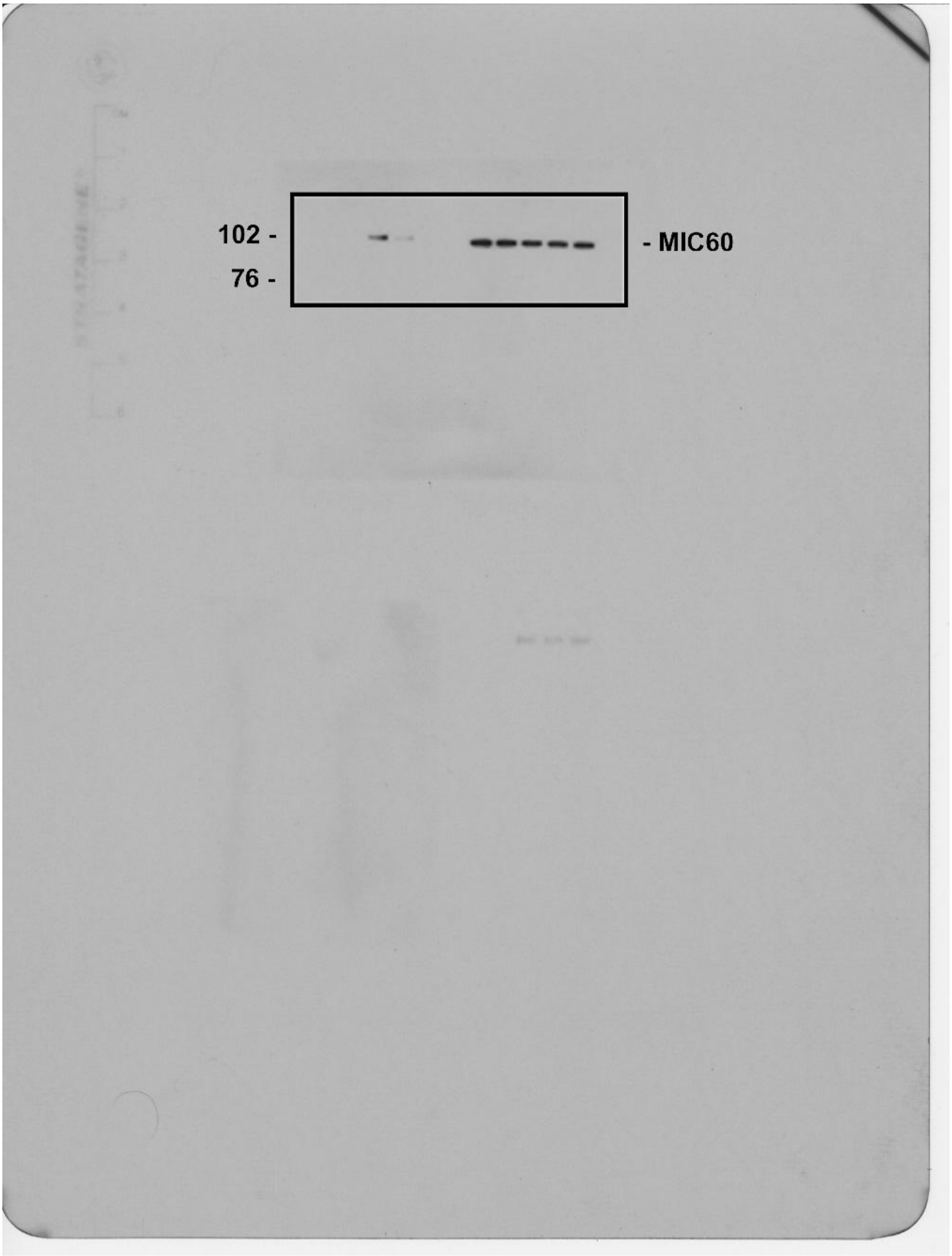

Figure 1 g panel PDE2A-GFP and GAPDH:

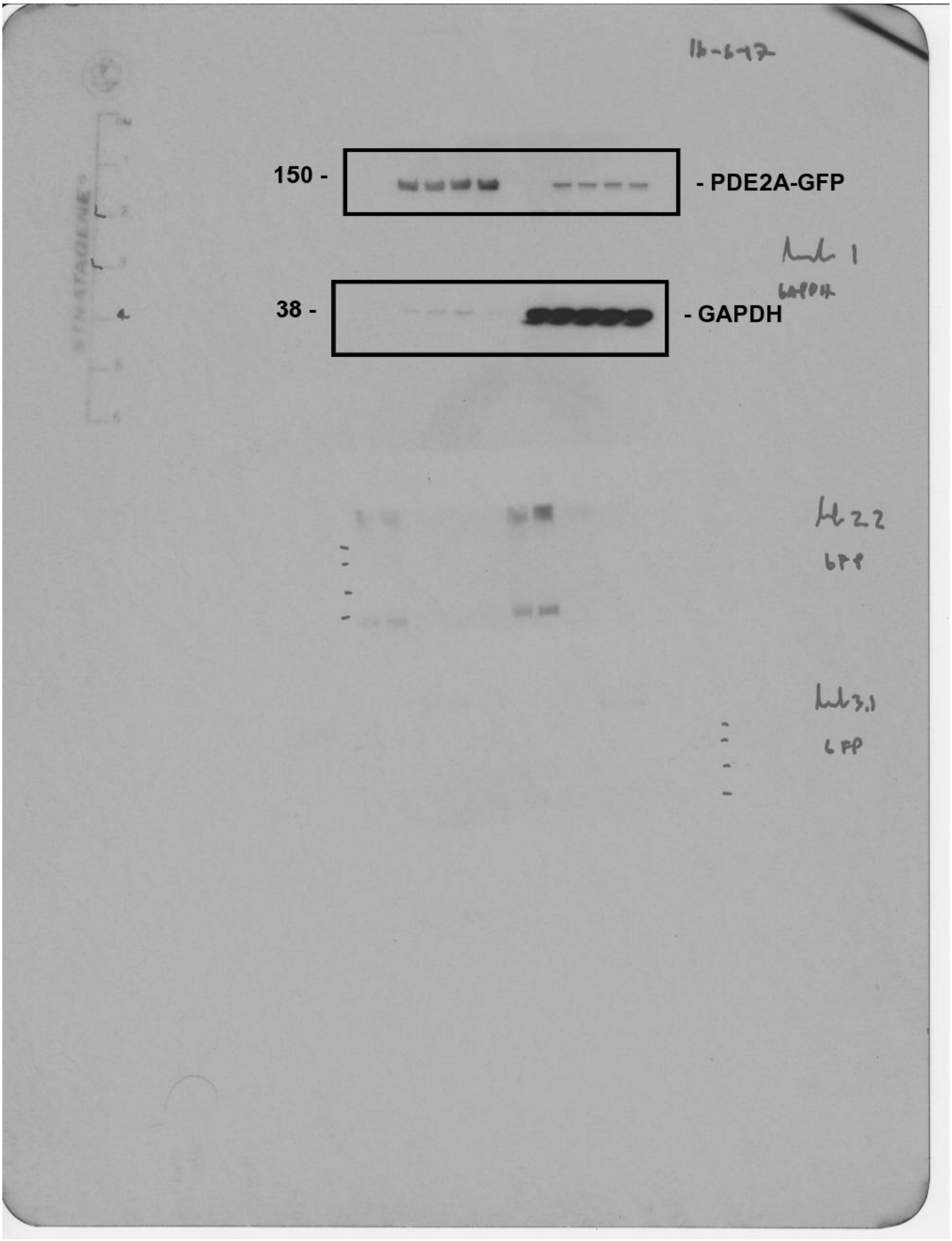

Figure 1 g panel PDE2A-GFP and GAPDH shorter exposition:

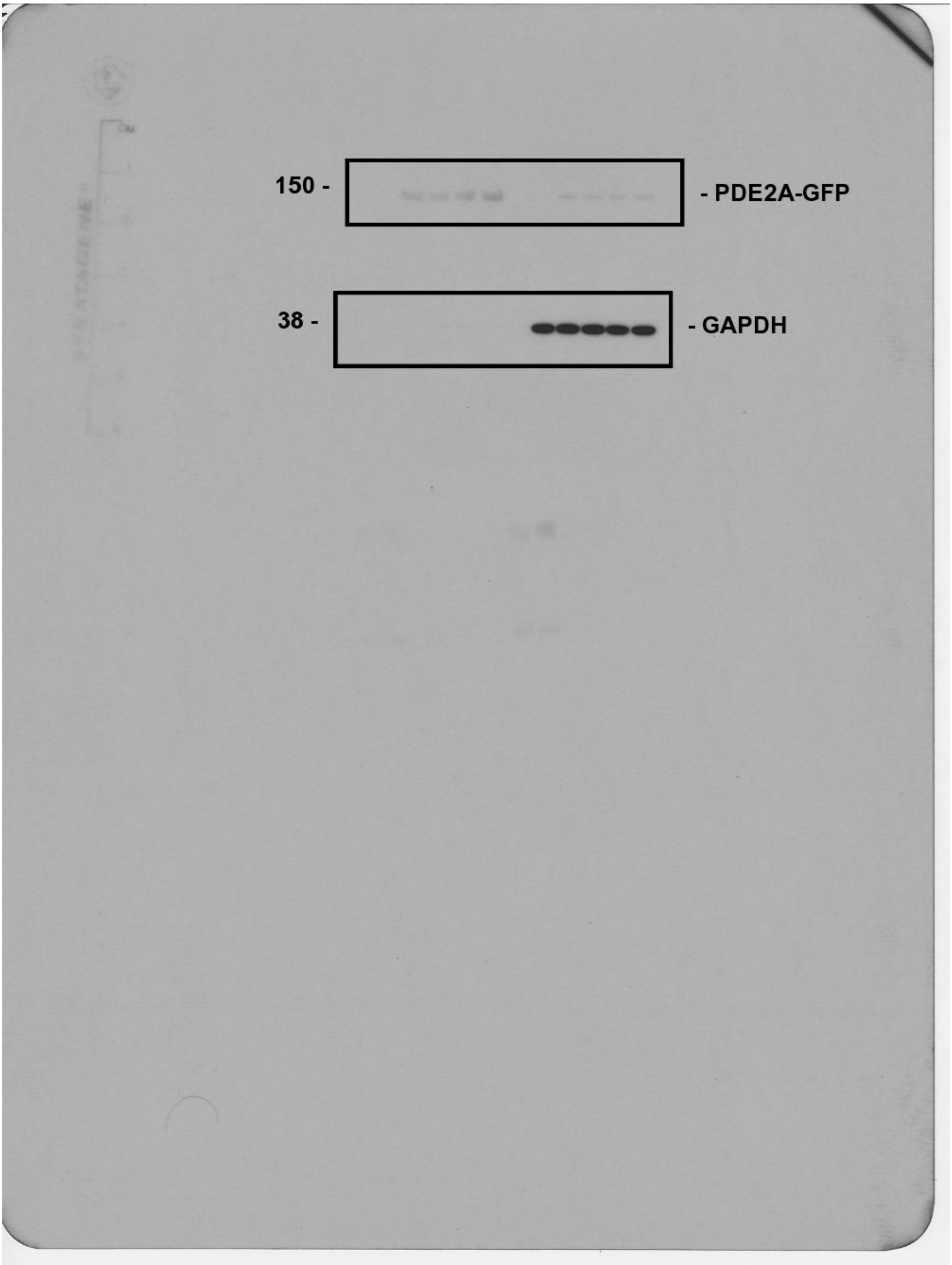

Figure 1 g Panel GFP:

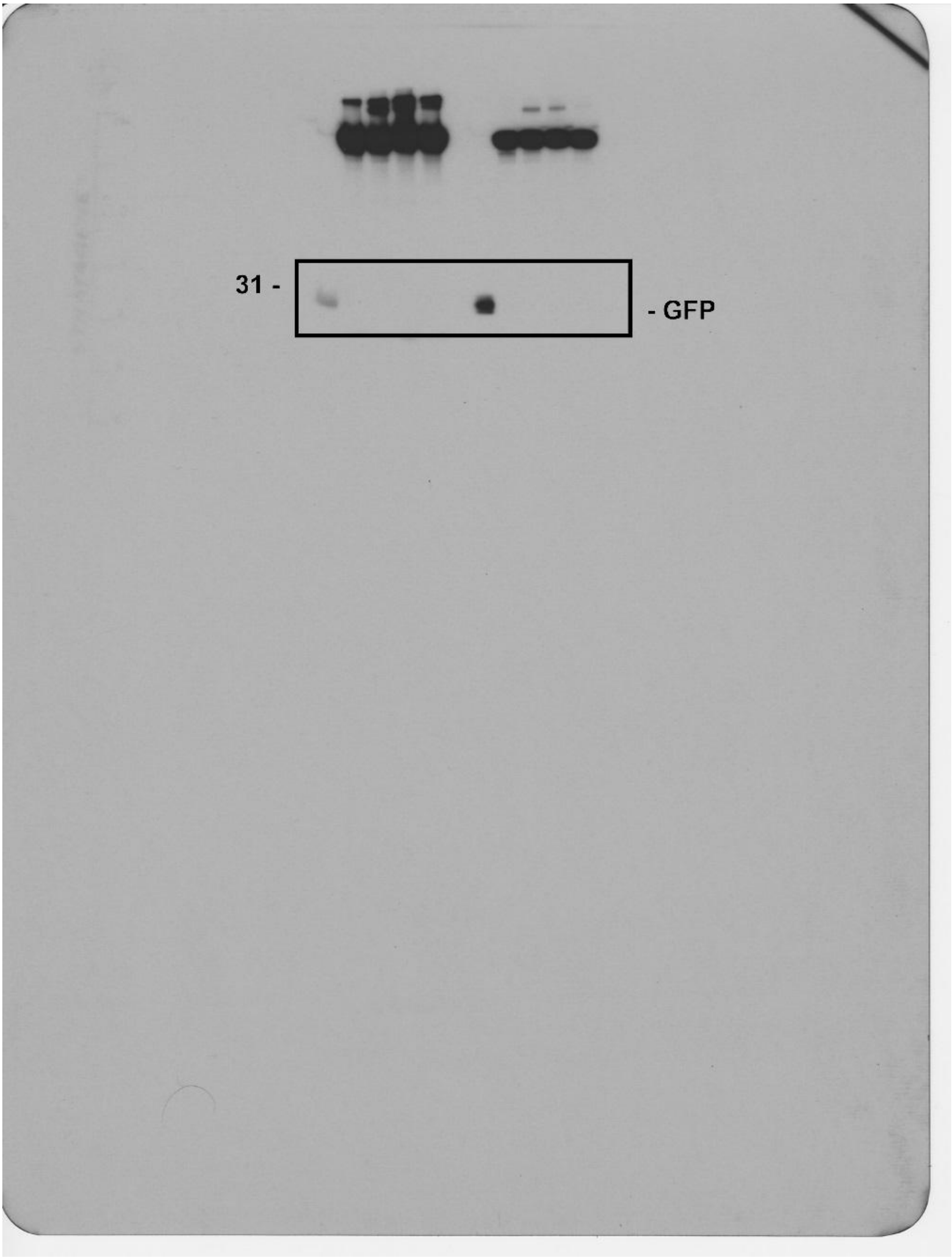

Original scans Figure 2

Figure 1a MIC60 IP (left Panel):

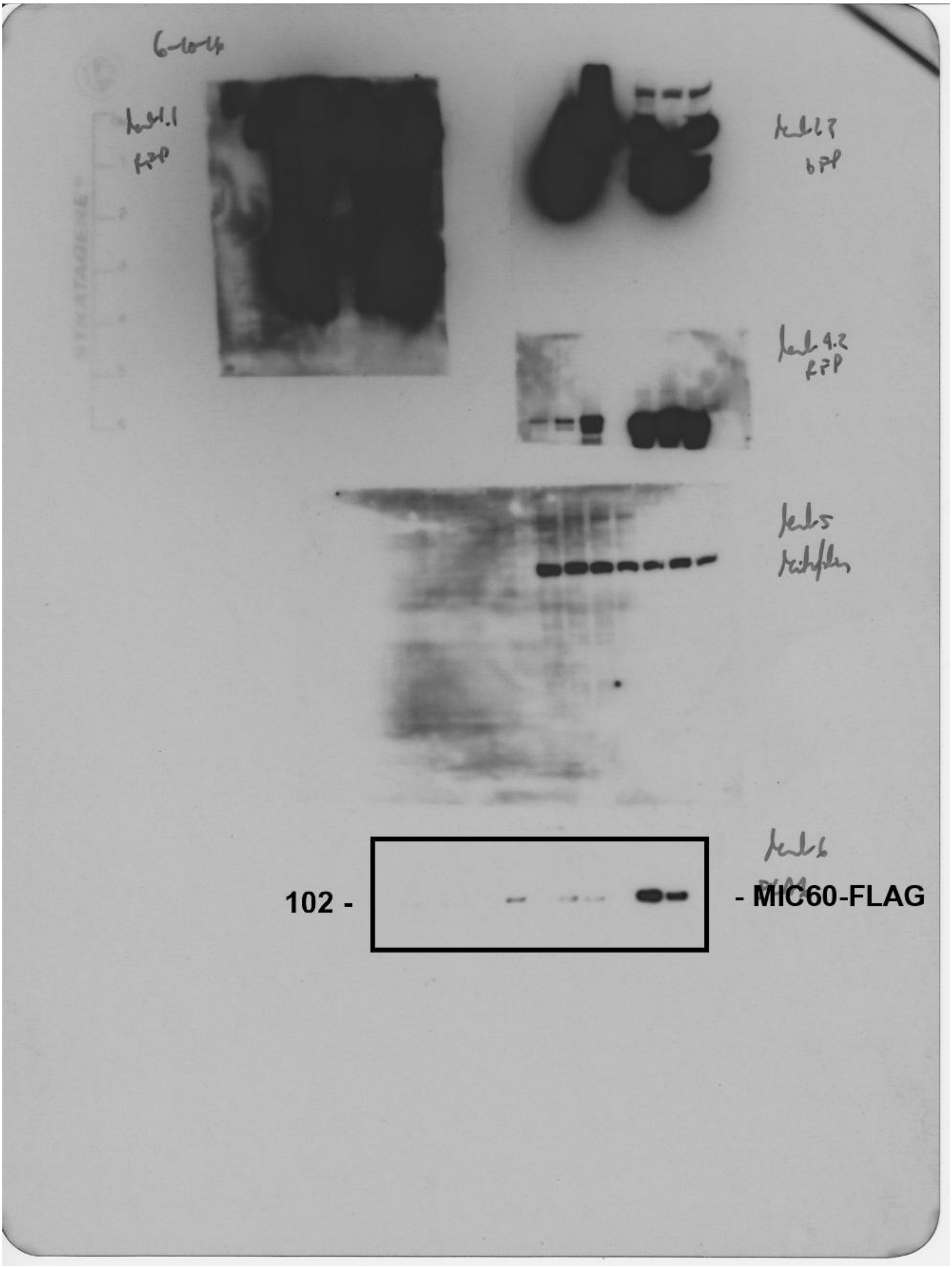

Figure 1a MIC60 WCL (right Panel):

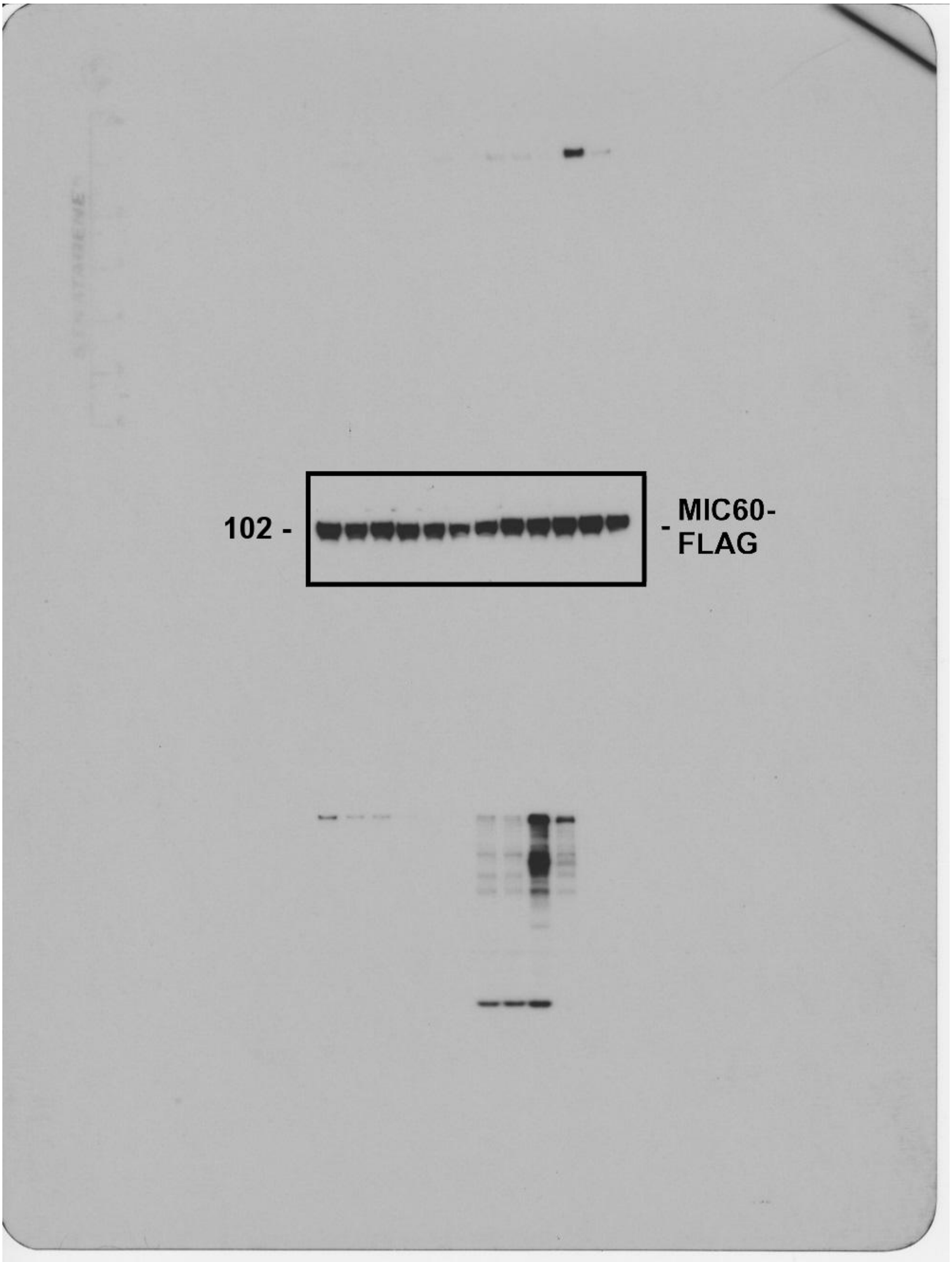

Figure 2A middle panels RFP:

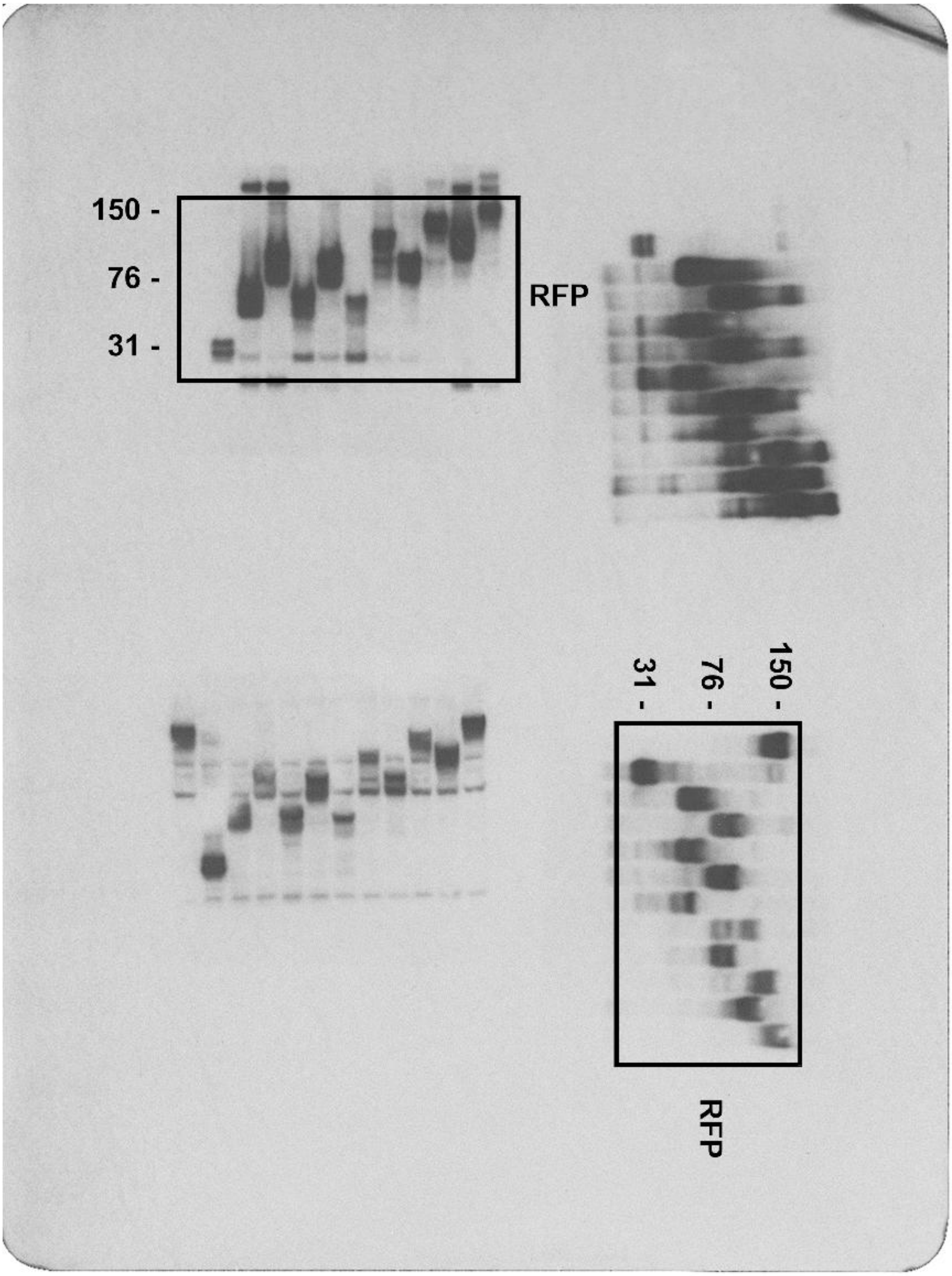

Figure 2A alpha-Tubulin:

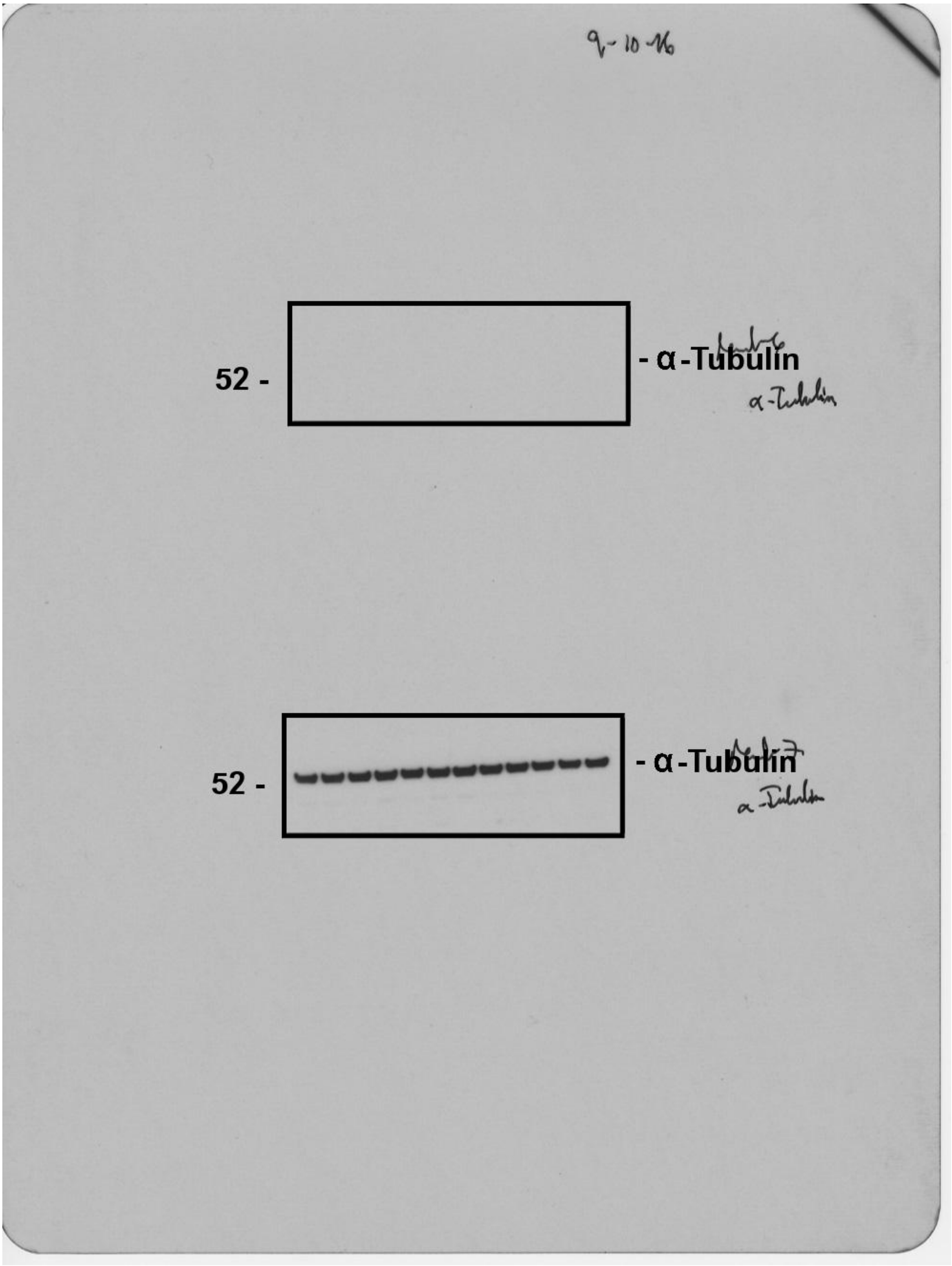

Figure 2B MIC60 FLAG:

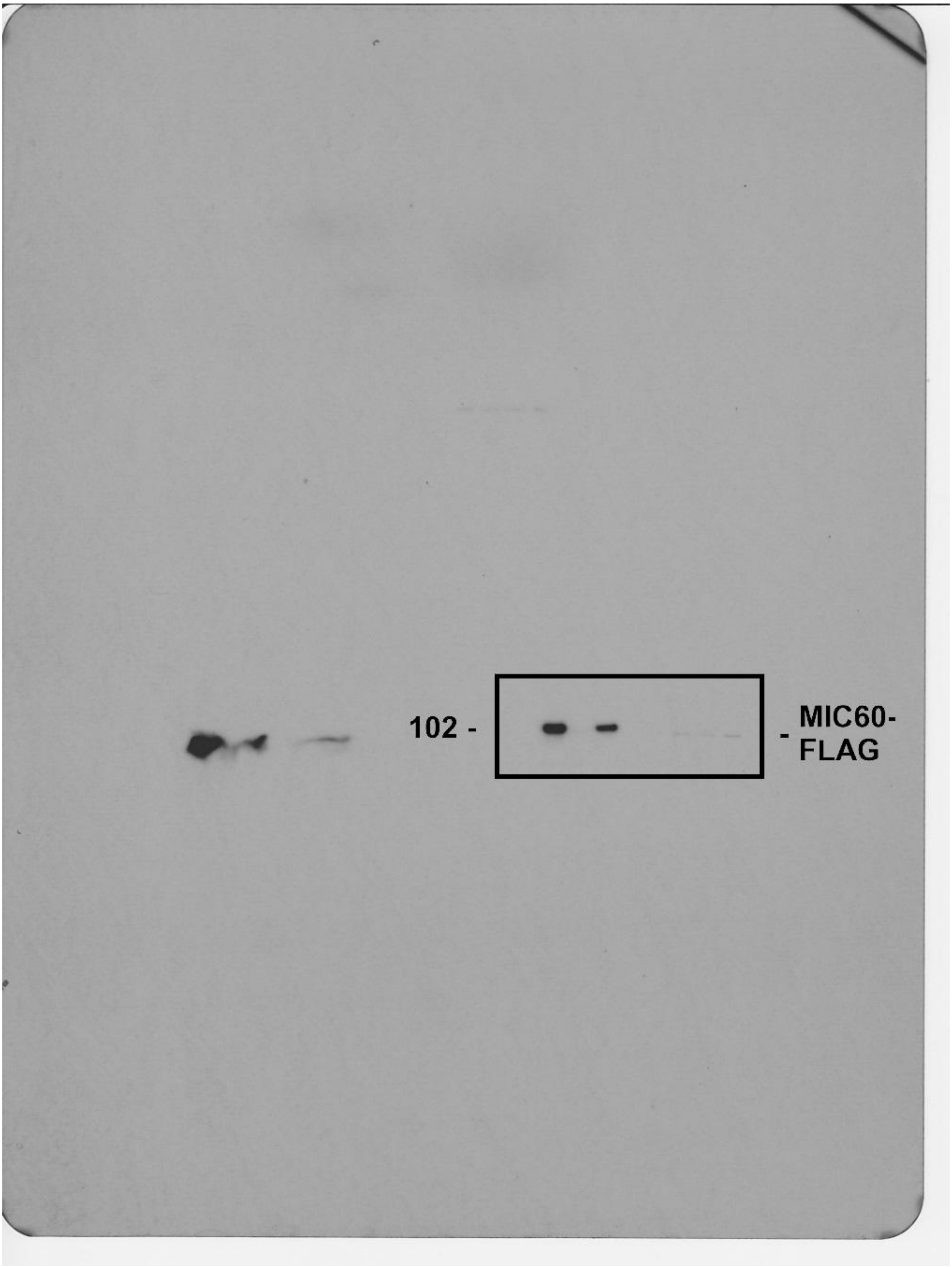

Figure 2b RFP:

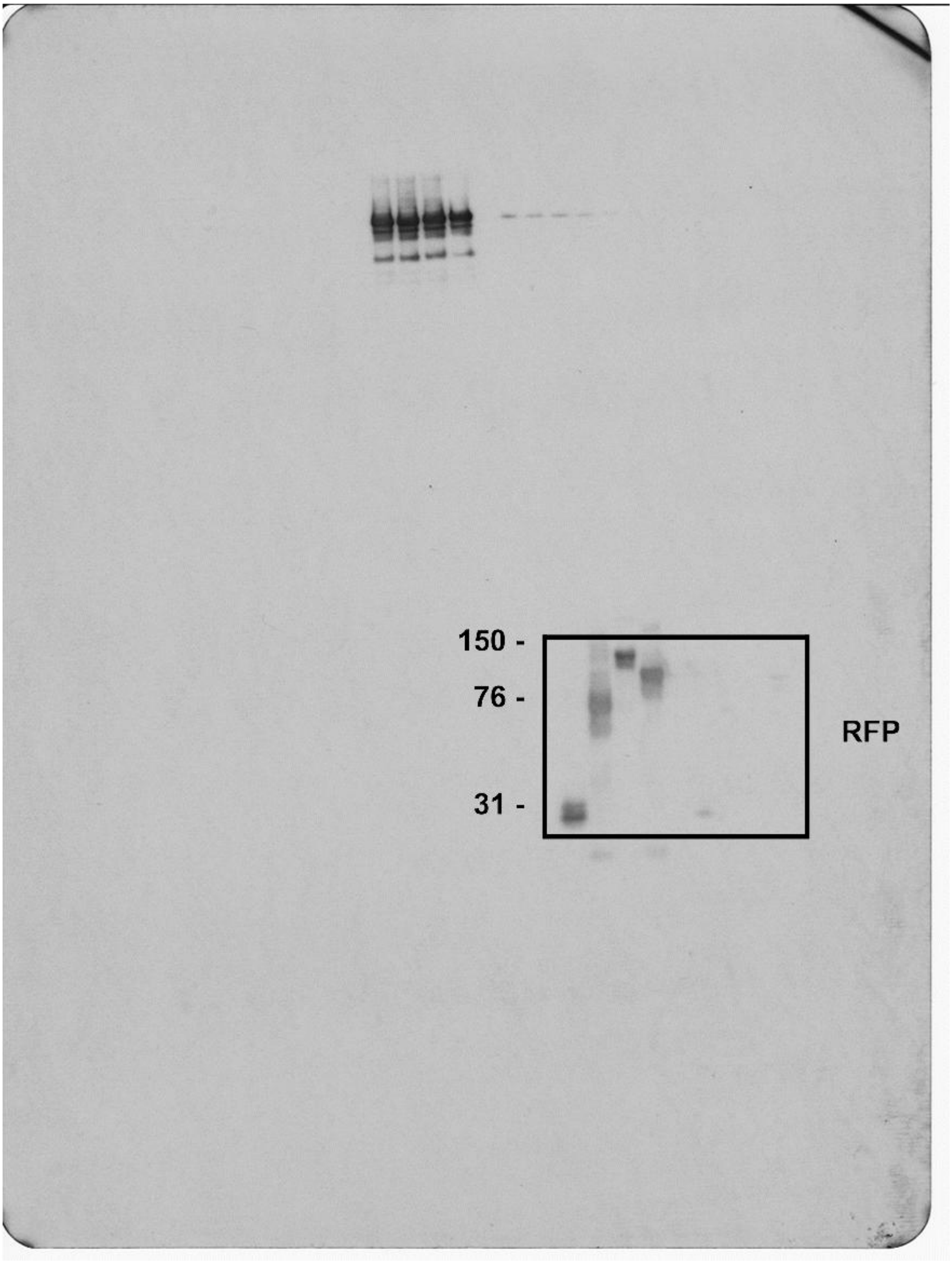

Figure 2B GAPDH:

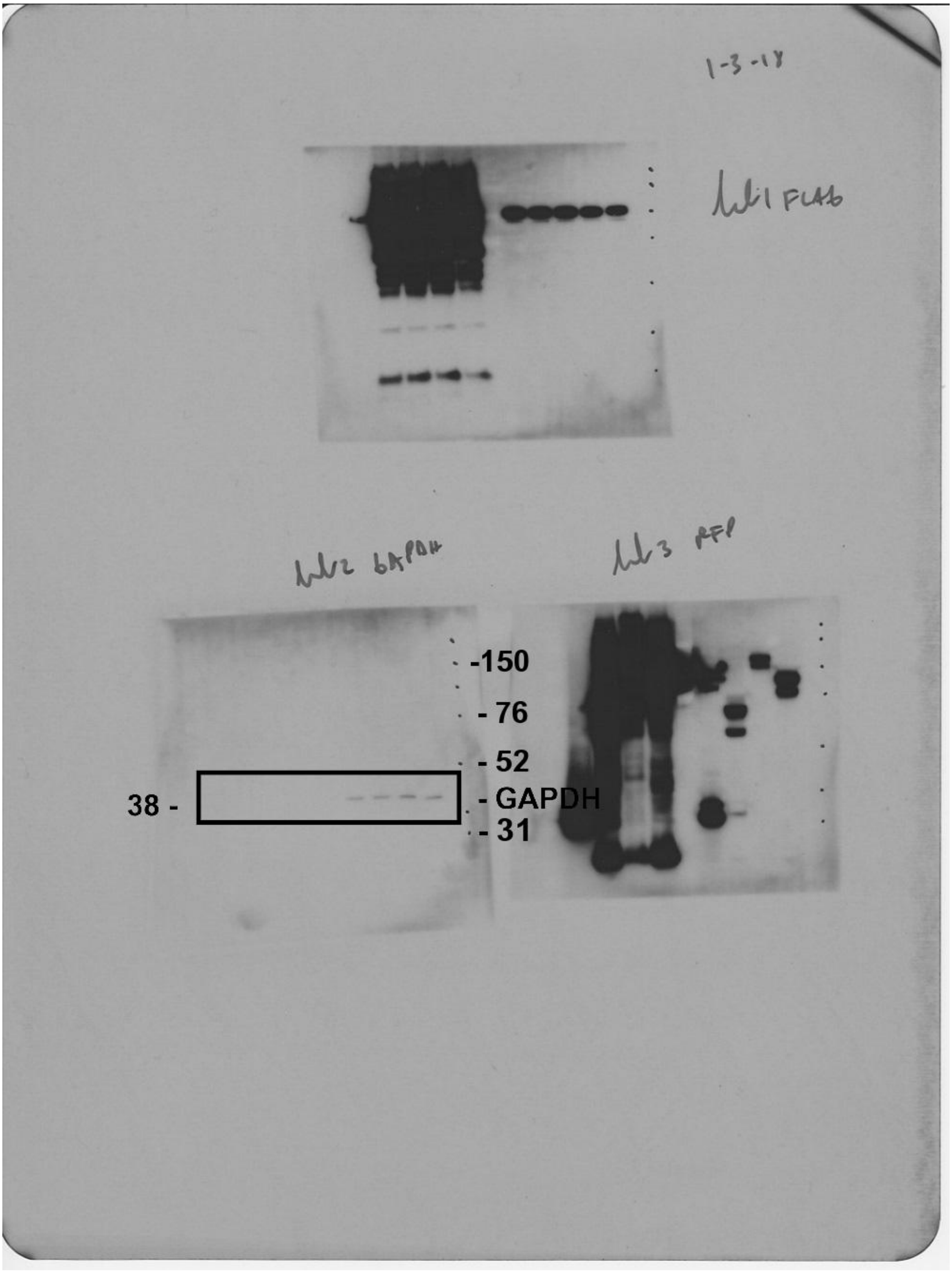

Figure 2c IP-MIC60-FLAG (upper panel left):

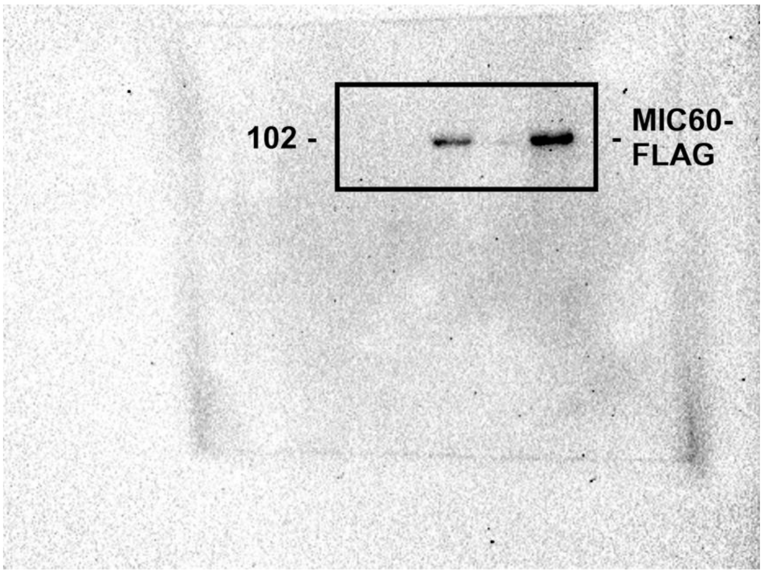

Figure 2c WCL-MIC60-FLAG (upper panel right):

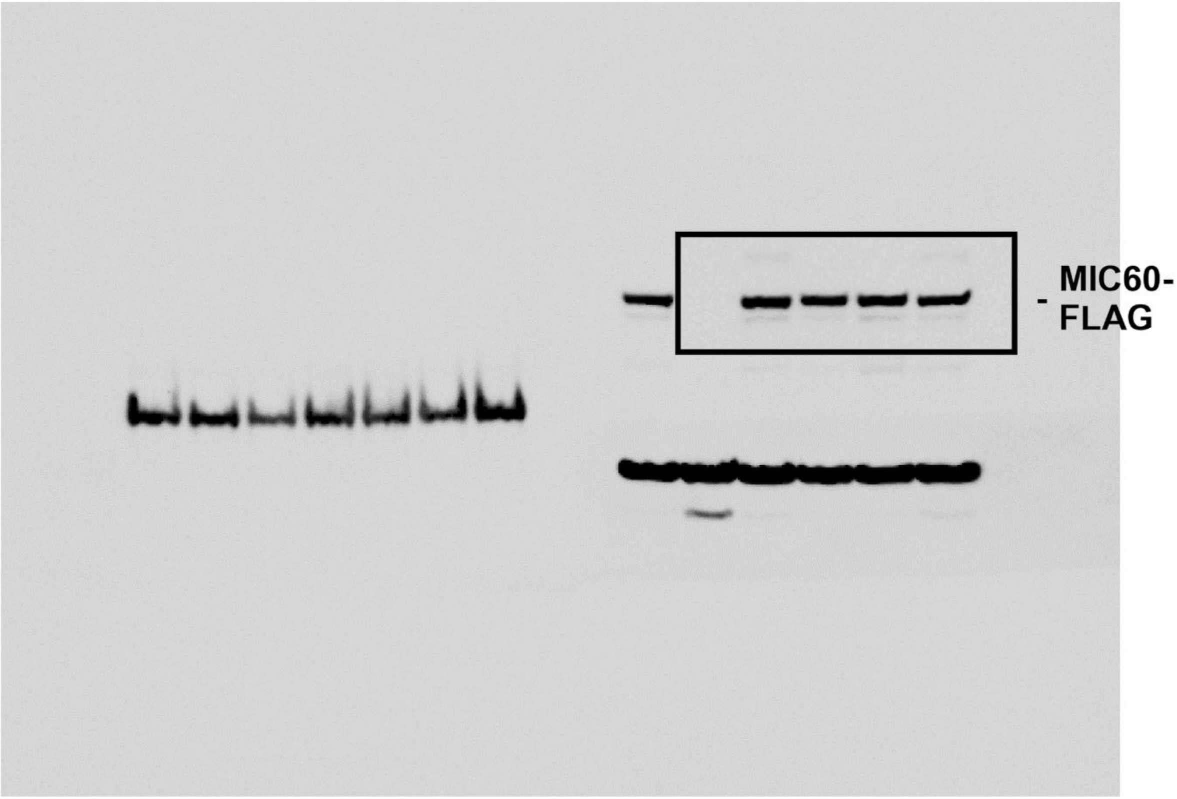

Figure 2c IP (middle panel left) and WCL-RFP (middle panel right):

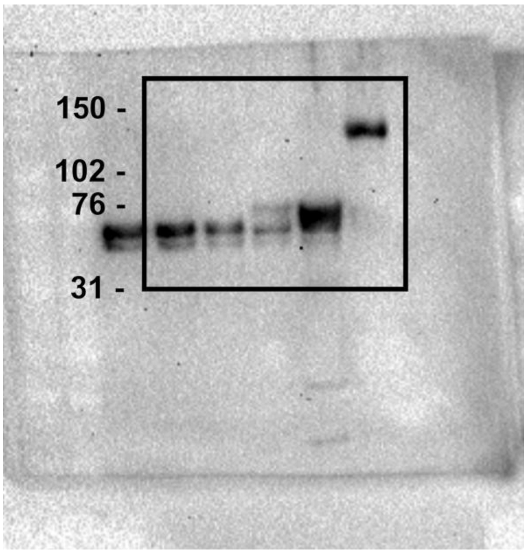

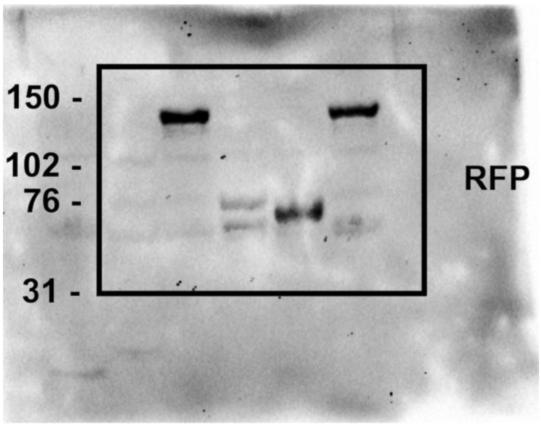

Figure 2c GAPDH (lower panel right)

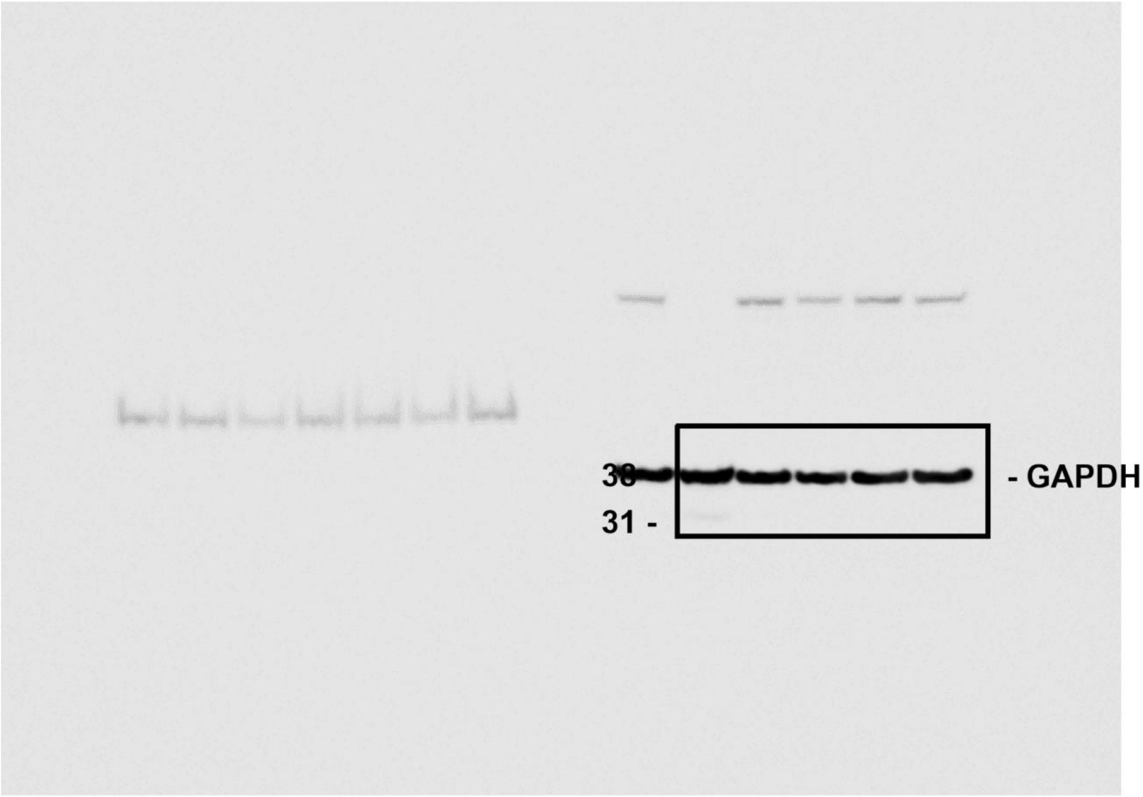

Figure 3: Original Western-Blot scans

Fig 1 C upper panel MIC 60

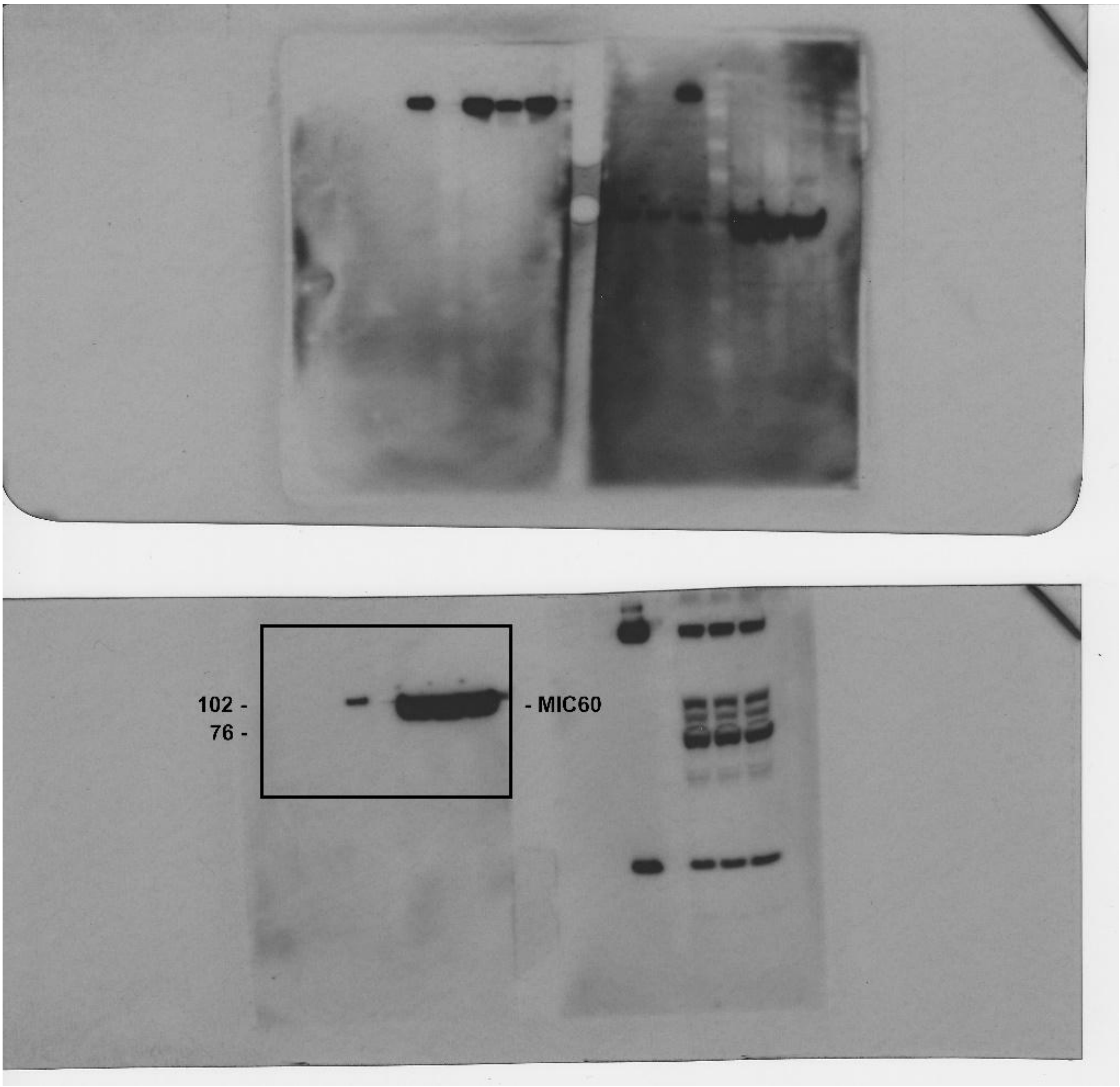

Figure 3: Original Western-Blot scans

Figure 3a panel RRx (upper):

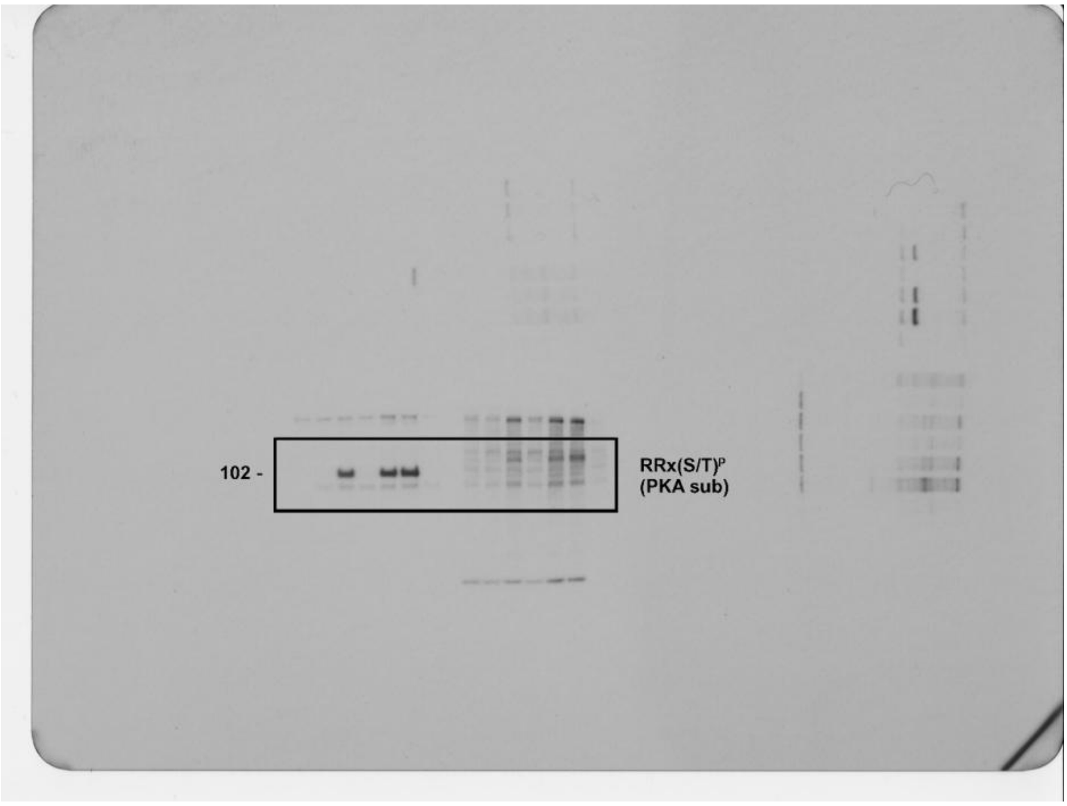

Figure 3a panel RRx (lower):

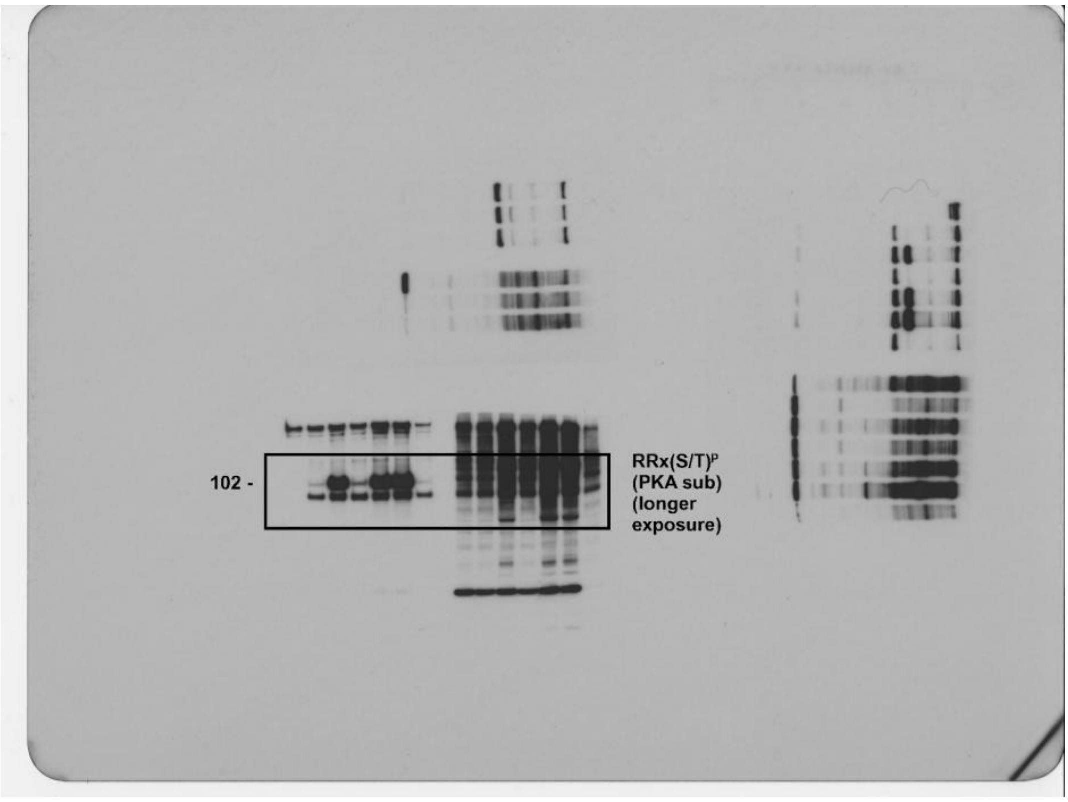

Figure 3a panel MIC60-FLAG:

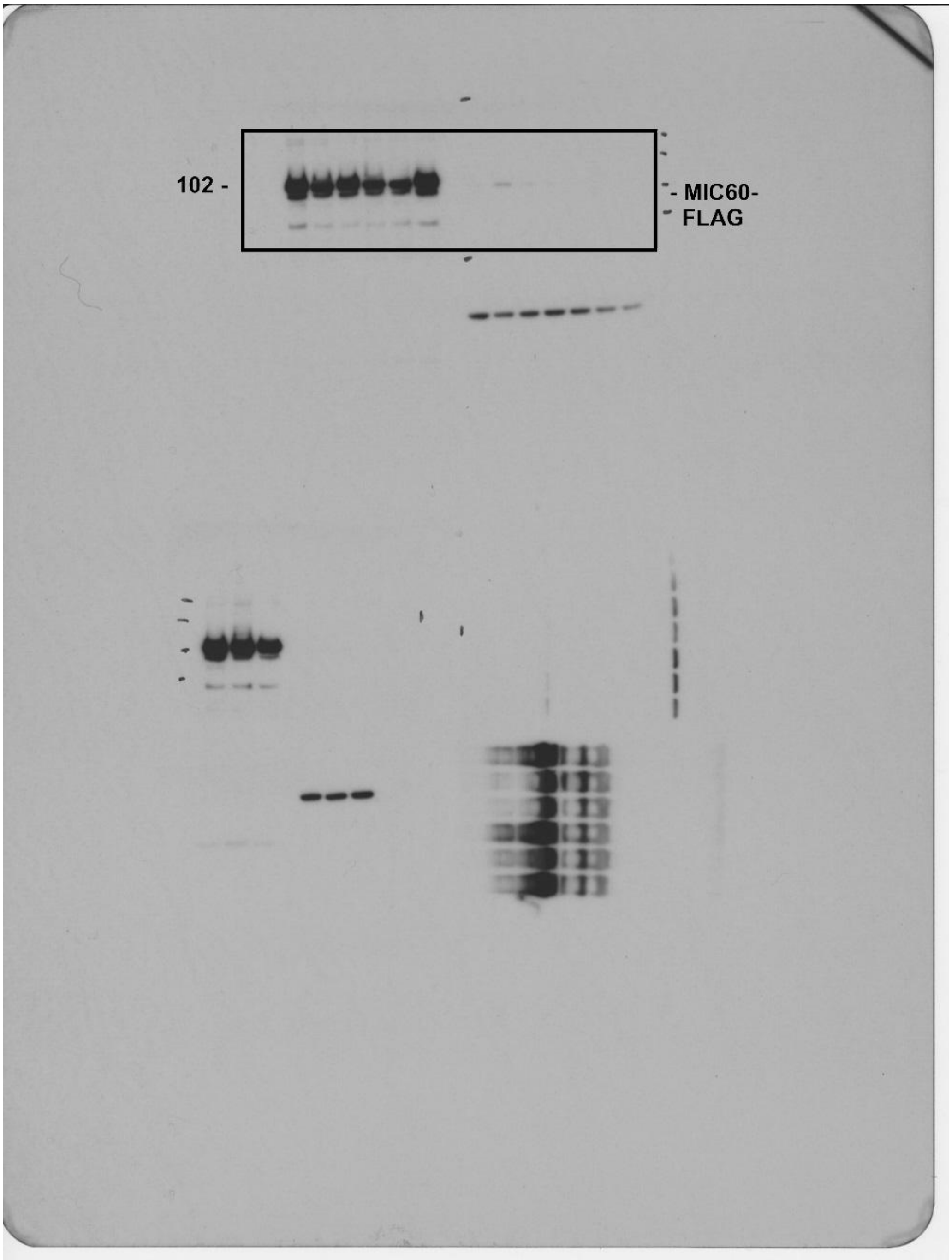

Figure 3a panel GAPDH:

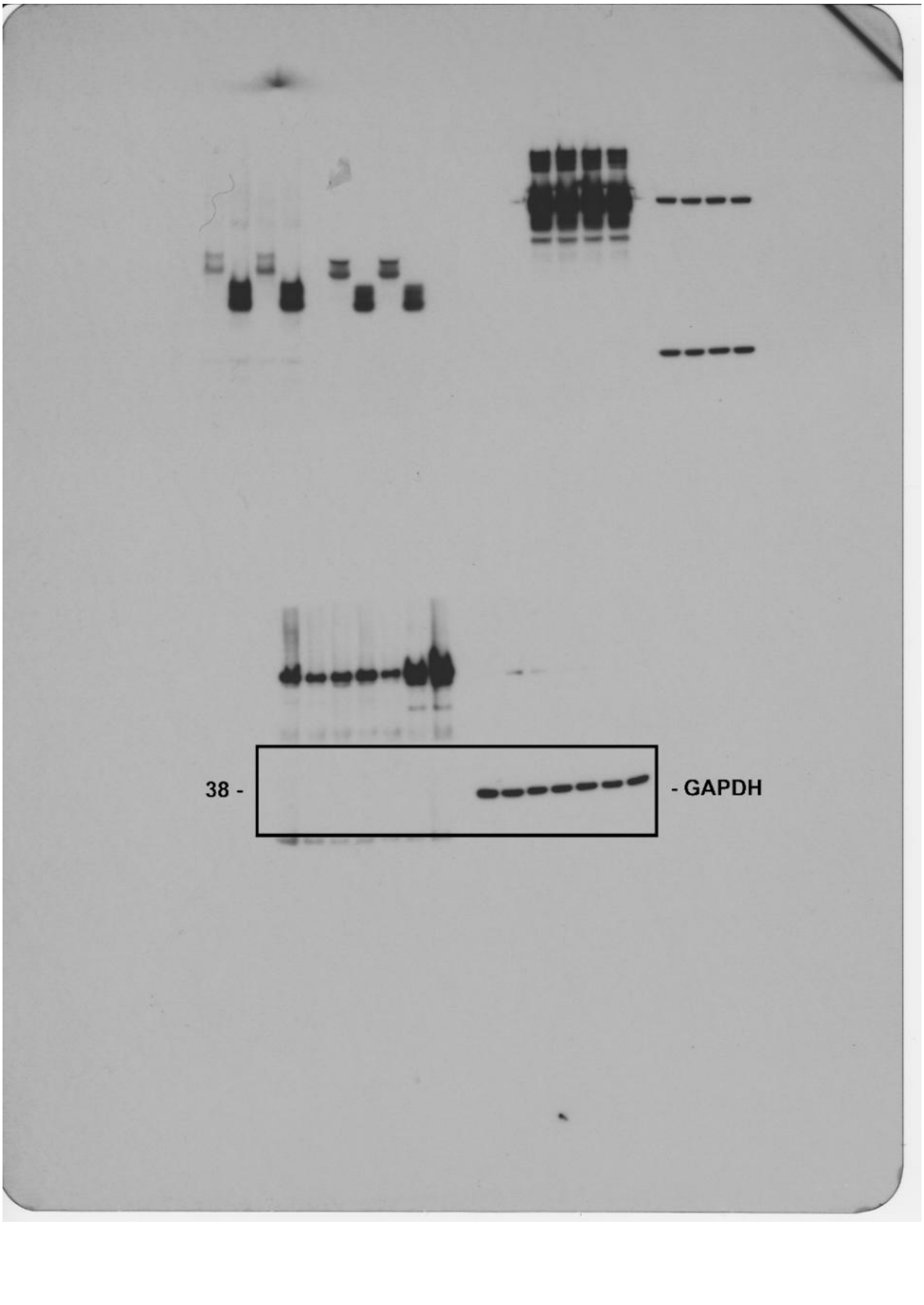

Figure 3d panel RRx:

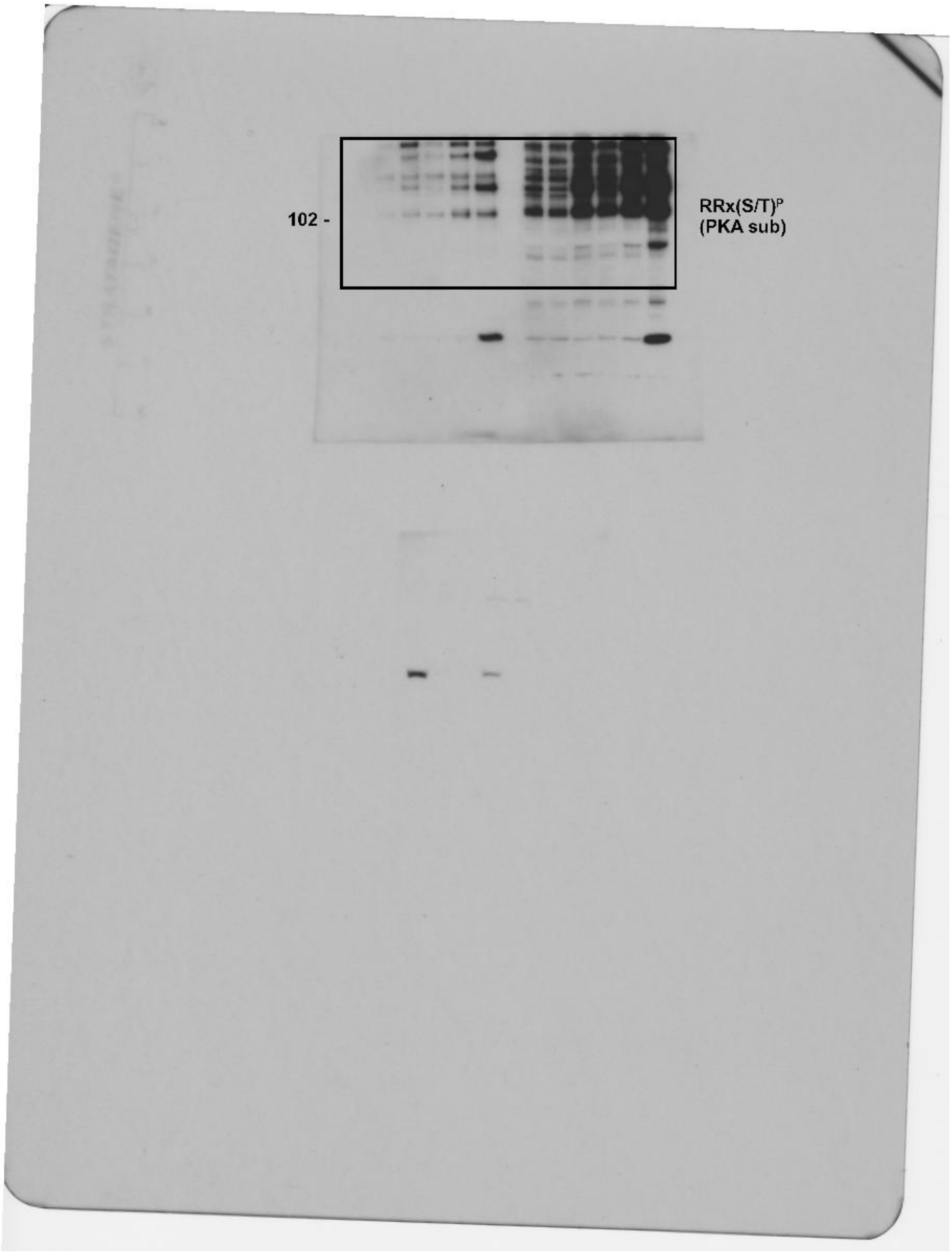

Figure 3d panel MIC60-FLAG:

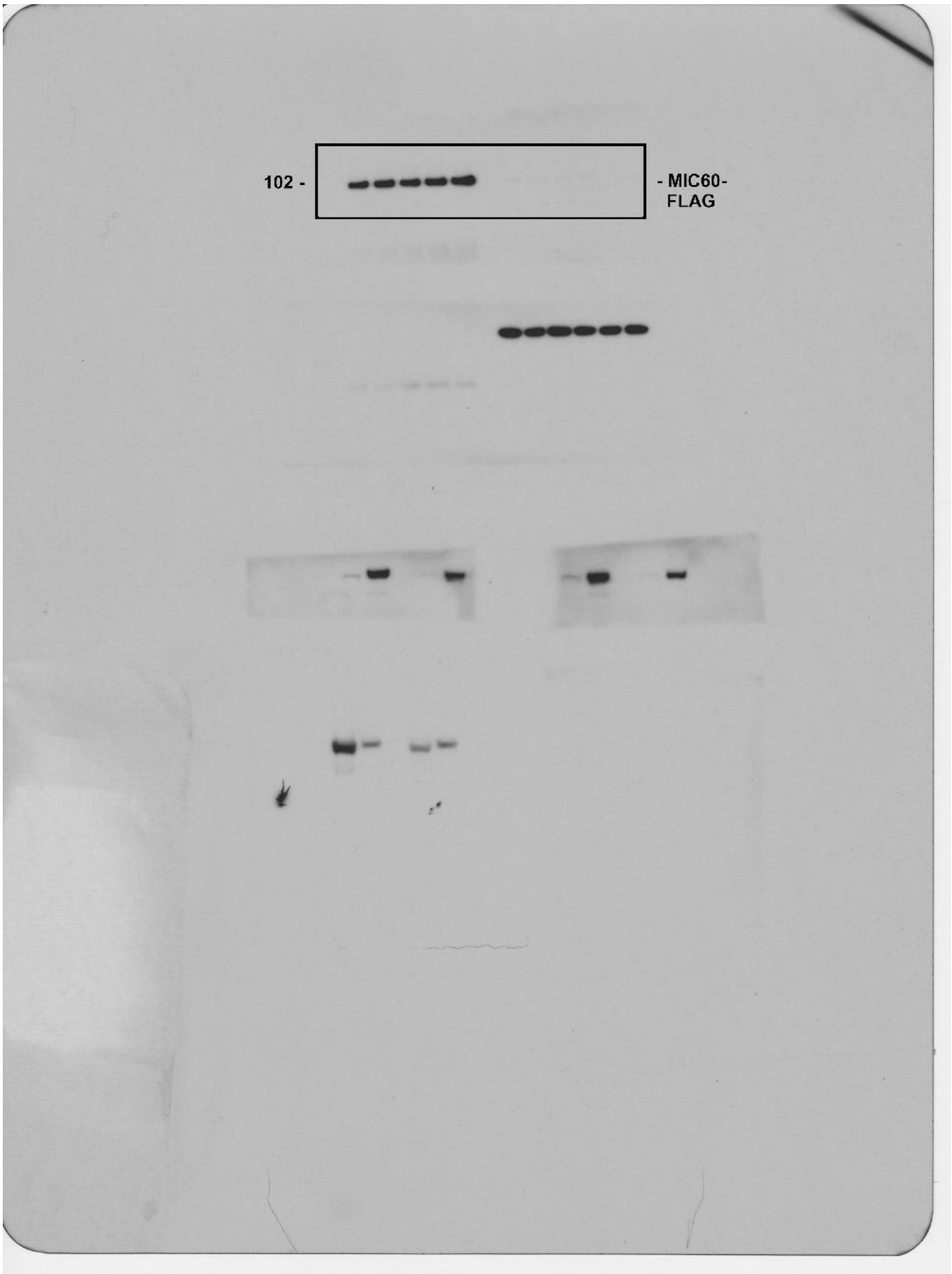

Figure 3d panel GAPDH:

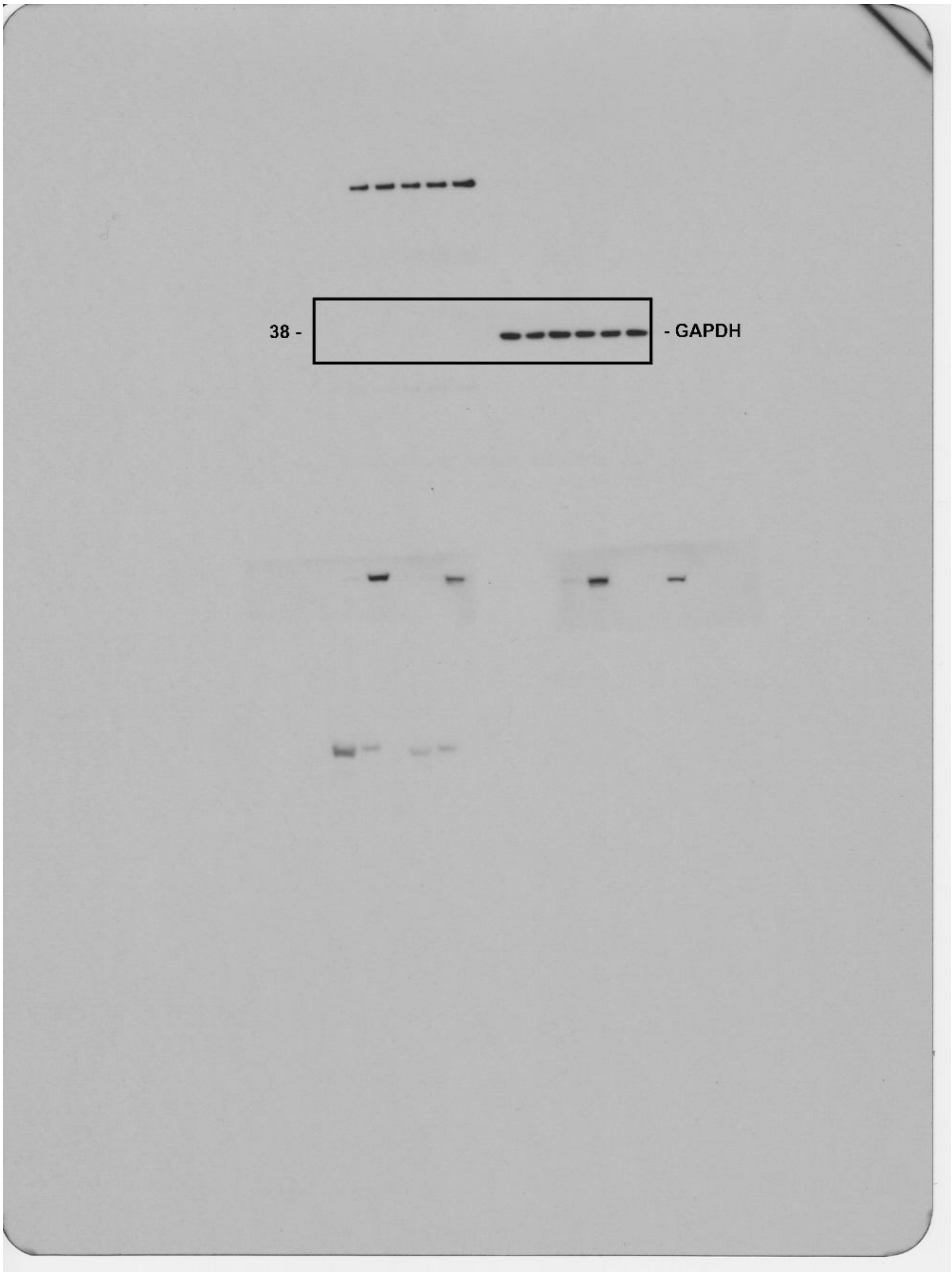

Figure 3f panel RRx:

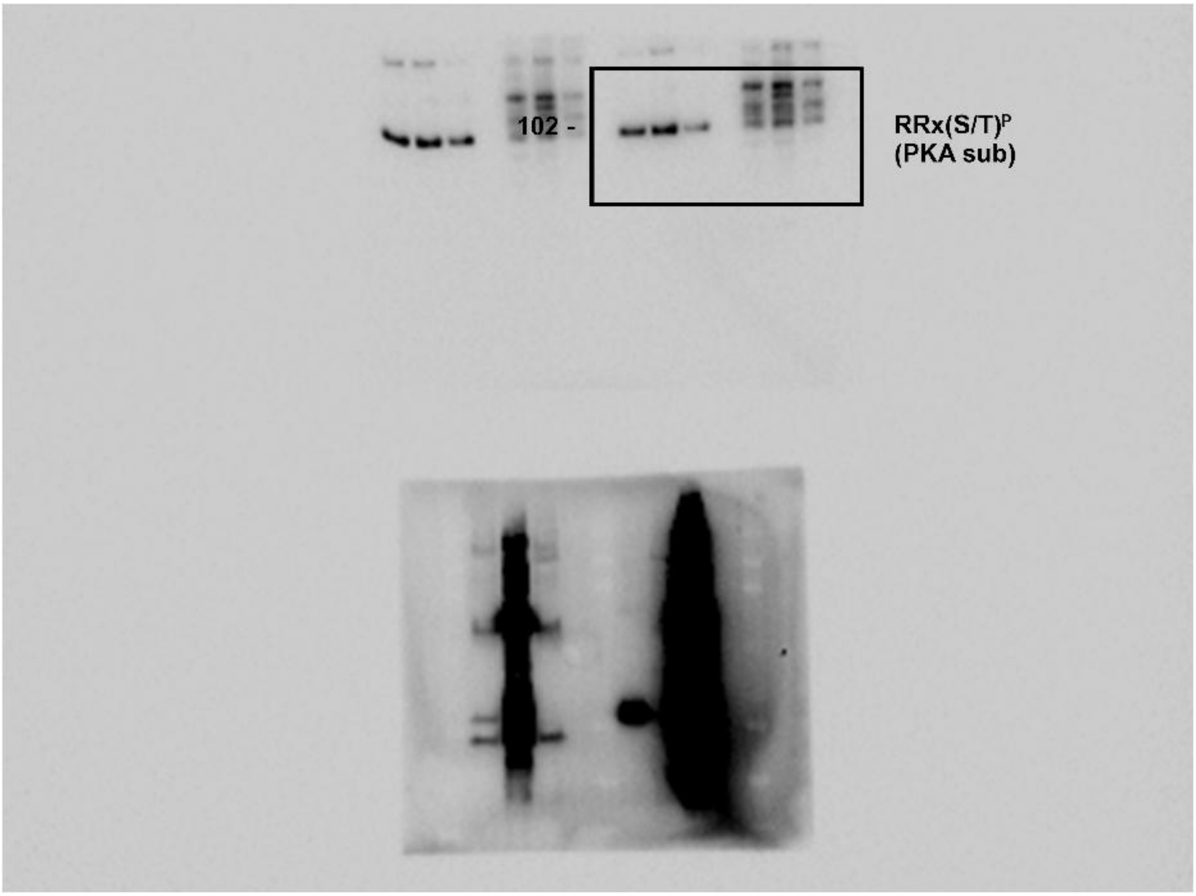

Figure 3f panel FLAG:

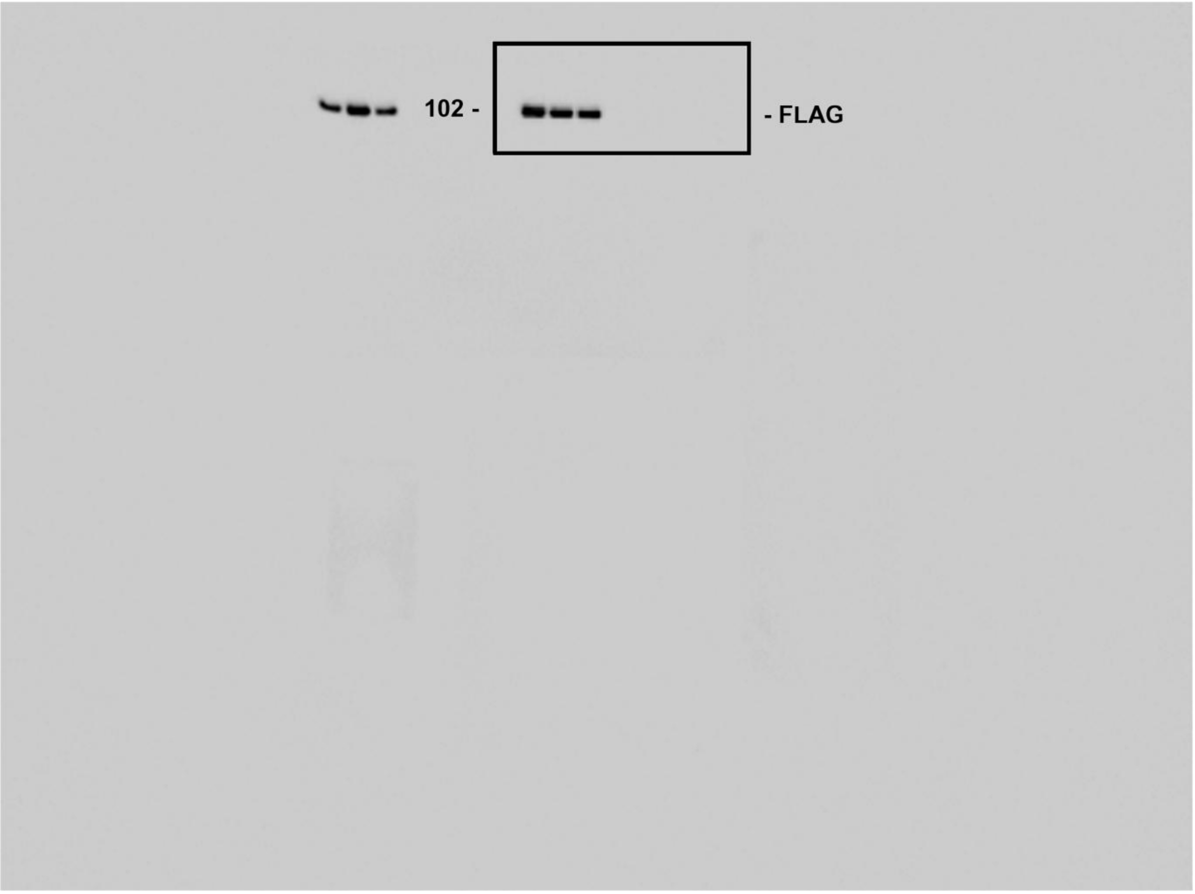

Figure 3f panel RRX:

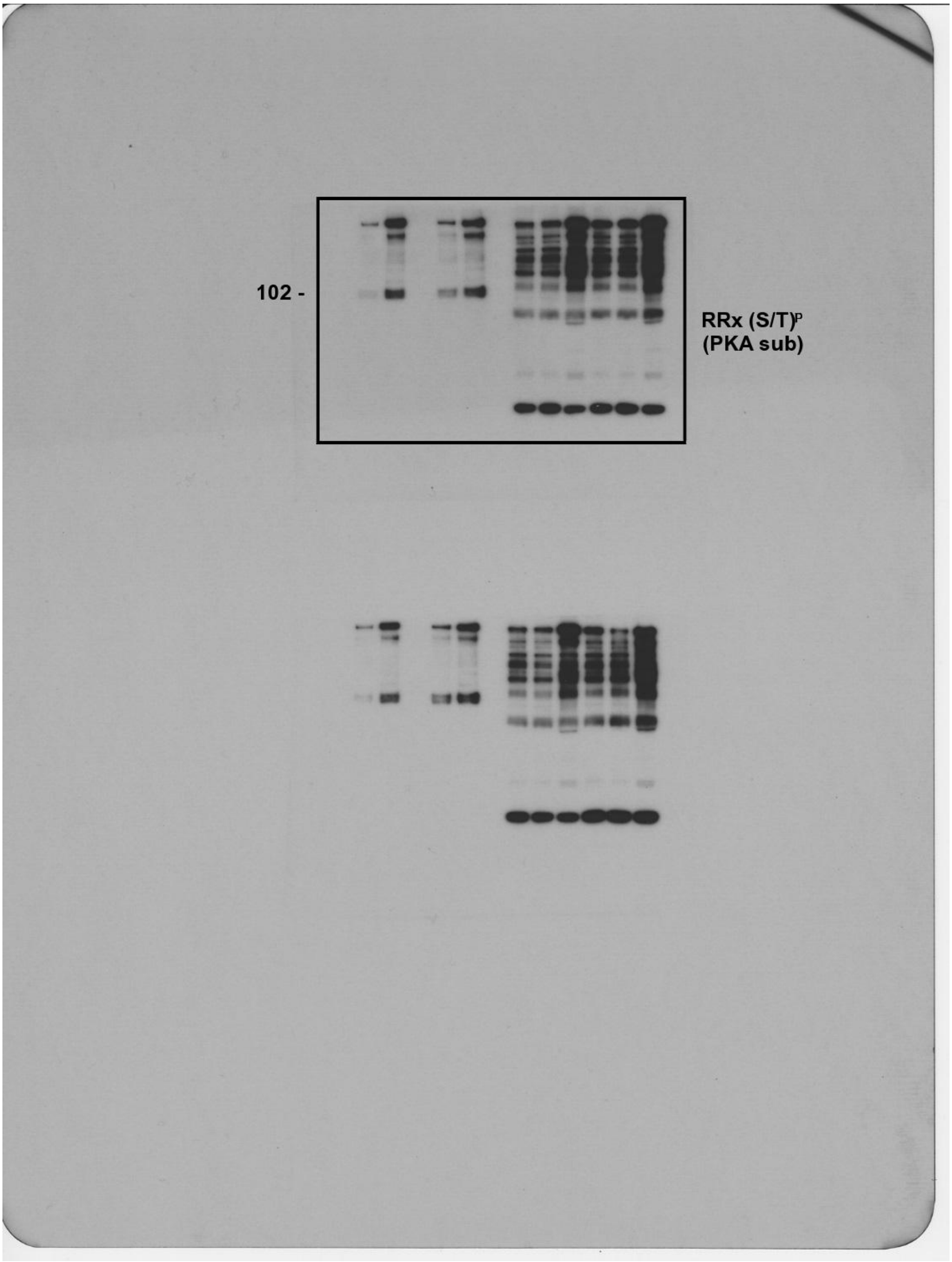

Figure 3f panel MIC60-FLAG and GAPDH:

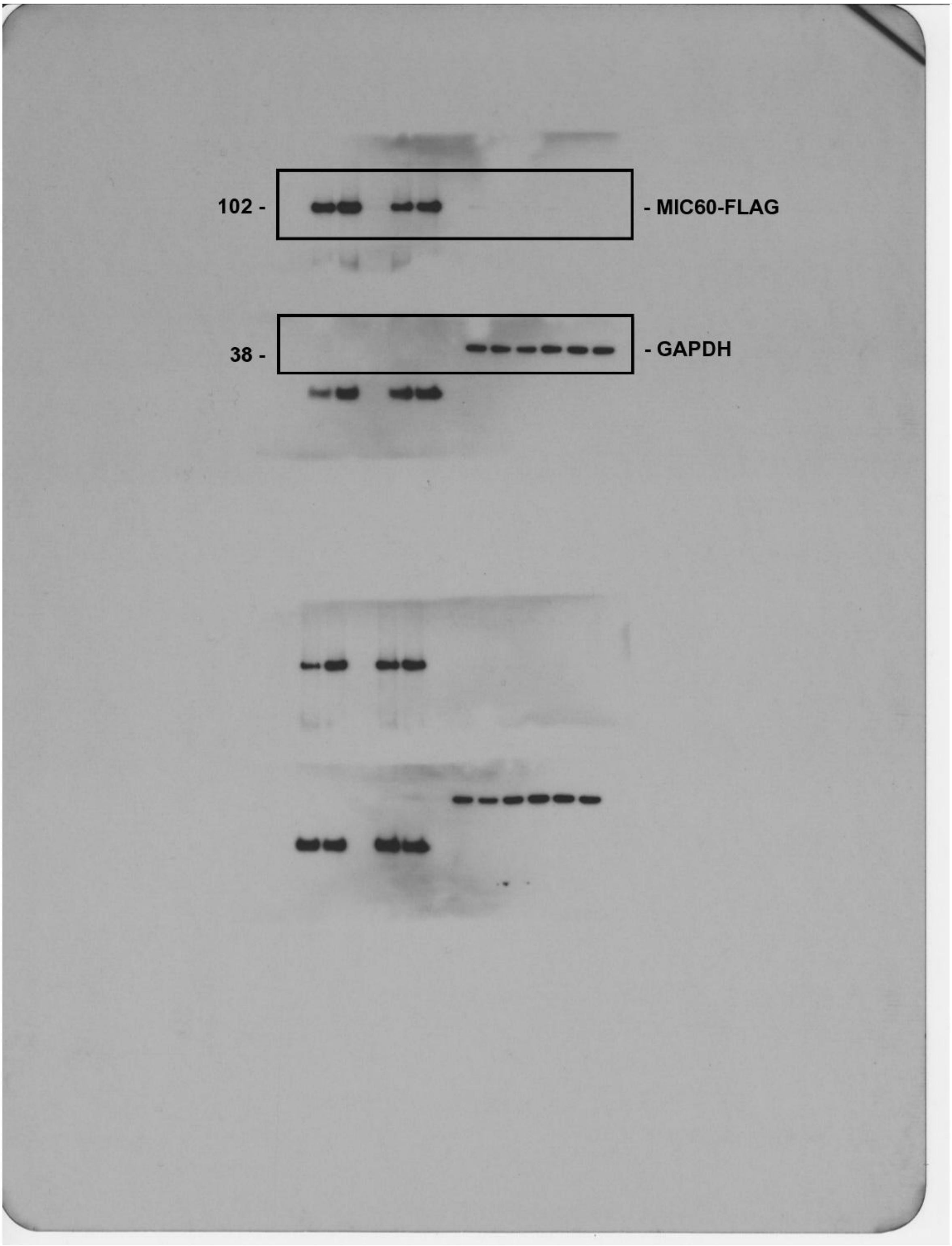

Figure 7: Original Western-Blot scans

Figure 7a panels PINK1 and GAPDH:

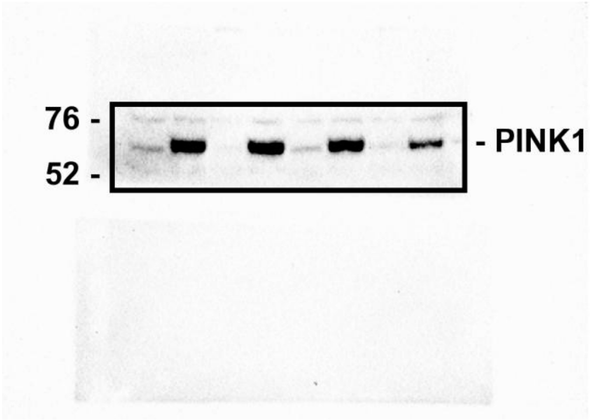

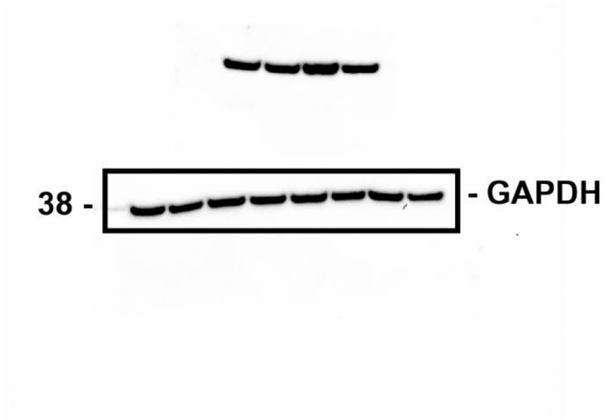

Figure 7c panel RFP:

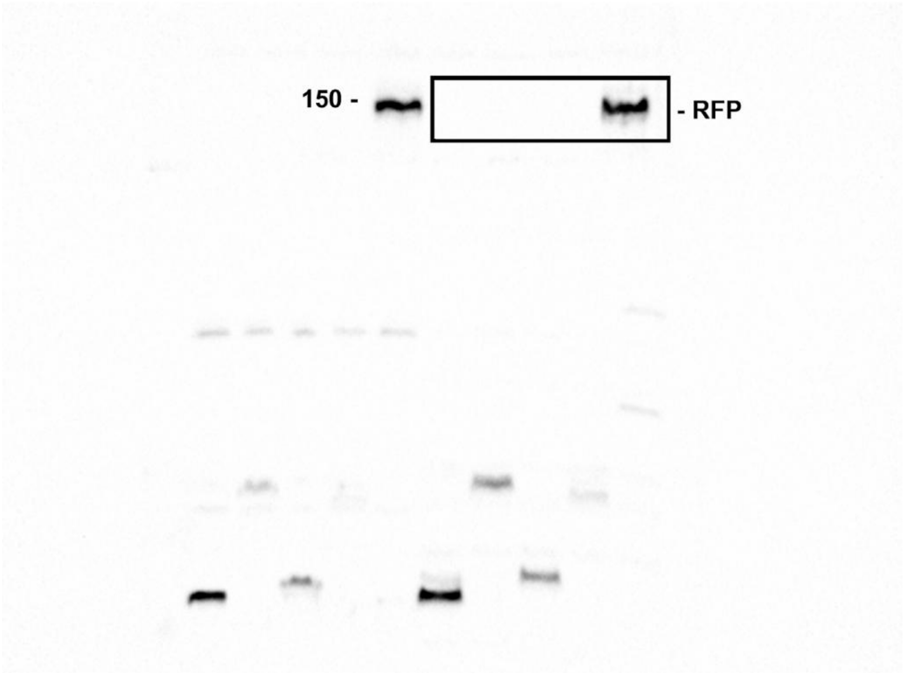

Figure 7c panel PINK1

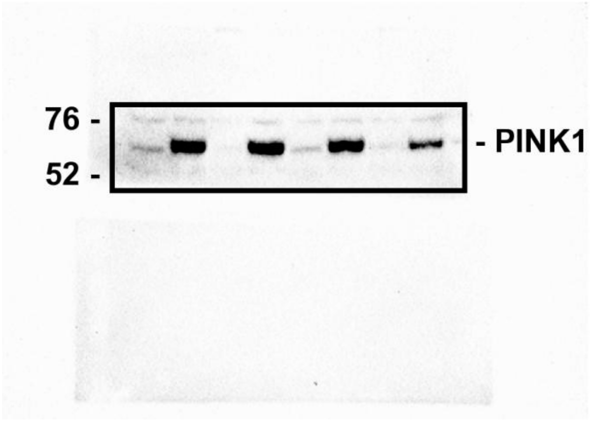

Figure 7c panel GAPDH

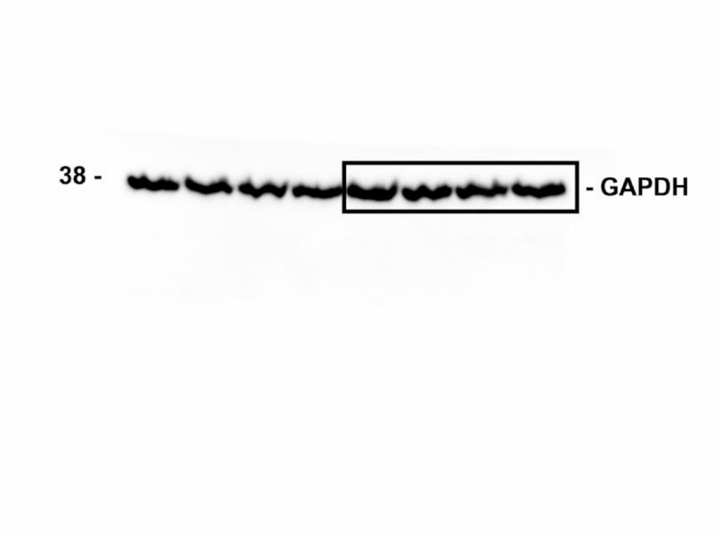

Figure 8: Original Western-Blot scans

Figure 8a PINK1:

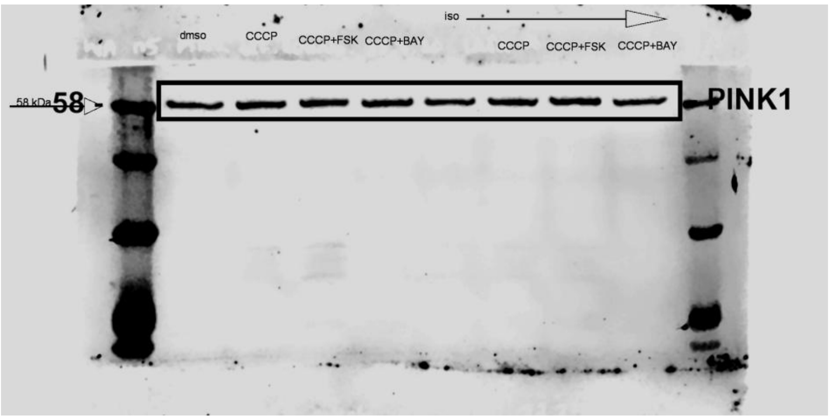

Figure 8a Calnexin:

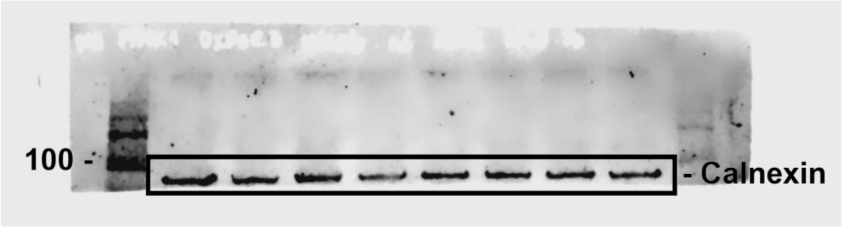

Figure 8c UCP1:

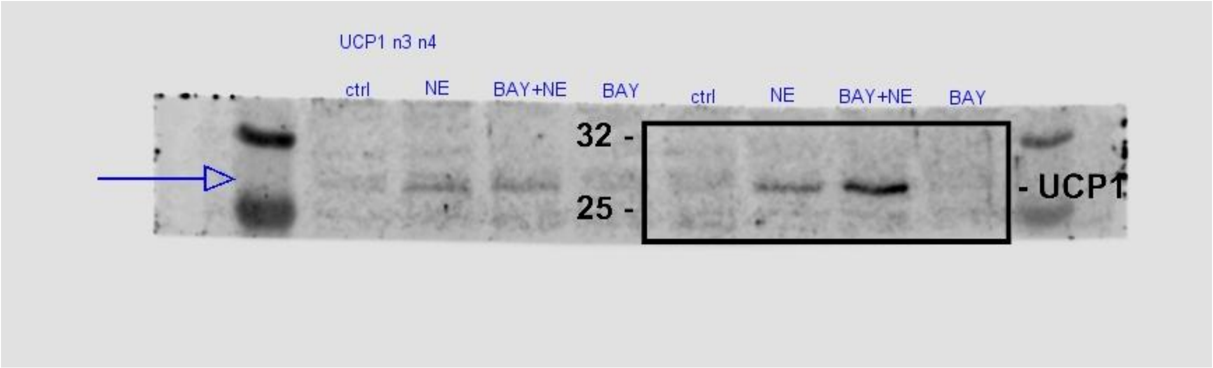

Figure 8c Calnexin:

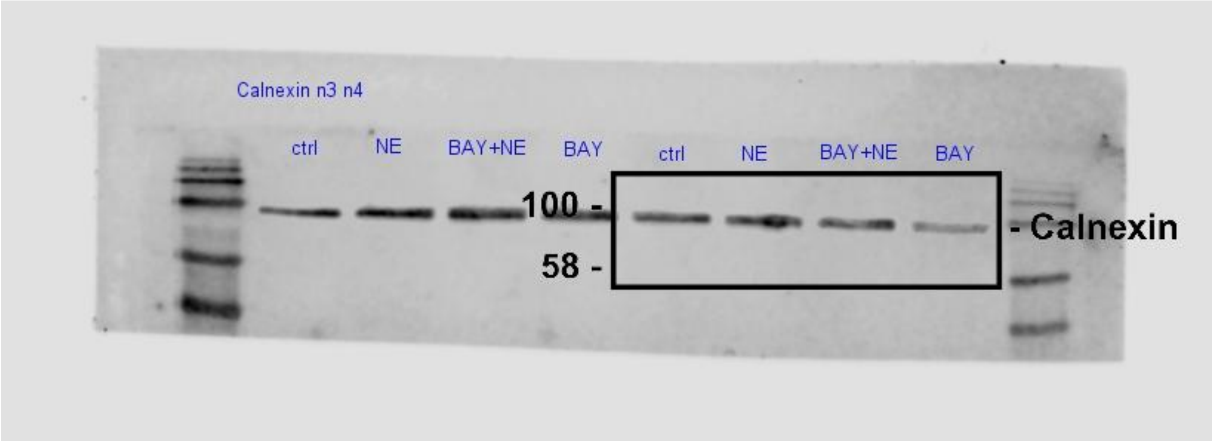

Original Western-Blot Scans - Supplement

Original scans Supplemental Figure 1

Supplemental figure 1a Mic60 FLAG:

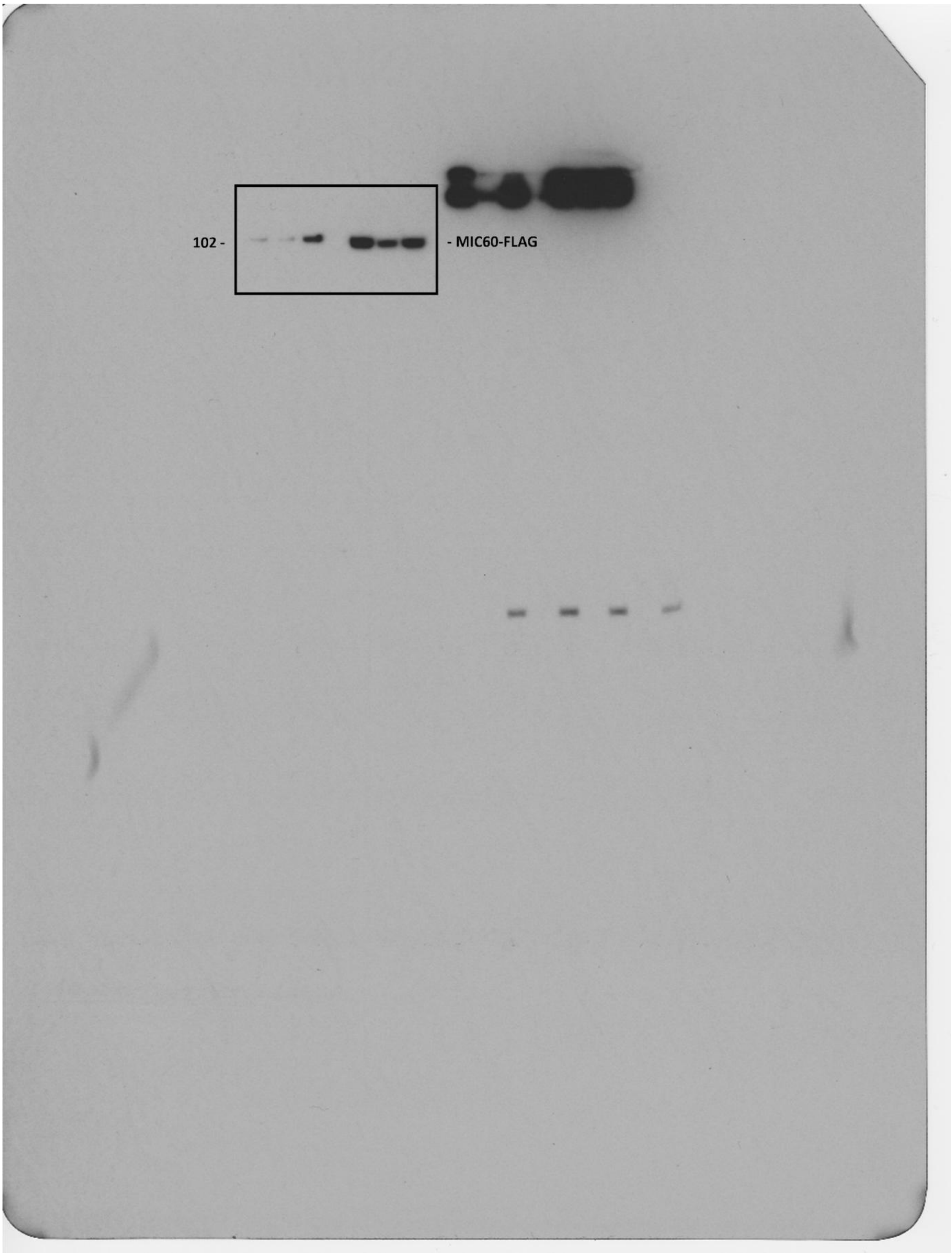

Supplemental figure 1a RFP (middle panel left) and GAPDH (lower panel):

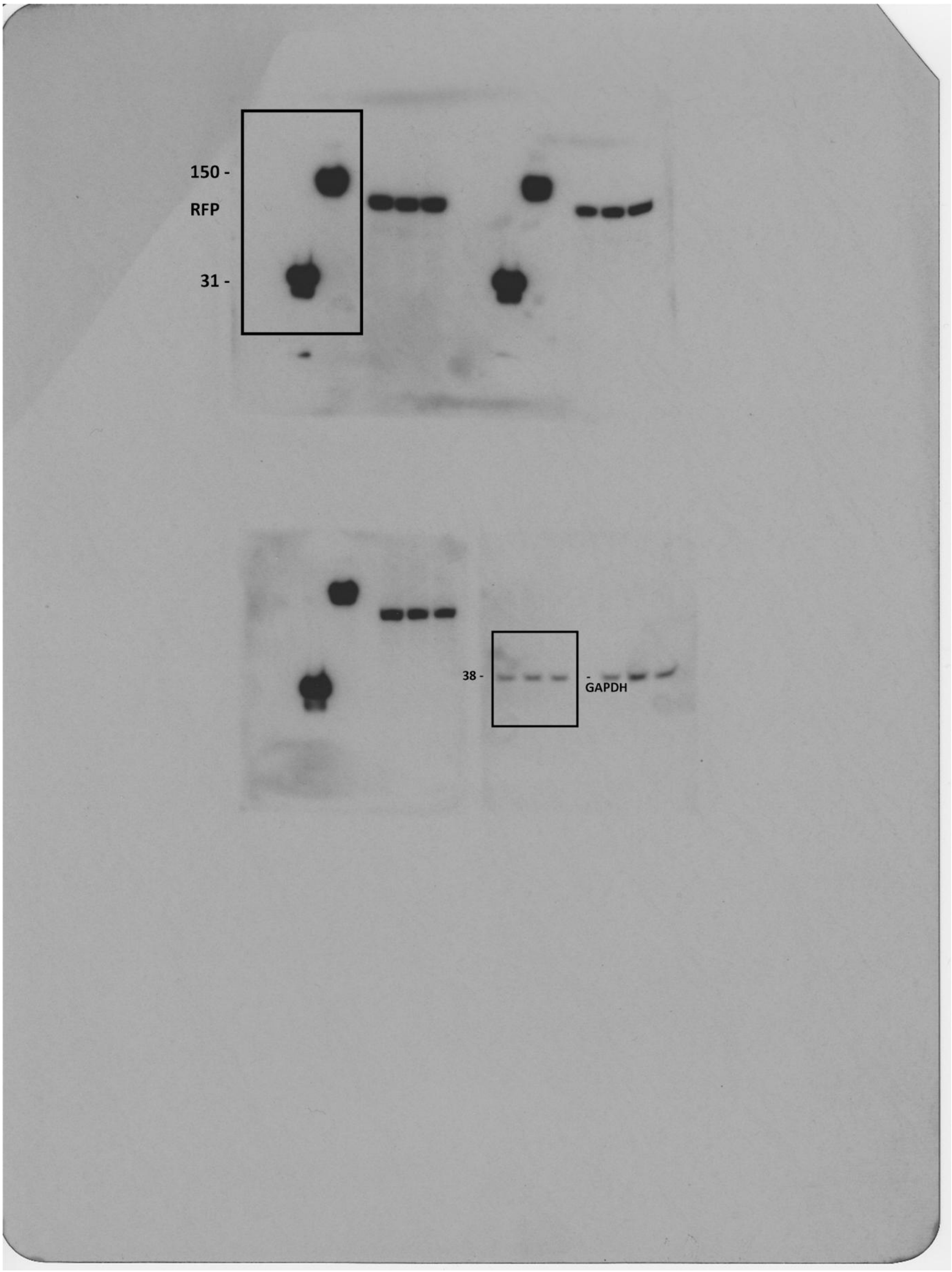

Supplemental figure 1a PDE2A2 (middle panel right):

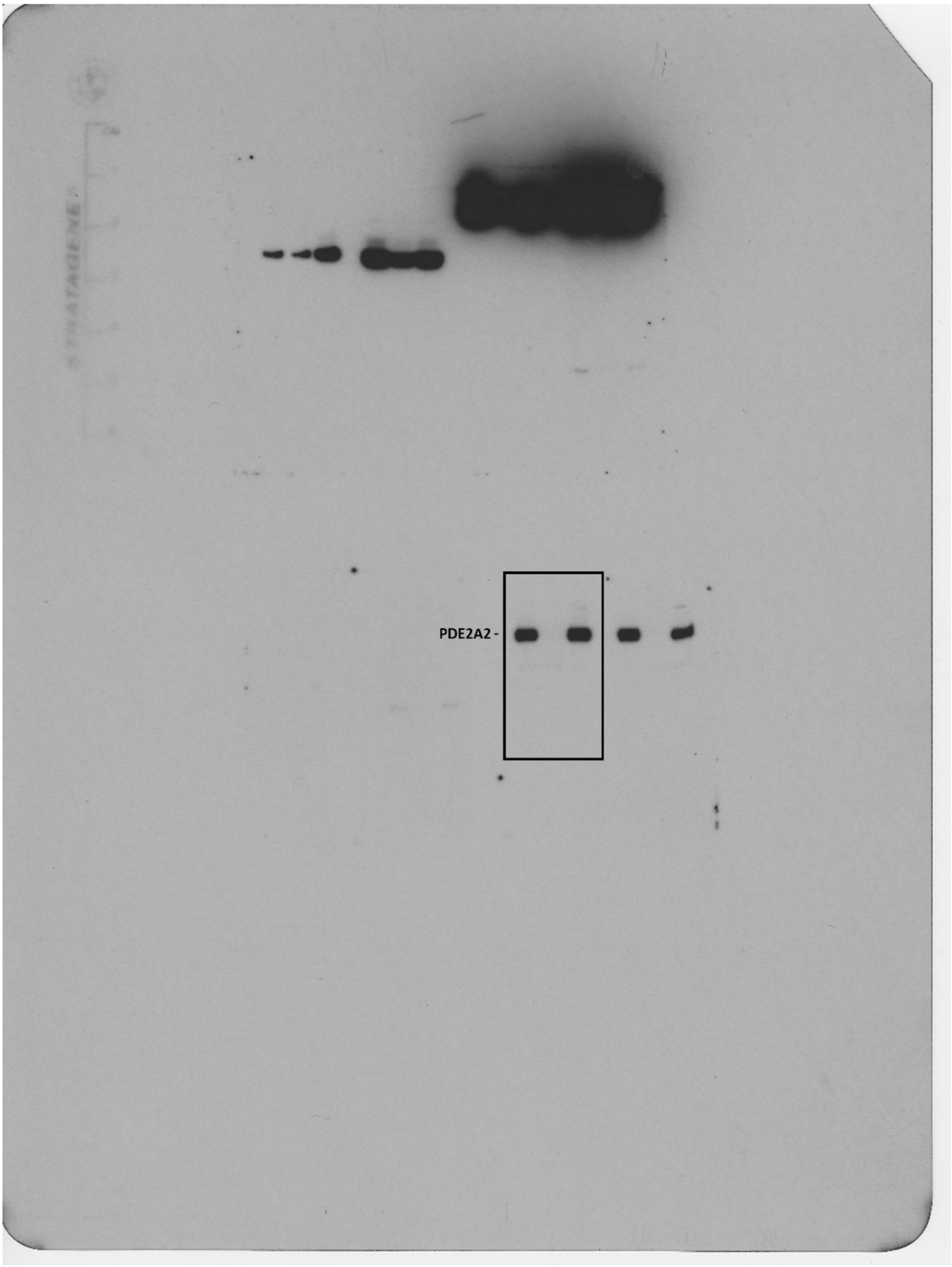

Supplemental figure 1b GFP (upper panel):

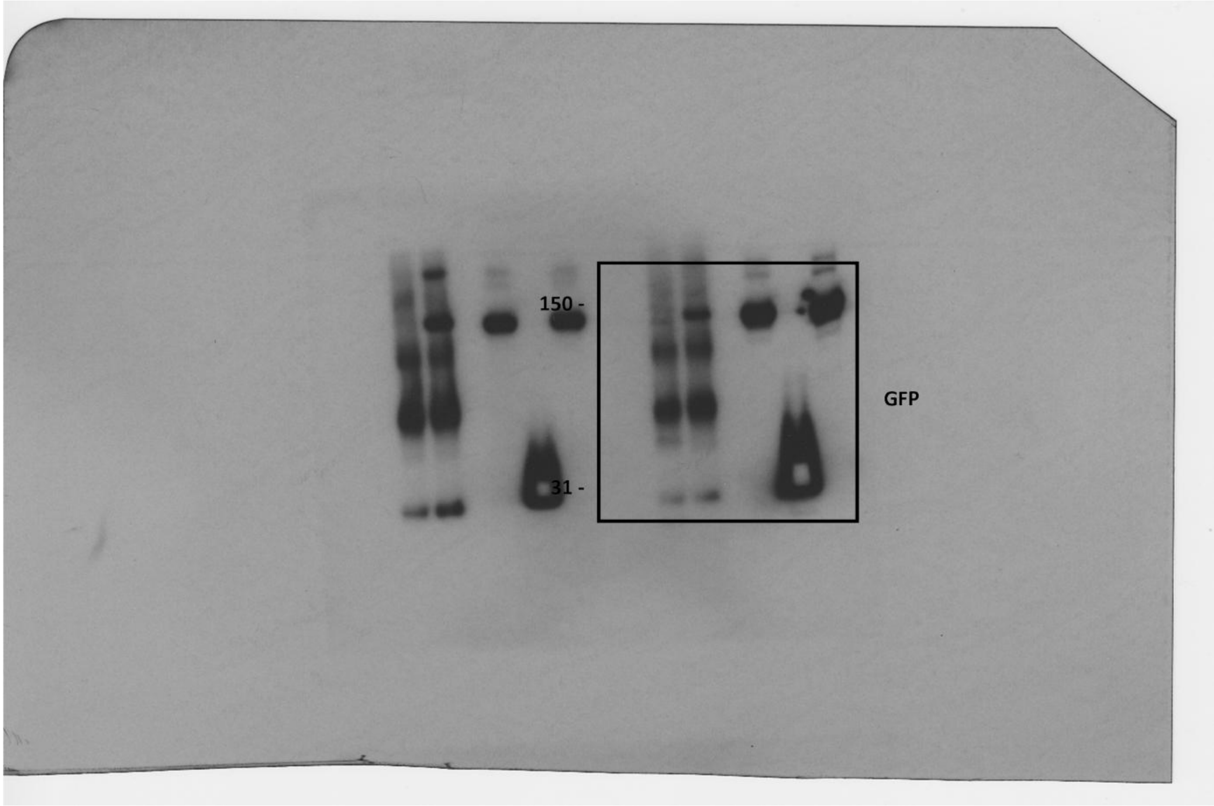

Supplemental figure 1b MIC60-FLAG (middle panel):

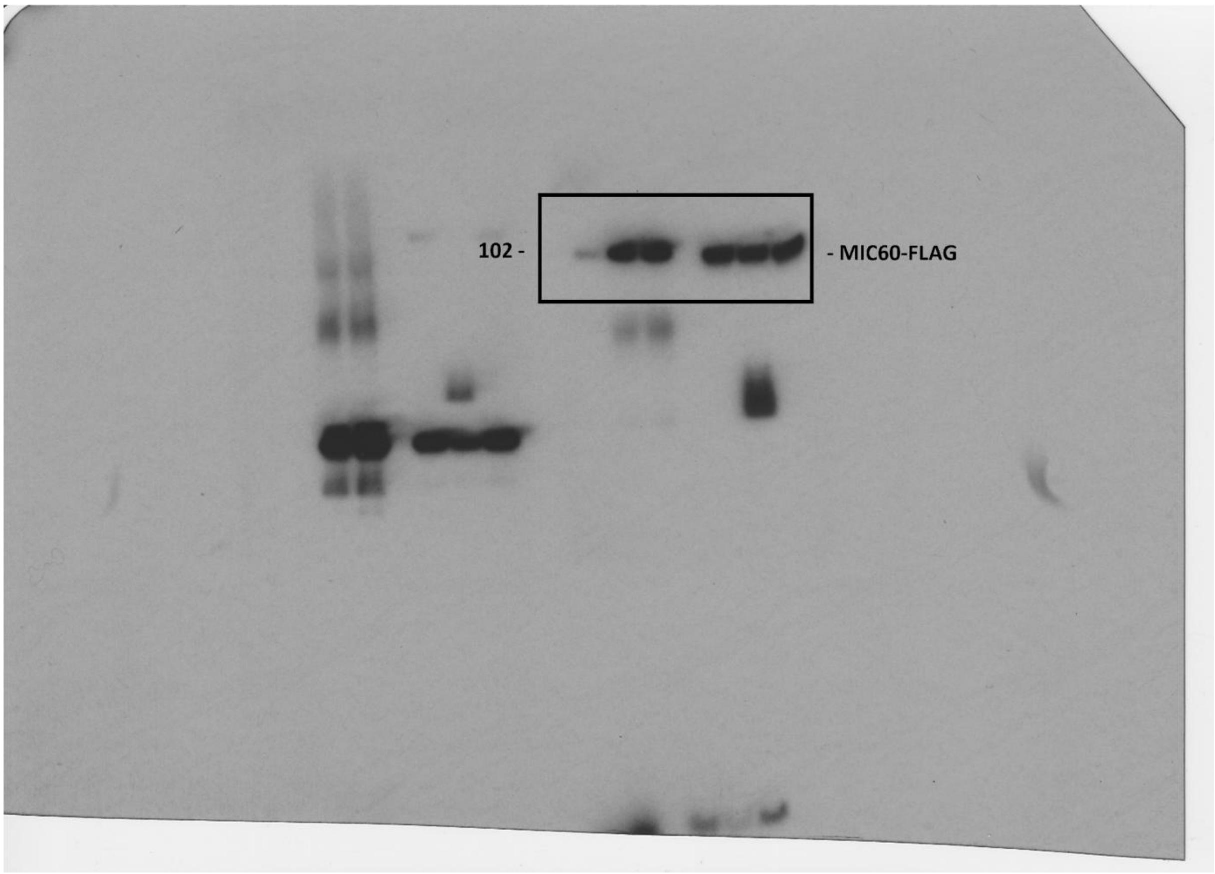

Supplemental figure 1b GAPDH (lower panel):

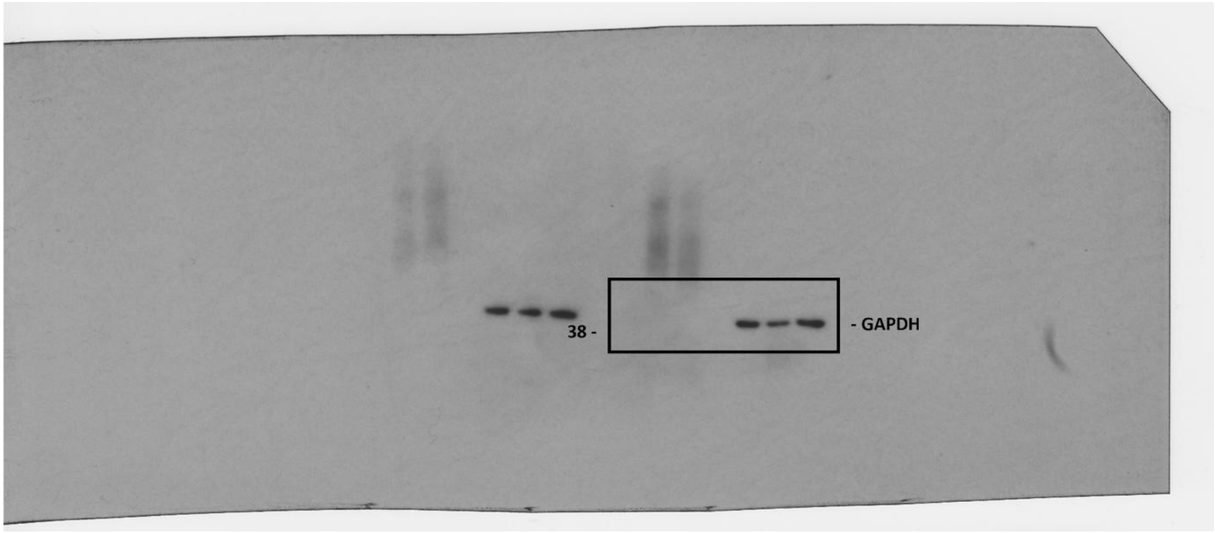

Supplemental figure 1c GFP (upper panel):

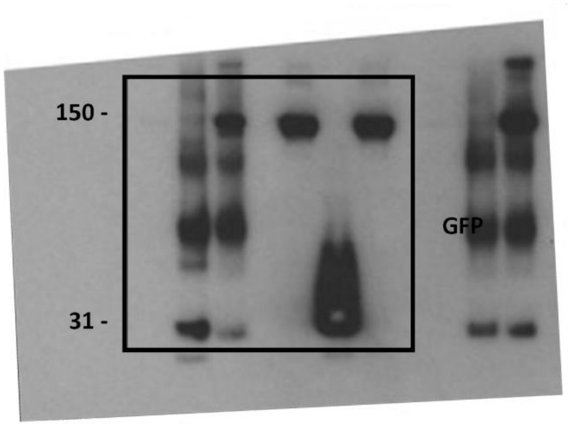

Supplemental figure 1c SAMM50-FLAG (middle panel):

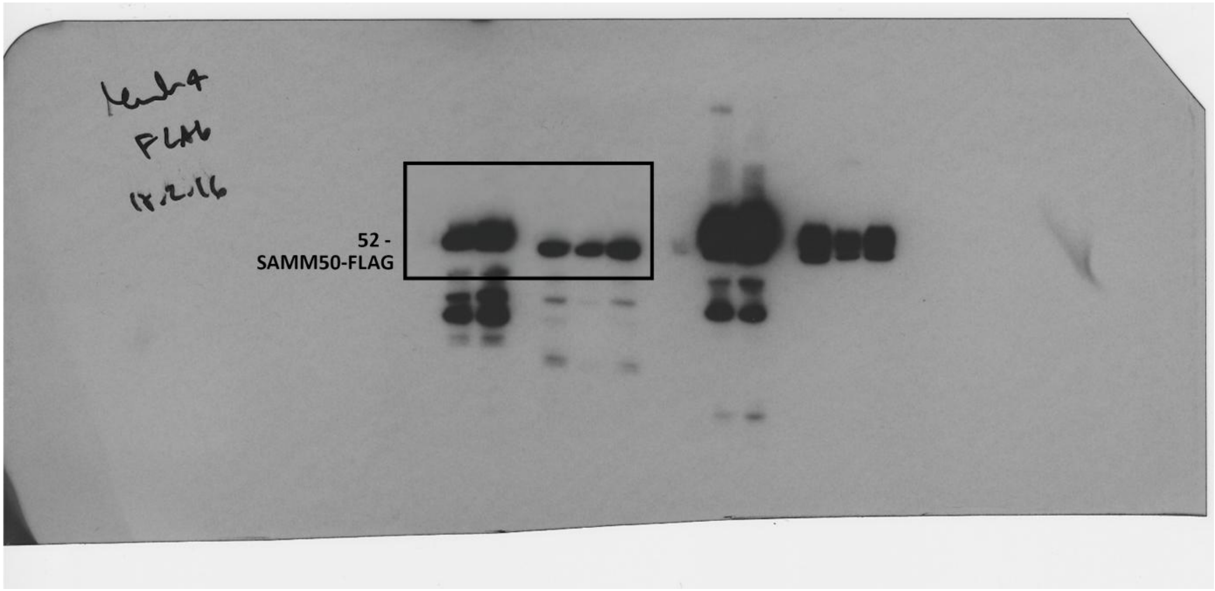

Supplemental figure 1c GAPDH (lower panel):

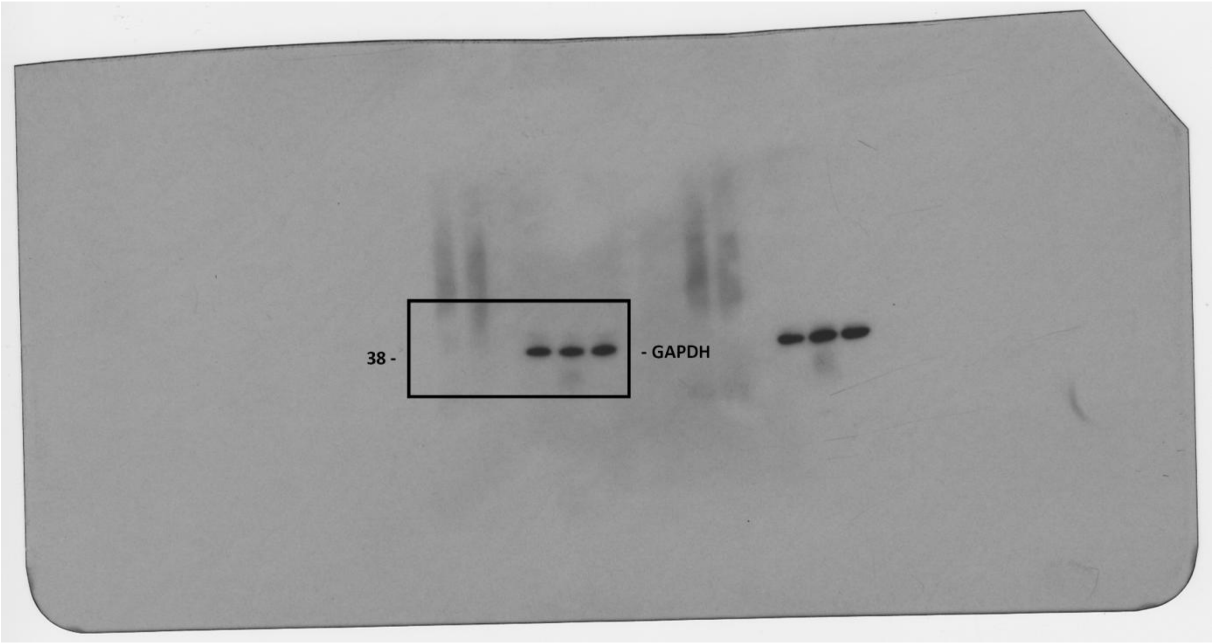

Supplemental figure 1d GFP (upper panel):

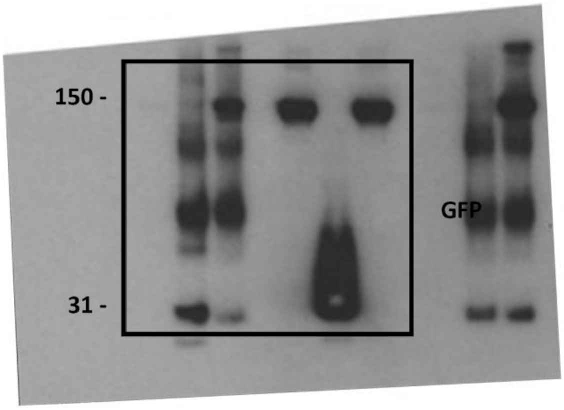

Supplemental figure 1d FLAG-IP (lower panel left):

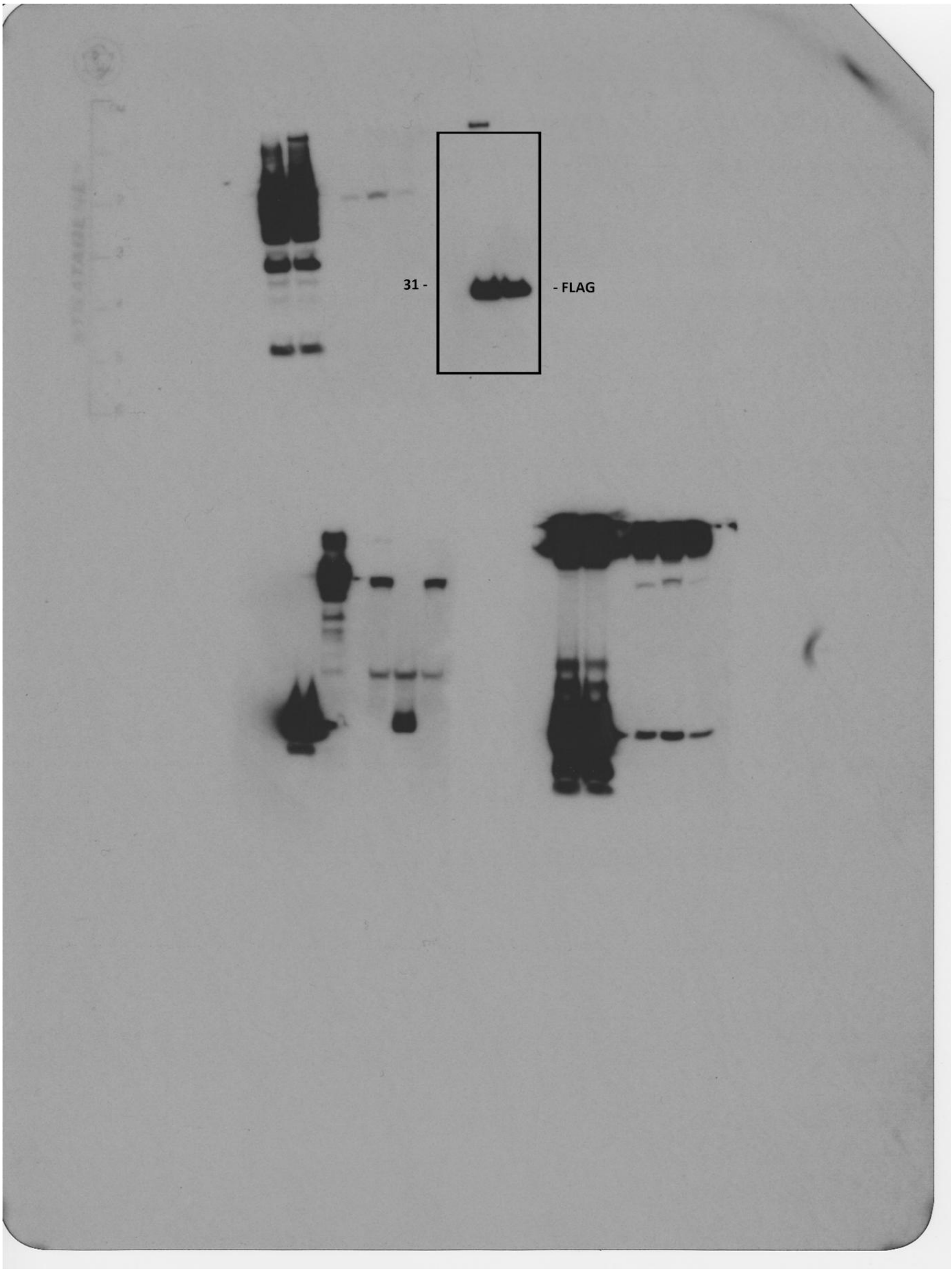

Supplemental figure 1d FLAG-WCL (lower panel right):

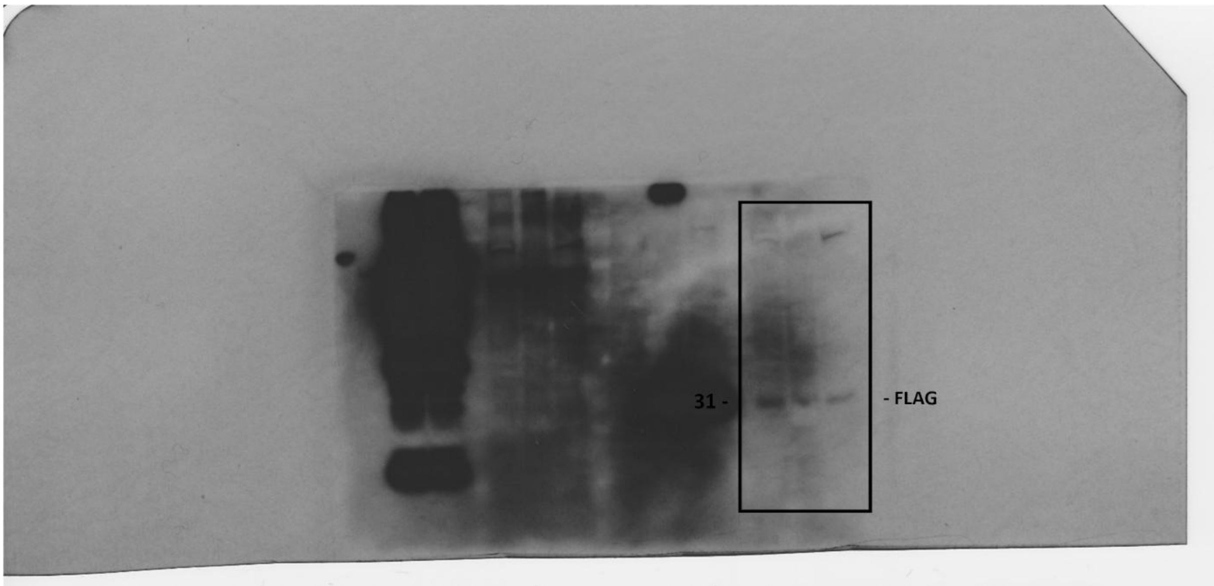

Original scans Supplemental Figure 2

Supplemental figure 2b RFP (left upper panel):

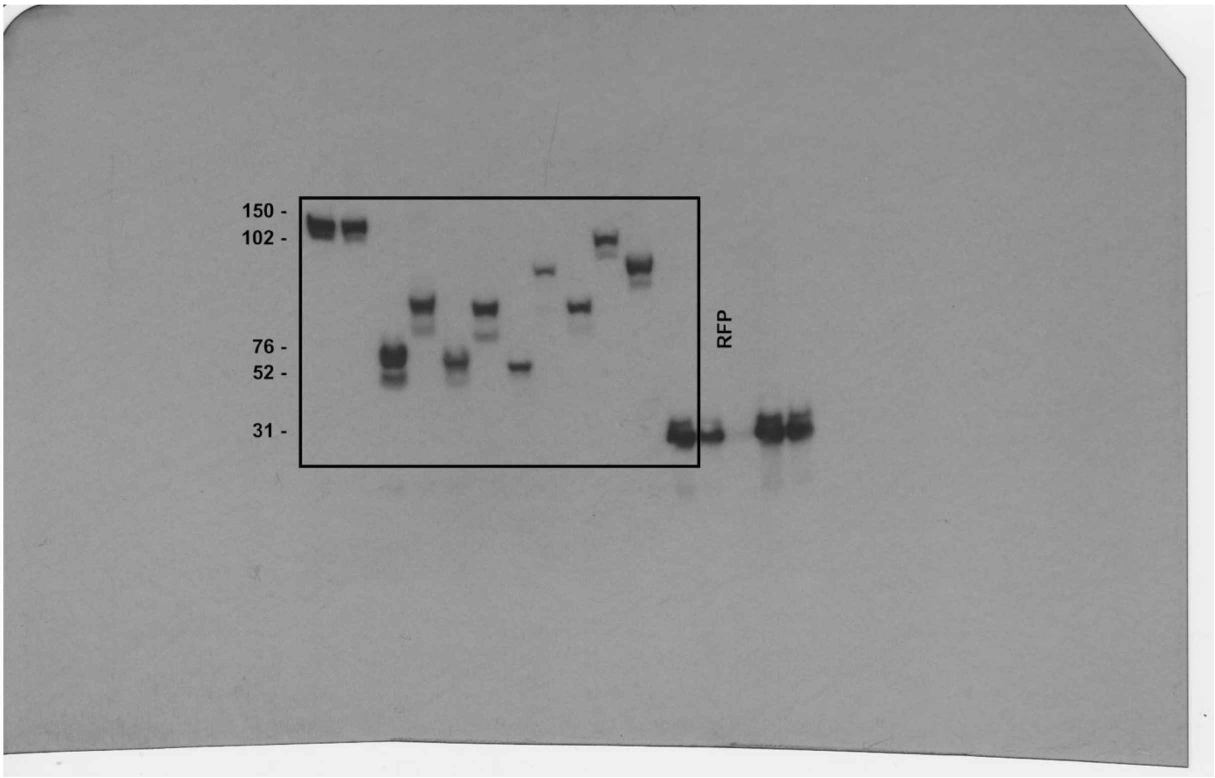

Supplemental figure 2b GAPDH (left lower panel):

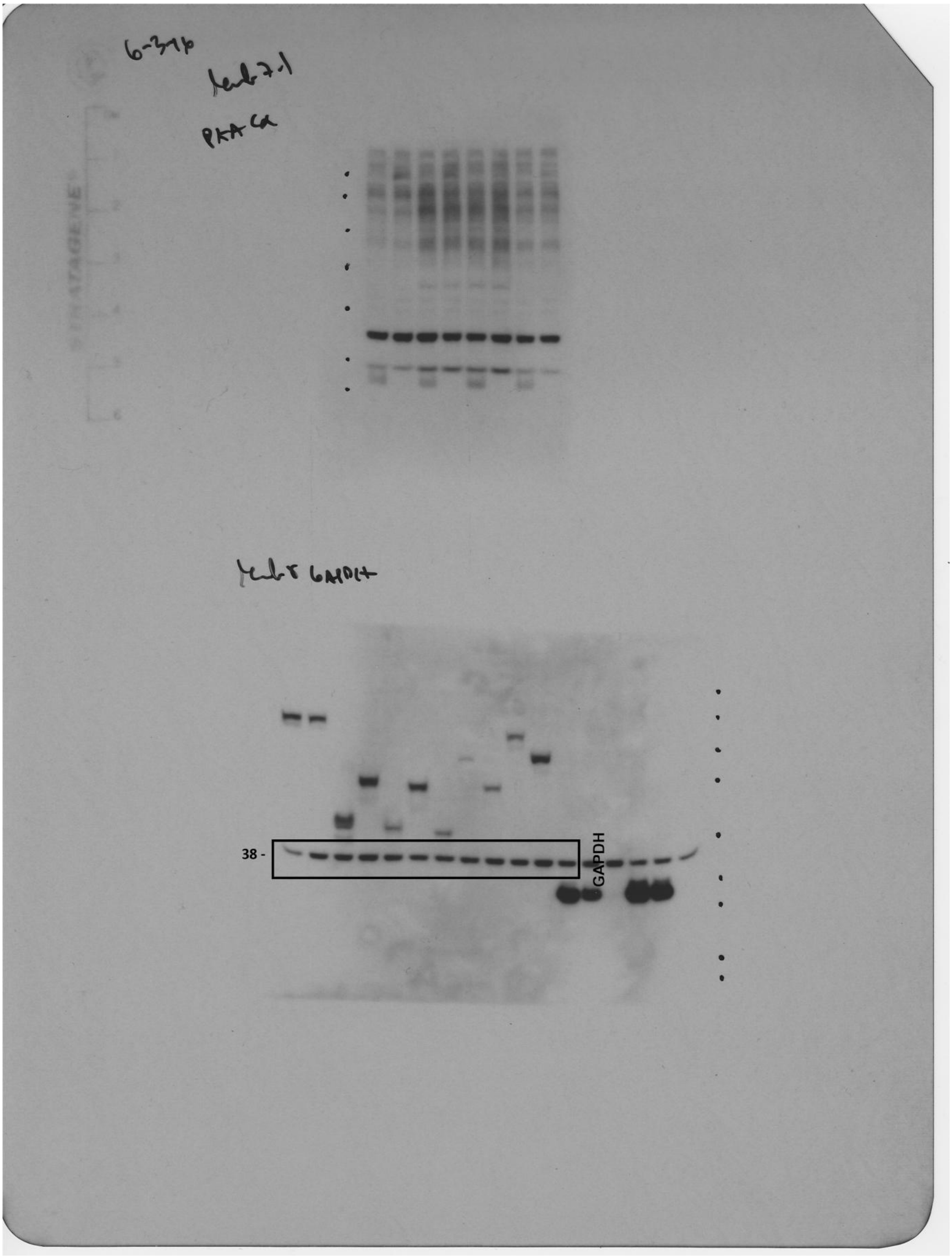

Supplemental figure 2d Mitochondria RFP (middle upper panel):

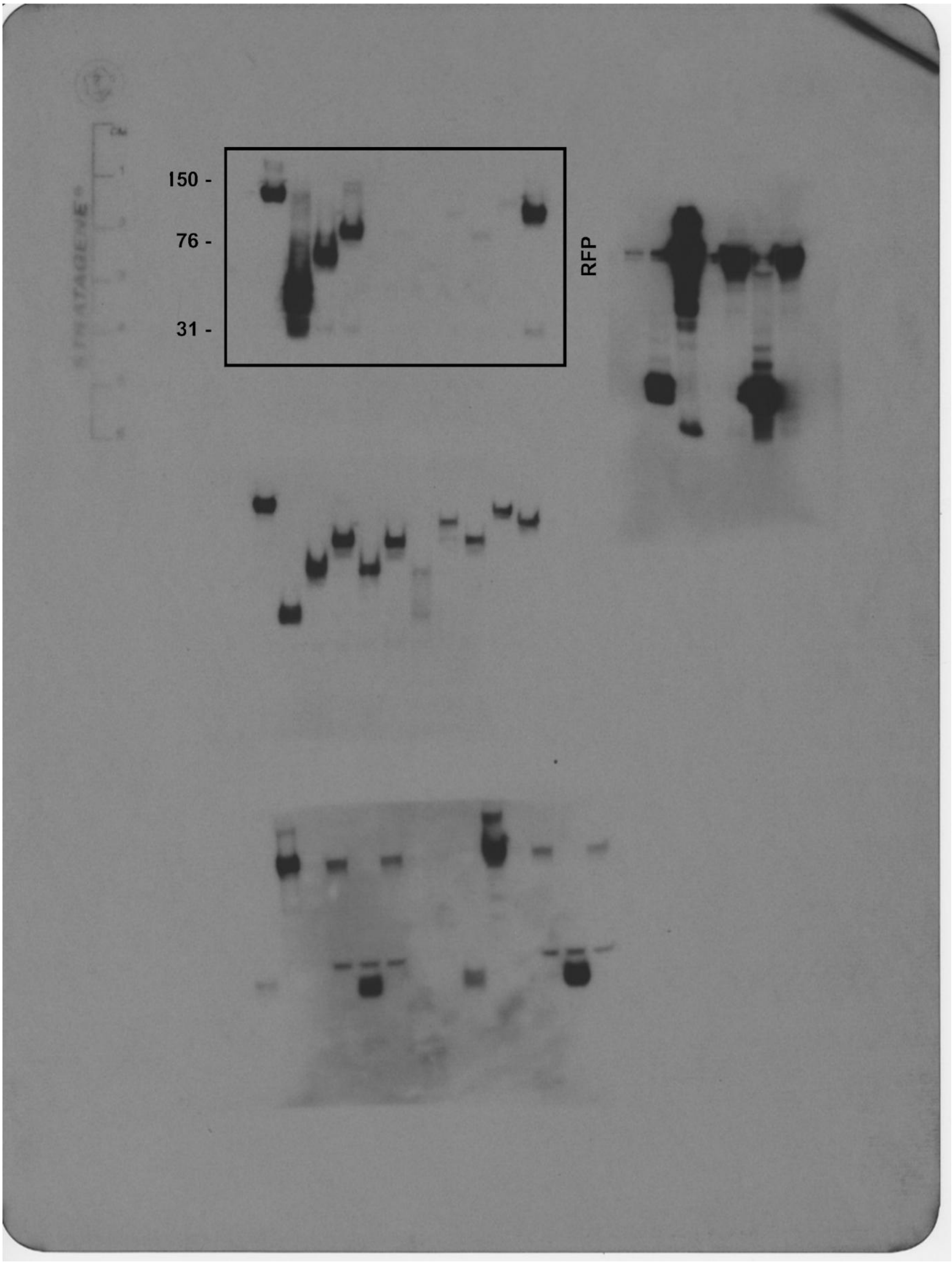

Supplemental figure 2d Mitochondria OXPHOS (middle lower panel):

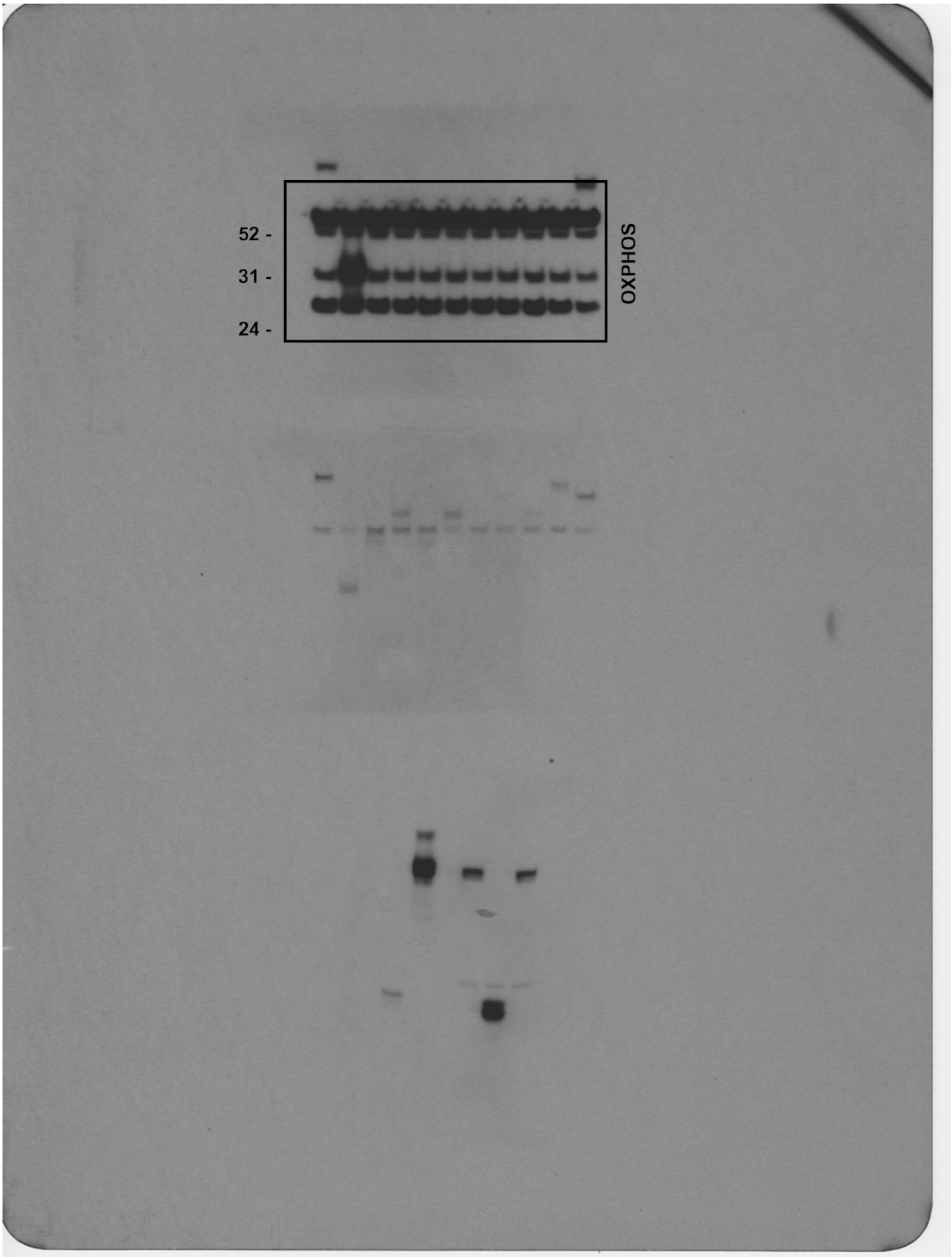

Supplemental figure 2d WCL RFP (right upper panel):

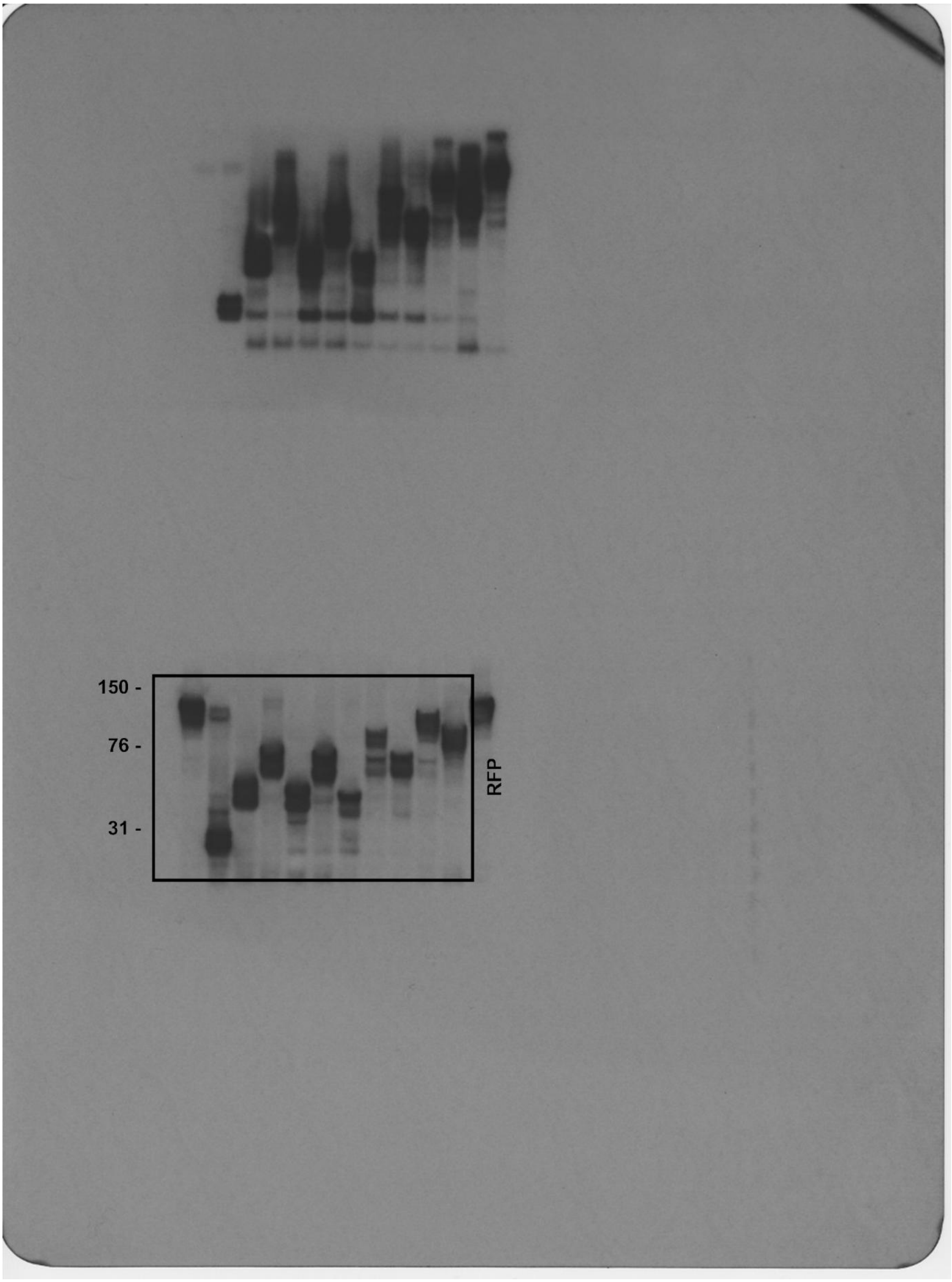

Original scans Supplemental Figure 3

Supplemental figure 3f Mitochondria RFP (upper panel):

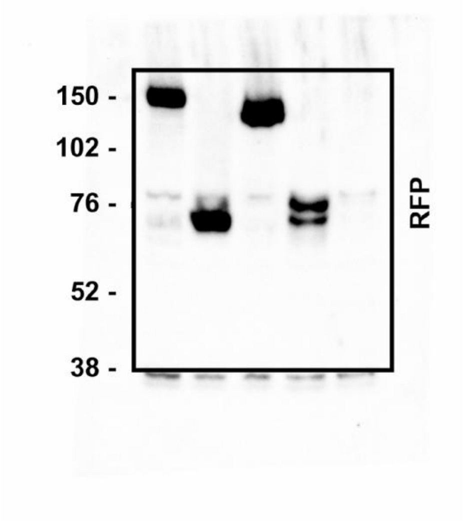

Supplemental figure 3f Mitochondria OXPHOS (lower panel):

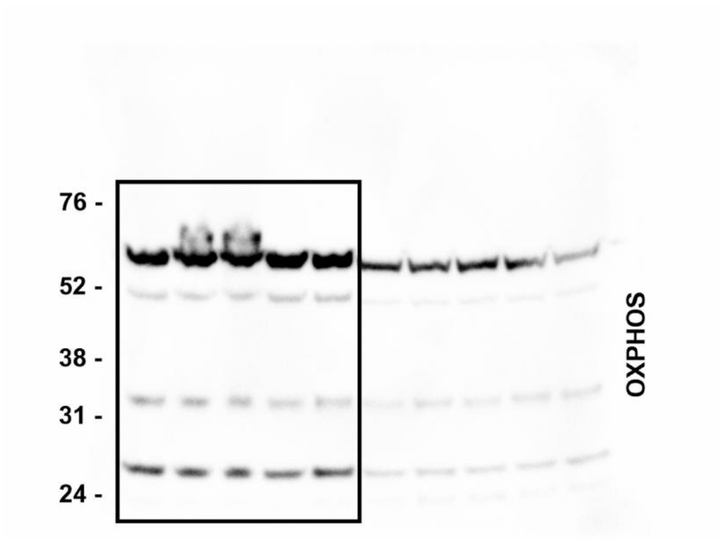

Supplemental figure 3f WCL RFP:

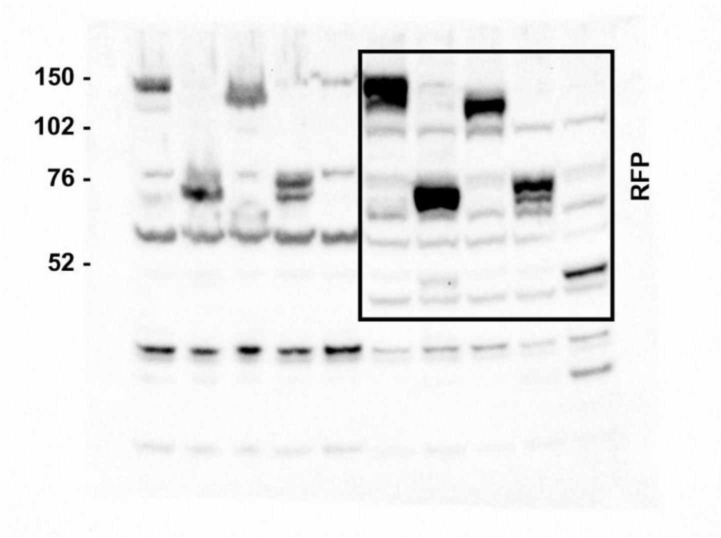

Original scans Supplemental Figure 4

Supplemental figure 4a RRx (upper panel):

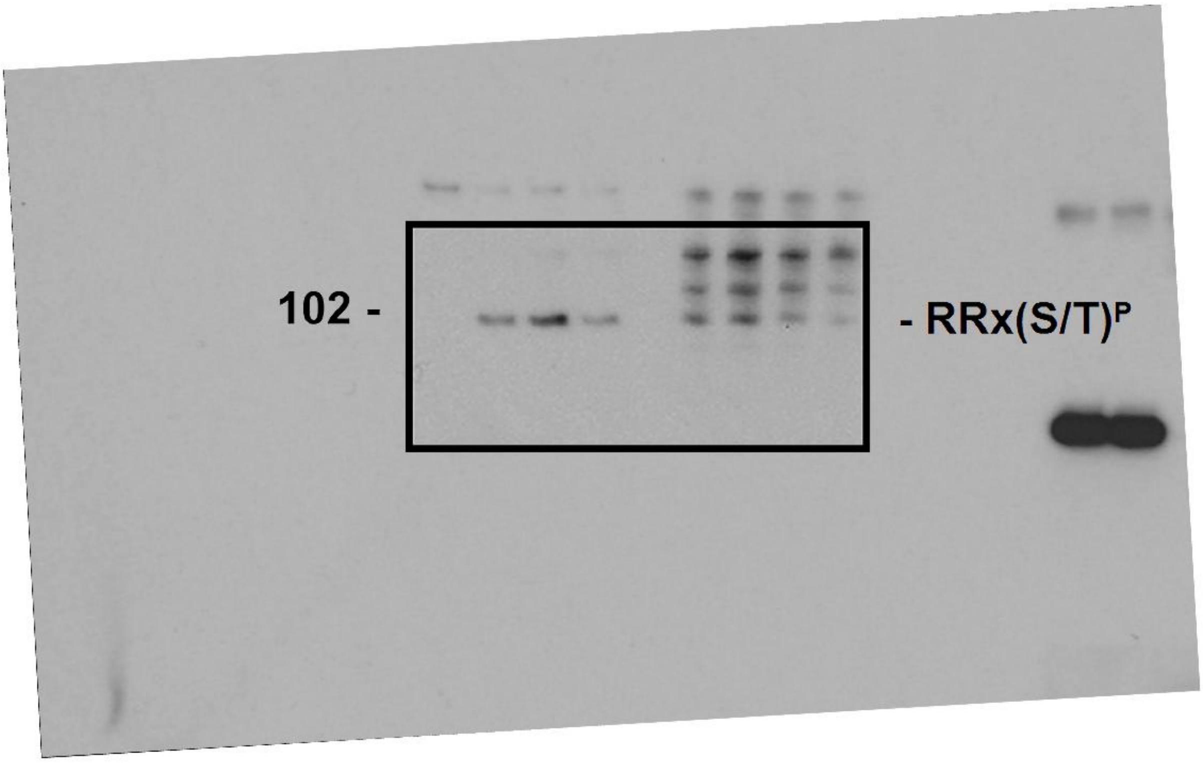

Supplemental figure 4a FLAG (middle panel):

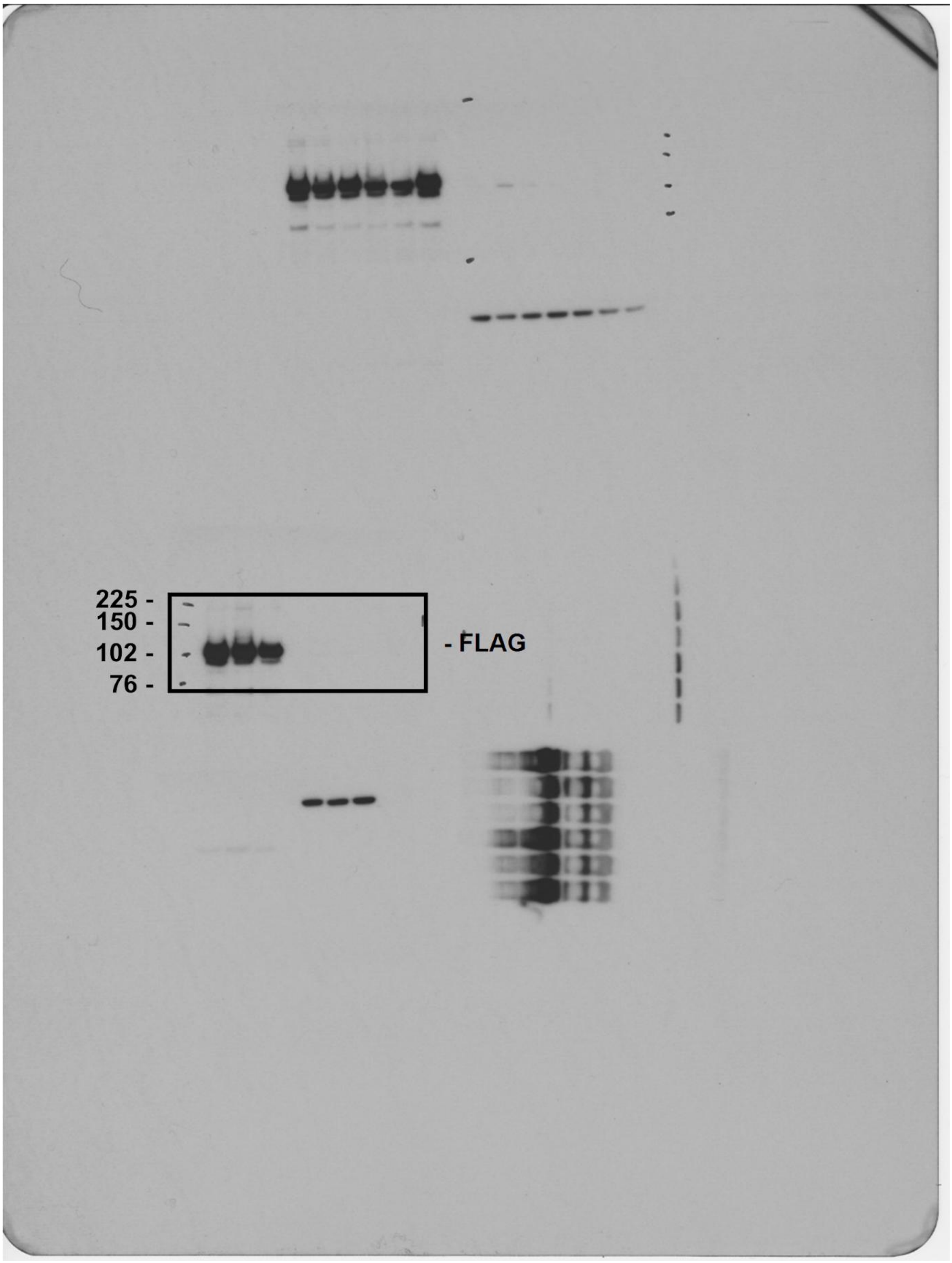

Supplemental figure 4a GAPDH (lower panel):

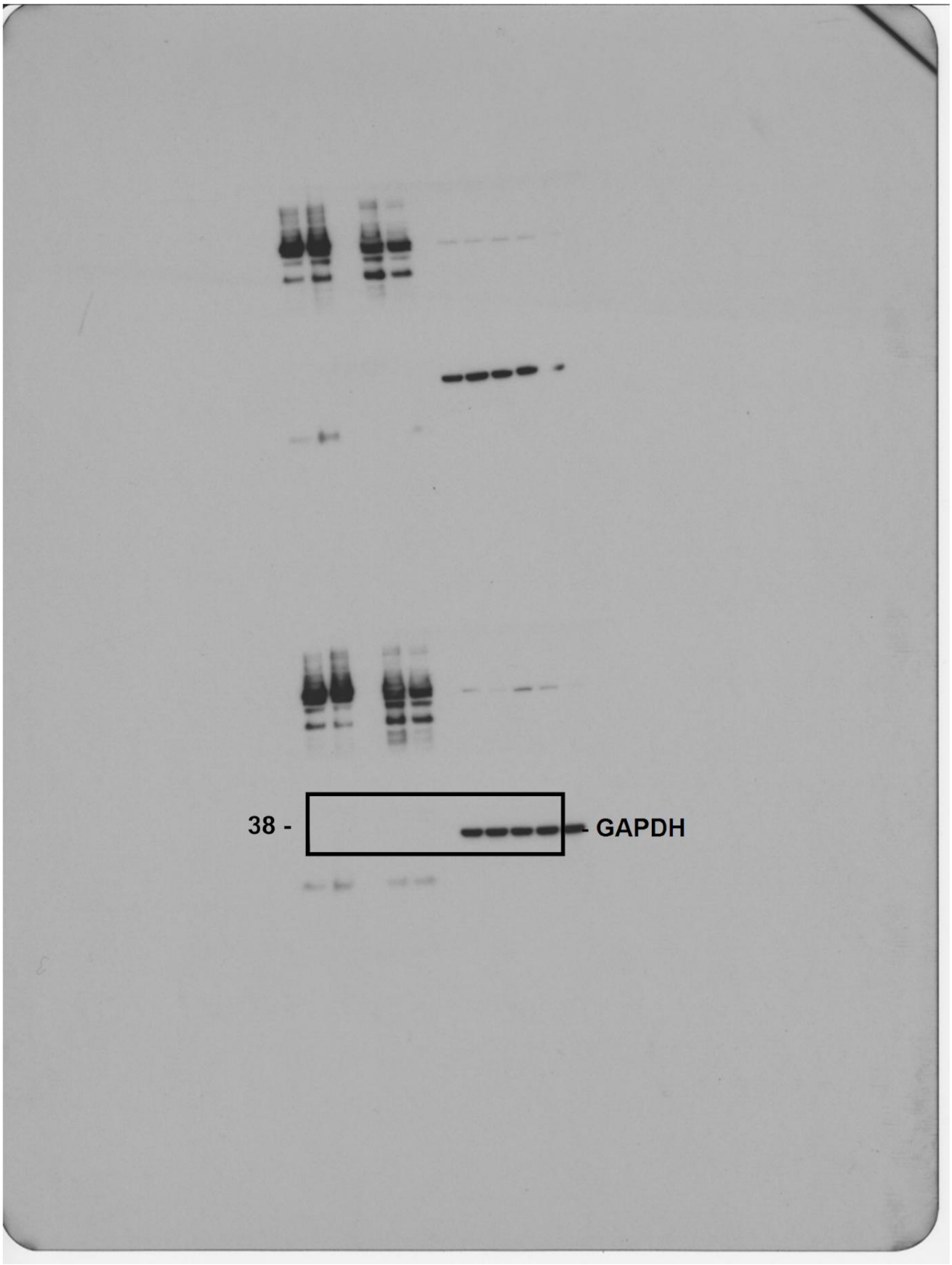

Supplemental figure 4c PDE2A2 (upper panel):

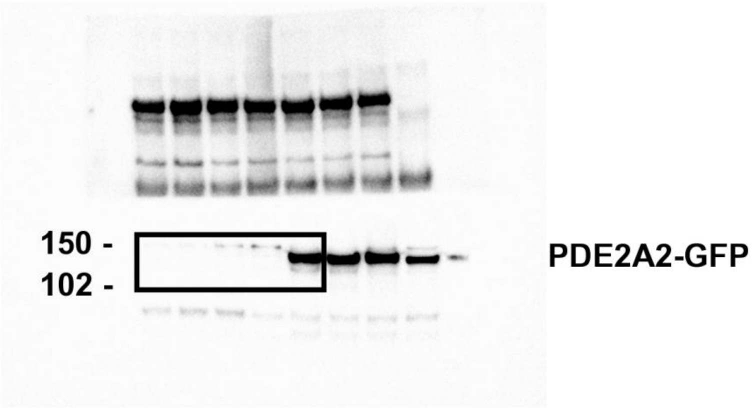

Supplemental figure 4c RRx (middle panel):

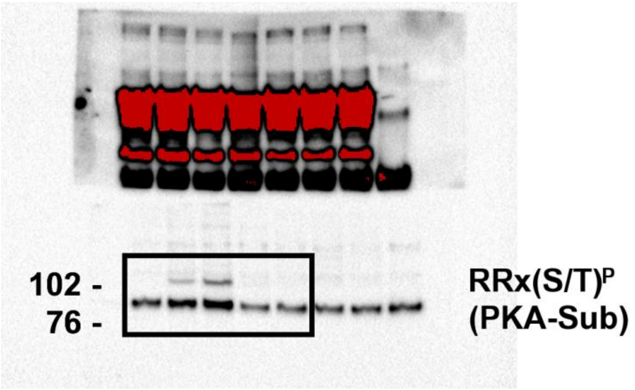

Supplemental figure 4c MIC60-FLAG (lower panel):

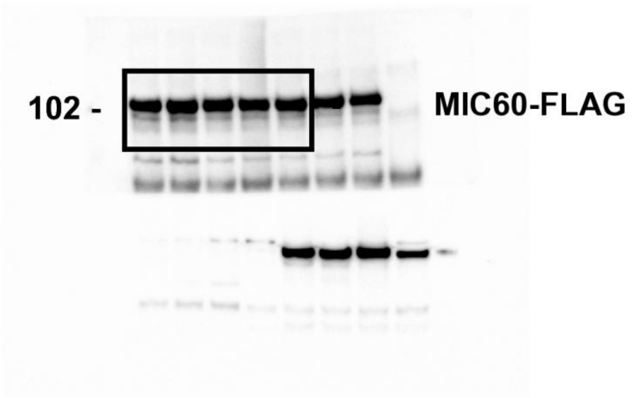

Supplement: Supplementary file 1 — Supplementary Information [file 42003_2020_1311_MOESM1_ESM.pdf]
