# Supplementary material for: Phosphodiesterase 2A2 regulates mitochondria clearance through Parkin-dependent mitophagy
Source: Commun Biol. 2020 Oct 21;3:596. doi: 10.1038/s42003-020-01311-7 (PMC7578833; doi:10.1038/s42003-020-01311-7)
Supplement: Supplementary file 2 — Description of Additional Supplementary Files [file 42003_2020_1311_MOESM2_ESM.pdf]

Description of Additional Supplementary Files

File Name: Supplementary Data 1
Description: Containing source data for the PDE2A2 interactome

File Name: Supplementary Data 2
Description: Containing the Fisher-test analysis for the PDE2A2 interactome

File Name: Supplementary Data 3
Description: Containing source data for all other experiments
