# Supplementary material for: Phosphodiesterase 2A2 regulates mitochondria clearance through Parkin-dependent mitophagy
Source: Commun Biol. 2020 Oct 21;3:596. doi: 10.1038/s42003-020-01311-7 (PMC7578833; doi:10.1038/s42003-020-01311-7)
Supplement: Supplementary file 6 — Reporting Summary [file 42003_2020_1311_MOESM6_ESM.pdf]

nature research

Corresponding author(s): Manuela Zaccolo

Last updated by author(s): August 20 2020

Reporting Summary

Nature Research wishes to improve the reproducibility of the work that we publish. This form provides structure for consistency and transparency in reporting. For further information on Nature Research policies, see Authors & Referees and the Editorial Policy Checklist .

Statistics

For all statistical analyses, confirm that the following items are present in the figure legend, table legend, main text, or Methods section.

n/a	Confirmed	
☐	☒	The exact sample size (*n*) for each experimental group/condition, given as a discrete number and unit of measurement
☐	☒	A statement on whether measurements were taken from distinct samples or whether the same sample was measured repeatedly
☐	☒	The statistical test(s) used AND whether they are one- or two-sided<br>*Only common tests should be described solely by name; describe more complex techniques in the Methods section.*
☐	☒	A description of all covariates tested
☐	☒	A description of any assumptions or corrections, such as tests of normality and adjustment for multiple comparisons
☐	☒	A full description of the statistical parameters including central tendency (e.g. means) or other basic estimates (e.g. regression coefficient) AND variation (e.g. standard deviation) or associated estimates of uncertainty (e.g. confidence intervals)
☐	☒	For null hypothesis testing, the test statistic (e.g. *F*, *t*, *r*) with confidence intervals, effect sizes, degrees of freedom and *P* value noted<br>*Give P values as exact values whenever suitable.*
☒	☐	For Bayesian analysis, information on the choice of priors and Markov chain Monte Carlo settings
☒	☐	For hierarchical and complex designs, identification of the appropriate level for tests and full reporting of outcomes
☒	☐	Estimates of effect sizes (e.g. Cohen's *d*, Pearson's *r*), indicating how they were calculated

Our web collection on statistics for biologists contains articles on many of the points above.

Software and code

Policy information about availability of computer code

Data collection	Mitophagy assessment was performed using ImageJ. For FRET imaging data were acquired using Metafluor software.
Data analysis	MS data were analyzed with the MaxQuant computational platform (version 1.2.0.11). Mitophagy analysis was performed using ImageJ. For FRET imaging data analysis we used Metafluor software.

For manuscripts utilizing custom algorithms or software that are central to the research but not yet described in published literature, software must be made available to editors/reviewers. We strongly encourage code deposition in a community repository (e.g. GitHub). See the Nature Research guidelines for submitting code & software for further information.

Data

Policy information about availability of data

All manuscripts must include a data availability statement. This statement should provide the following information, where applicable:

- Accession codes, unique identifiers, or web links for publicly available datasets
- A list of figures that have associated raw data
- A description of any restrictions on data availability

The PDE2A2 interactome raw data have been deposited and are available at ProteomeXchange (http://www.proteomexchange.org/)

Field-specific reporting

Please select the one below that is the best fit for your research. If you are not sure, read the appropriate sections before making your selection.

☒ Life sciences ☐ Behavioural & social sciences ☐ Ecological, evolutionary & environmental sciences

Life sciences study design

All studies must disclose on these points even when the disclosure is negative.

Sample size	By using average differences and standard deviations obtained in pilot studies and by performing a power calculation, sample size (n) has been calculated. For each protocol the number of animals per protocol is based on us achieving 80% power (alpha level of 0.05).
Data exclusions	No data were excluded
Replication	All experiments were replicated (biological replicates) at least three times and results were found to be consistent.
Randomization	Samples were allocated to a specific experimental group using one of the established randomisation techniques
Blinding	Investigators were blinded to sample allocation during data acquisition and analysis.

Reporting for specific materials, systems and methods

We require information from authors about some types of materials, experimental systems and methods used in many studies. Here, indicate whether each material, system or method listed is relevant to your study. If you are not sure if a list item applies to your research, read the appropriate section before selecting a response.

Materials & experimental systems

n/a	Involved in the study	
☐	☒	Antibodies
☐	☒	Eukaryotic cell lines
☒	☐	Palaeontology
☒	☐	Animals and other organisms
☒	☐	Human research participants
☒	☐	Clinical data

Methods

n/a	Involved in the study	
☒	☐	ChIP-seq
☒	☐	Flow cytometry
☒	☐	MRI-based neuroimaging

Antibodies

Antibodies used	FLAG (Cell Signaling, 8146; Cell Signaling,8146), GAPDH (SCBT, sc166574; Proteintech, 60004-1-Ig), GFP (SCBT, sc9996; Abcam,ab6556), MIC60 (Abcam, ab110329), Parkin (Cell Signaling, 4211), PDE2A (Proteintech,55306-1-AP), phospho-(Ser/Thr) PKA substrate (Cell Signaling, 2964), PINK1 (Cell Signaling,6946), RFP (Chromotek, 6G6; Abcam, ab62341), SAMM50 (Atlas Antibodies, HPA042935)
Validation	All antibodies used in this study have been extensively validated in numerous published studies

Eukaryotic cell lines

Policy information about cell lines

Cell line source(s)	HeLa, HEK293T and Sh-Sy5y were obtained from collaborators
Authentication	No authentication was performed
Mycoplasma contamination	All lines tested negative for mycoplasma
Commonly misidentified lines (See ICLAC register)	HEK293T. This line was used as the actual cell type is not relevant for this study
